# Serum and Serum Albumin Inhibit *in vitro* Formation of Neutrophil Extracellular Traps (NETs)

**DOI:** 10.3389/fimmu.2019.00012

**Published:** 2019-01-24

**Authors:** Elsa Neubert, Susanne N. Senger-Sander, Veit S. Manzke, Julia Busse, Elena Polo, Sophie E. F. Scheidmann, Michael P. Schön, Sebastian Kruss, Luise Erpenbeck

**Affiliations:** ^1^Department of Dermatology, Venereology and Allergology, University Medical Center Göttingen, Göttingen, Germany; ^2^Institute of Physical Chemistry, University of Göttingen, Göttingen, Germany; ^3^Lower Saxony Institute of Occupational Dermatology, University Medical Center Göttingen and University of Osnabrück, Göttingen, Germany

**Keywords:** neutrophils, neutrophil extracelluar traps, experimental conditions, NET, media, *in vitro* experiments, NETosis

## Abstract

The formation of neutrophil extracellular traps (NETs) is an immune defense mechanism of neutrophilic granulocytes. Moreover, it is also involved in the pathogenesis of autoimmune, inflammatory, and neoplastic diseases. For that reason, the process of NET formation (NETosis) is subject of intense ongoing research. *In vitro* approaches to quantify NET formation are commonly used and involve neutrophil stimulation with various activators such as phorbol 12-myristate 13-acetate (PMA), lipopolysaccharides (LPS), or calcium ionophores (CaI). However, the experimental conditions of these experiments, particularly the media and media supplements employed by different research groups, vary considerably, rendering comparisons of results difficult. Here, we present the first standardized investigation of the influence of different media supplements on NET formation *in vitro*. The addition of heat-inactivated (hi) fetal calf serum (FCS), 0.5% human serum albumin (HSA), or 0.5% bovine serum albumin (BSA) efficiently prevented NET formation of human neutrophils following stimulation with LPS and CaI, but not after stimulation with PMA. Thus, serum components such as HSA, BSA and hiFCS (at concentrations typically found in the literature) inhibit NET formation to different degrees, depending on the NETosis inducer used. In contrast, in murine neutrophils, NETosis was inhibited by FCS and BSA, regardless of the inducer employed. This shows that mouse and human neutrophils have different susceptibilities toward the inhibition of NETosis by albumin or serum components. Furthermore, we provide experimental evidence that albumin inhibits NETosis by scavenging activators such as LPS. We also put our results into the context of media supplements most commonly used in NET research. In experiments with human neutrophils, either FCS (0.5–10%), heat-inactivated (hiFCS, 0.1–10%) or human serum albumin (HSA, 0.05–2%) was commonly added to the medium. For murine neutrophils, serum-free medium was used in most cases for stimulation with LPS and CaI, reflecting the different sensitivities of human and murine neutrophils to media supplements. Thus, the choice of media supplements greatly determines the outcome of experiments on NET-formation, which must be taken into account in NETosis research.

## Introduction

The discovery of neutrophil extracellular traps (NETs) in 2004 (1) marked the beginning of an impressive scientific career of these extracellular DNA meshworks. NETs are expelled by neutrophilic granulocytes under certain (patho) physiological conditions. Different signaling pathways and forms of NET formation have been described in the last years (2, 3). In most studied scenarios, the cells release the NET consisting of decondensed chromatin, decorated with antimicrobial peptides and, most likely, a plethora of cytokines and other proteins. This release occurs after the rupture of the cell membrane into the extracellular space, ultimately leaving the neutrophil to die. This pathway has been called “NETosis” or “suicidal NETosis” (4), in analogy to previously known cell death pathways such as apoptosis and necrosis. In contrast, some publications have also described a faster, different form of NET formation mainly in response to bacteria which leaves the neutrophil alive and functional (“vital NETosis” or “vital NET formation”) (5). It remains a matter of debate whether these phenotypes are truly distinct biological processes.

While originally described as a novel immune defense mechanism to trap and kill pathogens like bacterial, fungi and even viruses, it has become increasingly clear that the role of NETs goes far beyond these initial discoveries. Indeed, excessive NET production or a dysregulation of NET clearance have been negatively implicated in an ever growing number of diseases, many of them associated with considerable morbidity and socioeconomic impact such as chronic inflammatory diseases like rheumatoid arthritis (6), systemic lupus erythematosus (7), chronic obstructive pulmonary disease (COPD) (8, 9) and psoriasis (10, 11), ischemia-reperfusion injury after myocardial infarction (12), thrombosis (13), impaired wound healing (14), preeclampsia (15), and cancer (16, 17). Therefore, it is not surprising that publications involving NETs have increased exponentially within the last couple of years and reliable methods to study NET formation are highly desired.

Initially, reports of NETs as contributors to different diseases relied mainly on the *ex vivo* detection of NETs and NET-related proteins by immunofluorescence and immunohistochemistry protocols in affected tissues. More recently, real-time observations of NETs forming *in vivo*, published in several very sophisticated mouse models, has led to great advances of our understanding of NETs and their role in different diseases. Similarly, flow cytometry-based protocols as a means for detecting NETs in blood from mice or humans have facilitated the screening for NET production in full blood under different pathological conditions (18, 19).

Nevertheless, *in vivo* models possess a high level of complexity, which does not allow the assessment of inhibitors and activators of NETosis in a high-throughput fashion and in a well-defined setting. Additionally, observing NETosis on a single-cell level remains very challenging in any *in vivo* setting. For example, testing the isolated influence of different stimuli such as bacterial proteins or inhibitors of certain neutrophilic enzymes on NETosis is hardly feasible *in vivo*; more so, if one aims to determine at which time-point of NETosis these activators or inhibitors play a role (20). Finally, *in vivo* studies (apart from the *ex vivo* assessment of existing NETs in peripheral blood by flow cytometry) are strictly limited to animal models. Currently it is not possible to determine whether neutrophils from patients with certain diseases show a greater propensity for NETosis unless one isolates neutrophils from these patients and stimulates them *ex vivo*. For these reasons, NET research heavily relies on the isolation of fresh neutrophils from donors and their *ex vivo* stimulation. Indeed, as of today, the isolation of neutrophils from patients, healthy donors, or animals followed by an *ex vivo* stimulation of these cells to assess and quantify NET formation is what may be called the gold standard of NET-experiments.

Considering the importance of this method for the whole field of neutrophil biology and the ever-growing number of laboratories performing NET studies, it is alarming to note that experimental conditions under which NETosis experiments are performed vary fundamentally from lab to lab, sometimes from publication to publication in the same group and occasionally even from assay to assay, rendering any comparison of research results nearly impossible. Looney at al. showed early on that the production of NETs by mice depend strongly on exterior influences on the mice. This group was one of the first to perform systematic NETosis experiments, in the context of transfusion-related acute lung injury (TRALI) (21, 22). Relocation of the mouse colony to a housing facility with a stronger barrier and less exposure of the mice to pathogens led to the inability of the group to repeat their own experiments—presumably, neutrophils from the new group of mice were not sufficiently pre-stimulated anymore. Only after exposure of the mice to low amounts of LPS prior to the experiments was the group able to recapitulate their previous results. This very instructive example shows how vulnerable NETosis is to external influences. One would expect neutrophils to be similarly or even more susceptible to variations of experimental conditions. Neutrophils are sensitive to subtle changes in the density and type of surface receptors (23), ranging from different methods of neutrophils isolation (24), type of activator used for the *ex vivo* stimulation of neutrophils [for a comprehensive review on activators of NETosis see also (25)] and kind of culture medium used to incubate neutrophils during NETosis. In human experiments, supplements added to culture media typically vary from no supplement to heat inactivated FCS (0.05 to 10% or 0.1 to 10%) and human serum albumin (HSA; 0.05 to 2 %), yet other supplements such as (heat inactivated) human plasma, (heat inactivated) human serum, and BSA can also be found at variable concentration (Supplementary Table 1, **Figure 5**). For murine neutrophils, the most frequently used supplements are FCS and BSA, though some activators of NETosis such as CaI and LPS are largely being studied in serum-free medium. These differences may greatly influence the outcome of NET-experiments, as albumin may bind proteins like lipopolysaccharides (LPS) (26, 27). Furthermore, an effect of supplements on *in vitro* NET formation has been considered from several groups. For instance, an inhibition of NET-formation was seen at very high FCS concentrations under Phorbol-12-myristate-13-acetate (PMA) in a dose-dependent way (4). Another group observed more cells involved in NET formation in serum-free medium after stimulation with nanoparticles (28), while others reported decreased NET-rates in HSA-containing media and pointed out, that a harmonization of culture conditions is still pending (4, 28, 29). Furthermore, it has been shown that serum-free culture conditions may allow a certain degree of spontaneous NET formation (30). Here, we selected the most commonly used supplements and analyzed the effect of their addition or omission on (suicidal) NETosis in a standardized setup. Additionally, we systematically compared media supplements for *in vitro* NETosis experiments in use in the literature.

## Materials and Methods

### Isolation of Human Neutrophils

Human neutrophils were isolated from venous blood of healthy donors. For all human studies, a pool of 15 healthy donors was available. For all experiments with human neutrophils, blood from at least 3 different donors from this pool was collected to isolate neutrophils. This study was carried out in accordance with the recommendations and with the approval of the Medical Ethics Committee of the University Medical Center Göttingen (UMG), protocol number 29/1/17 with written informed consent from all subjects and in accordance with the Declaration of Helsinki.

The isolation was performed under sterile conditions based on previously published protocols (31) using gradient centrifugation. In brief, fresh blood of healthy donors was collected in S-Monovettes EDTA (7.5 ml, Sarstedt) and immediately layered in a ratio of 1 to 1 on top of Histopaque 1119 (Sigma Aldrich). After centrifugation and washing with HBSS (without Ca^2+^/Mg^2+^) (Lonza) cells were separated a second time on a gradient consisting of 65, 70, 75, 80, and 85% of 10:1 diluted Percoll (GE Healthcare). Then, cells were washed and resuspended in 1 ml HBSS without Ca^2+^/Mg^2+^. Cellular identity and a purity of the isolated cells of >95% was confirmed by cytospin (Cytospin 2 Zentrifuge, Shanson) followed by Diff Quick staining (Medion Diagnostics).

### Isolation of Mouse Neutrophils

Mouse neutrophils were isolated from 6 to 10-week-old wild type C57BL/6J mice (male and female mice, equally distributed between groups). The blood was collected from the retroorbital venous plexus under full anesthesia with isoflurane and collected into the 15 mM EDTA (Gibco) containing BSA/PBS-solution. The isolation was performed according to previously published standard protocols (32, 33). After centrifugation, the cells were layered on top of a 10:1-diluted percoll gradient consisting of 78, 69, and 52% layers in PBS. Afterwards, erythrocytes were lysed with deionized water followed by a washing-step. Then, cells were resuspended in HBSS and cellular identity as well as purity >95% were confirmed as described above.

### Stimulation Assay

Freshly isolated human or murine neutrophils were counted and suspended in RPMI 1640 (Lonza) containing 10 mM HEPES (Roth) (RPMI/HEPES) and 0.5% HSA (Sigma-Aldrich), 0.5%/1%/2% FCS (Biochrom GmbH, Merck Millipore) or 0.5% BSA (Roth), respectively. FCS was heat inactivated at 56°C (Thermostat plus, Eppendorf, Hamburg) for 30 min before use. 10,000 cells per well were seeded in 96-glassbottom-well-plates (*in vitro* scientific) for 30 min (37°C, 5% CO_2_) and stimulated to undergo NET formation with either LPS from *Pseudomonas aeruginosa* (Sigma-Aldrich) at 10, 25, or 100 μg/ml, CaI (Sigma-Aldrich) at 4 μM, or PMA (Sigma-Aldrich) at 100 nM. After an incubation time of 3 h, cells were fixed with 2% PFA (Roth) to end NET formation and stored over night at 4°C. The fixed samples were washed with PBS (Sigma-Aldrich) and stained with 1.62 μM Hoechst (Sigma-Aldrich) for 15 min at room temperature. After staining, cells were washed and imaged by fluorescence microscopy (Axiovert 200, Zeiss; software: Metamorph 6.3r2., Molecular Devices) with the camera CoolSNAP ES (Photometrics). For each well, in total 5–6 images of clearly defined regions were obtained blinded in an automated fashion. For all experiments, the number of decondensed nuclei and the total cell counts were assessed using ImageJ (https://imagej.nih.gov/ij/download.html) and the percentage of decondensed nuclei/ NETs calculated by Excel (version: 14.3.0; Microsoft corporation).

### Immunofluorescence Staining

Human neutrophils were isolated, seeded (200,000/well) in 24-well plates on glass coverslips and activated to undergo NET formation as described above. After fixation with 2 % PFA (Roth) over night, cells were permeabilized 0.1 % TritonX (Merck) and incubated with 5 % FCS (Biochrom) to block unspecific antibody binding. Subsequently, cells were stained using monoclonal anti-human MPO (IgG, mouse) as primary antibody (Abcam, ab25989, 1:500) and polyclonal anti-mouse Alexa 555 (IgG, goat) as secondary antibody (Life technologies, A21422, 1:2000). Neutrophil DNA was stained with 1.62 μM Hoechst (Sigma-Aldrich) as described above. After the staining procedure, cells were stored protected from light at 4°C. Representative confocal fluorescence images were obtained with the olympus IX83 inverted microscope (software: Olympus Fluoview Ver.4.2, Olympus) and recorded 60x magnified (UPlanSApo 1.35 oil, Olympus). All pictures were recorded at equal exposure times for MPO to ensure comparability.

### Neutrophil Elastase NET Assays

1 × 10^6^ human neutrophils in RMPI/HEPES with or without 0.5% HSA were seeded in 24-well-plates and stimulated with LPS from *Pseudomonas aeruginosa* (100 μg/ml), CaI (4 μM) or PMA (100 nM) for 2–3 h at 37°C. Measurement of neutrophil elastase (NE) bound to extracellular neutrophil chromatin was carried out with the NETosis assays kit (Cayman) according to the company's instructions. In short, NETs were washed after stimulation to remove unbound NE, chromatin was decomposed by DNase and subsequently the activity of NE was measured in the supernatant by formation of the 4-nitroaniline product from a NE-substrate (N-methoxysuccinyl-Ala-Ala-Pro-Val p-nitroanilide). Absorption of the resulting product was measured at 405 nm (Thermo Scientific APPLISKAN® Software: Skanlt RE for Appliskan 2.3, Thermo Fisher Scientific). All measurements were carried out in duplicates.

### Anisotropy Measurements

Fluorescence anisotropy measurements were performed with a Fluoromax-4 spectrofluorometer (Horiba Scientific). All fluorescence emission spectra were recorded with excitation at 280 nm for BSA and 295 nm for HSA using excitation and emission slit widths of 5 nm. Emission detection wavelength was set at λ_em_ = 344 nm for BSA and λ_em_ = 350 nm for HSA, integration time was 1 s and detection steps were 1 nm. The cuvette used was a QS high precision cell (10x2 m; Hellma Analytics). First, anisotropy of 0.005% BSA, or 0.005% HSA was monitored for 500 s. Then, 10, 25, or 100 μg/ml LPS from pseudomonas aeruginosa were added to 0.005% BSA or 0.005% HSA for another 500 s. The system was allowed to reach equilibrium for 50 s.

### Calcium Measurements

Calcium concentrations in presents or absence of 0.5% HSA were determined in the medical laboratory of the University Medical Center Göttingen using standard protocols.

### Systematic Literature Review

For the literature review the online data base PubMed was used with the search terms “Neutrophil extracellular trap,” “NETosis” and “Neutrophil + NET” up to 1st of March 2018. We included 460 human and 108 murine *in vitro* NET studies published after 2004 with full access to the PDF and written in English. Reviews and exclusive *in vivo* studies were excluded. Moreover, we included only studies performed on murine or human neutrophils, not on other cell types, co-cultures, transfected cells or cell lines. This study required unequivocal primary information on the used medium and the performed stimulation method to be included in this work (Supplementary Figure 1). For comparison of spontaneous NET formation in serum-free culture conditions, we analyzed all human *in vitro* NET studies performed with neutrophils from healthy donors in media without addition of solvents or stimuli that reported relative spontaneous NETosis rates (29 out of 255 publications).

### Statistics and Data Analysis

All statistics were calculated with GraphPad Prism (Version 6.0, GraphPad Software Inc.). Significance was tested using standard two-way-ANOVA with Bonferroni's multiple comparisons test (ns, not significant; ^*^p < 0.05, ^**^p < 0.01, ^***^p < 0.001, ^****^p < 0.0001), after testing for normal distribution, where applicable. Fluorescence images were processed with ImageJ.46r (National Institutes of Health) and all cell counts obtained using the Plugin “Cell Counter”.

## Results

### Albumin and Serum Inhibit CaI- and LPS-, but Not PMA-Induced NETosis in Human Neutrophils

**Figure 1:**
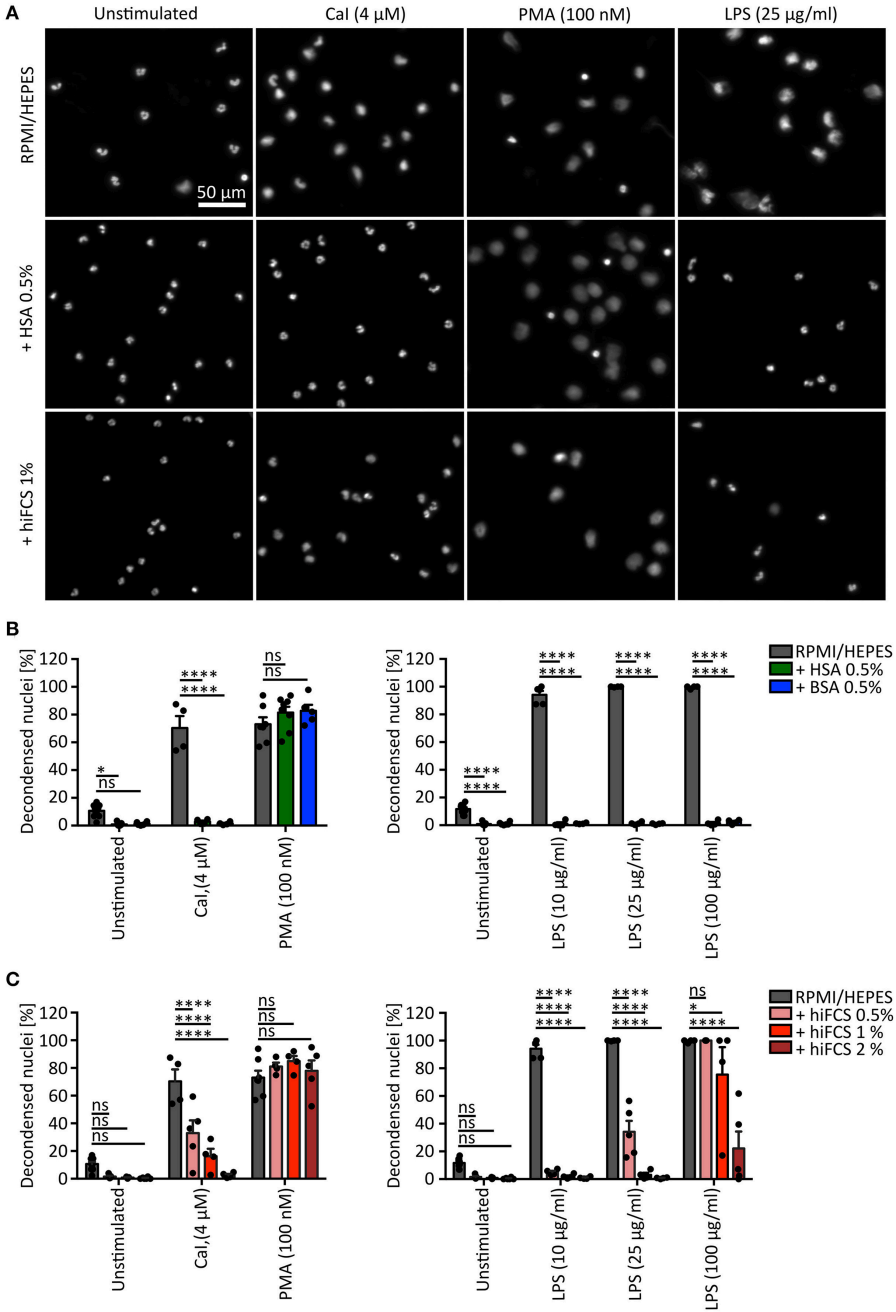
Influence of serum and serum albumin supplements on NET formation of human neutrophils. **(A)** Representative fluorescence images of human neutrophils (chromatin stained by Hoechst) after stimulation with CaI (4 μM), PMA (100 nM), or LPS (25 μg/ml) for 180 min, respectively. NET formation of neutrophils was studied in RPMI 1,640 with 10 mM HEPES (RPMI/HEPES), RPMI/ HEPES + 0.5% human serum albumin (HSA) or + 1% heat inactivated (56°C) fetal calf serum (hiFCS). Chromatin decondensation induced by PMA is clearly visible with all three culture conditions, while LPS or CaI only cause NET formation in BSA- and HSA-free RPMI/HEPES. Scale = 50 μm. **(B)** Neutrophils were stimulated to undergo NET formation with CaI (4 μM), PMA (100 nM) or LPS (10, 25, or 100 μg/ml), respectively. Both, HSA and BSA inhibit CaI and LPS-induced formation of NETs (determined as percentage of decondensed nuclei/NETs of total neutrophils). NETosis stimulated by PMA is independent of serum albumin addition. Error bars = mean ± SEM. ns, not significant. *p < 0.05, ****p < 0.0001. *N* = 4–9 [pool = 15 donors, 6–9 (unstimulated), 4 (CaI, 25 and 100 μg/ml LPS), 4–5 (10 μg/ml LPS), 5–8 (PMA)]. Two-way-ANOVA, Bonferroni's multiple comparisons test. **(C)** NET formation of neutrophils stimulated in RPMI/ HEPES supplemented with 0.5, 1, or 2% heat inactivated (56°C) fetal calf serum (hiFCS). Addition of hiFCS to RPMI/ HEPES decreases the percentage of decondensed nuclei/NETs after stimulation by CaI or LPS in a dose-dependent manner. PMA-induced NET formation occurs independently of FCS addition. Error bars = mean ± SEM. ns, not significant. *p < 0.05. ****p < 0.0001. *N* = 3–8 [pool = 15 donors, 5–8 (unstimulated), 3–5 (100 μg/ml LPS), 4–5 (PMA, CaI, 10 and 25 μg/ml LPS)]. Two-way-ANOVA, Bonferroni's multiple comparisons test.

**Figure 2:**
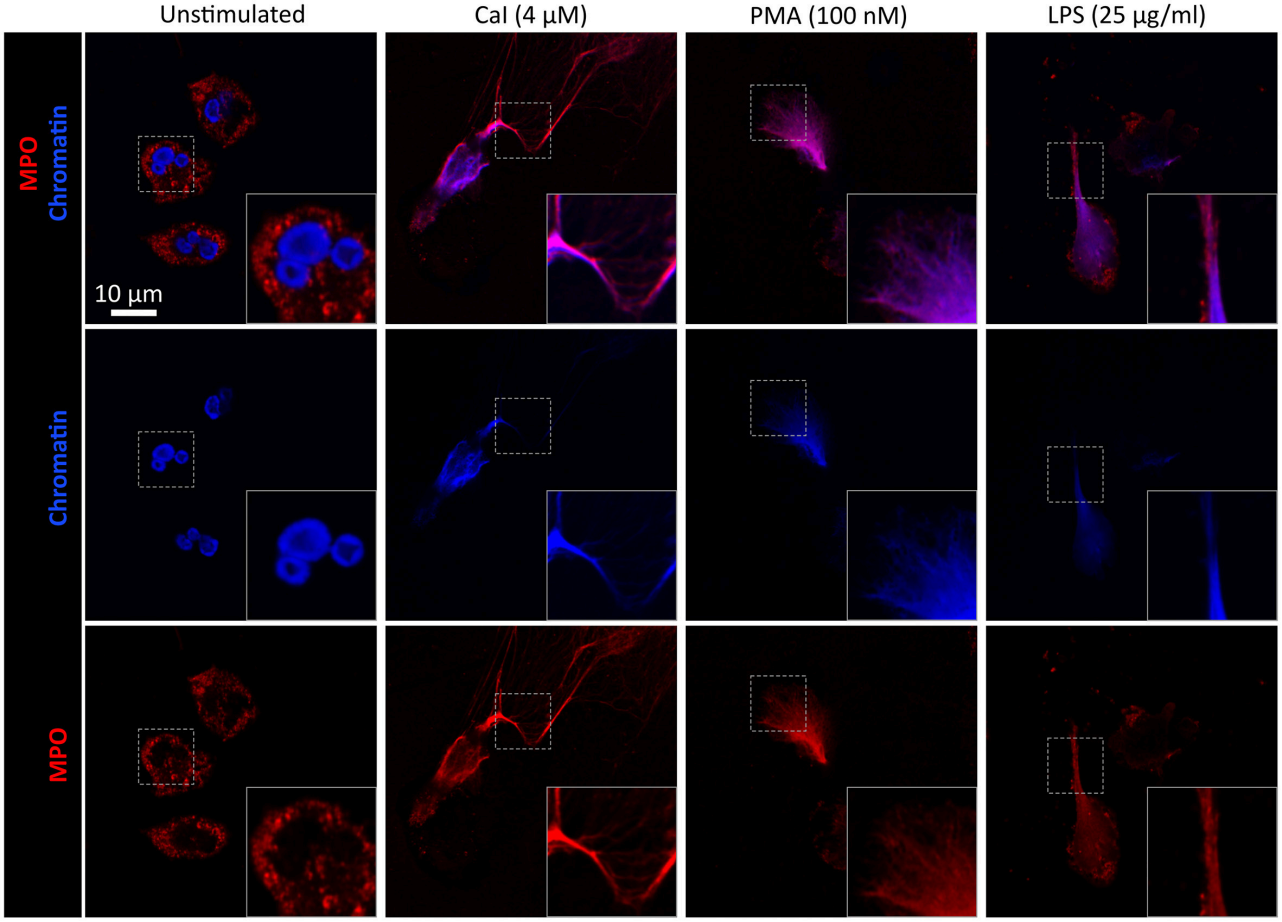
Extracellular NETotic DNA is rich in MPO. Representative fluorescence images of NETs induced by CaI (4 μM), PMA (100 nM), or LPS (25 μg/ml) activated in supplement-free RPMI/HEPES. The images show a clear colocalization of MPO (red) with the extracellular DNA-fibers (blue) of released NETs. Scale = 10 μm.

In the first series of experiments, we determined whether the different medium supplements have an influence on NET formation *in vitro*. To this end, we tested the frequently used supplements BSA, HSA and (hi)FCS at different concentrations on human neutrophils, taking into account the most commonly used concentrations in the literature (Supplementary Table 1). Firstly, we added FCS which had been heat-inactivated at 56°C (56°C hiFCS) to avoid side effects and degradation by serum nucleases, which is the most commonly applied manner of heat-inactivation. In 2009, von Köckritz-Blickwede et al. reported that even 56°C hiFCS may still contain heat-stable nucleases and recommended the use of FCS inactivated at 70°C (34). Nonetheless, we did not observe any difference in NETosis studied in 70°C hiFCS compared to 56°C hiFCS on the results in our setup (data not shown) and therefore decided for the more frequently used 56°C hiFCS for this study. We stimulated freshly isolated human neutrophils with CaI at 4 μM, PMA at 100 nM and LPS at 10 μg/ml, 25 μg/ml and 100 μg/ml to assess whether neutrophils stimulated by different inducers of NETosis would react differently to the media supplements (Figure 1). The identity of NETs was confirmed by co-staining of DNA and the neutrophil marker myeloperoxidase (MPO) (Figure 2) as well as release of neutrophil elastase (NE)-containing extracellular DNA (Supplementary Figure 2). The decondensed DNA of neutrophils having undergone NETosis in supplement-free RPMI/ HEPES clearly colocalized with MPO in confocal microscopy images after stimulation with CaI, PMA, or LPS. Furthermore, the NE-based NETosis assay showed the release of DNA-bound NE into the extracellular space after stimulation with the aforementioned stimuli, thus corroborating the induction of NETs in our experimental system.

We found that addition of BSA 0.5% as well as HSA 0.5% to RPMI/ HEPES significantly inhibited spontaneous NETosis and completely abolished NET formation mediated by CaI and LPS at 10, 25, and 100 μg/ml, whereas stimulation in pure RPMI/ HEPES led to a robust induction of NETs (Figures 1A,B). Interestingly, stimulation of neutrophils by 100 nM PMA was not influenced by either BSA or HSA (Figures 1A,B). In line with these observations, the release of DNA-bound NE after stimulation with CaI or LPS (100 μg/ml) was inhibited by addition of 0.5% HSA, but was not reduced in response to PMA (Supplementary Figure 2). Similarly, addition of hiFCS led to a dose-dependent decrease of CaI-induced NET formation but did not inhibit PMA-mediated NETosis (Figure 1C). Moreover, the addition of hiFCS significantly inhibited LPS-induced NETosis in a manner dependent on the concentration of both LPS and hiFCS itself. When 10 μg/ml LPS were used for the induction of NETosis, 0.5% hiFCS were sufficient to reduce NET formation dramatically from 95 to 5%. When 100 μg/ml LPS were employed, a significant reduction of NETosis to 22% could only be reached by adding 2% hiFCS (Figure 1C).

### Murine and Human Neutrophils React Differently to Media Supplements

**Figure 3:**
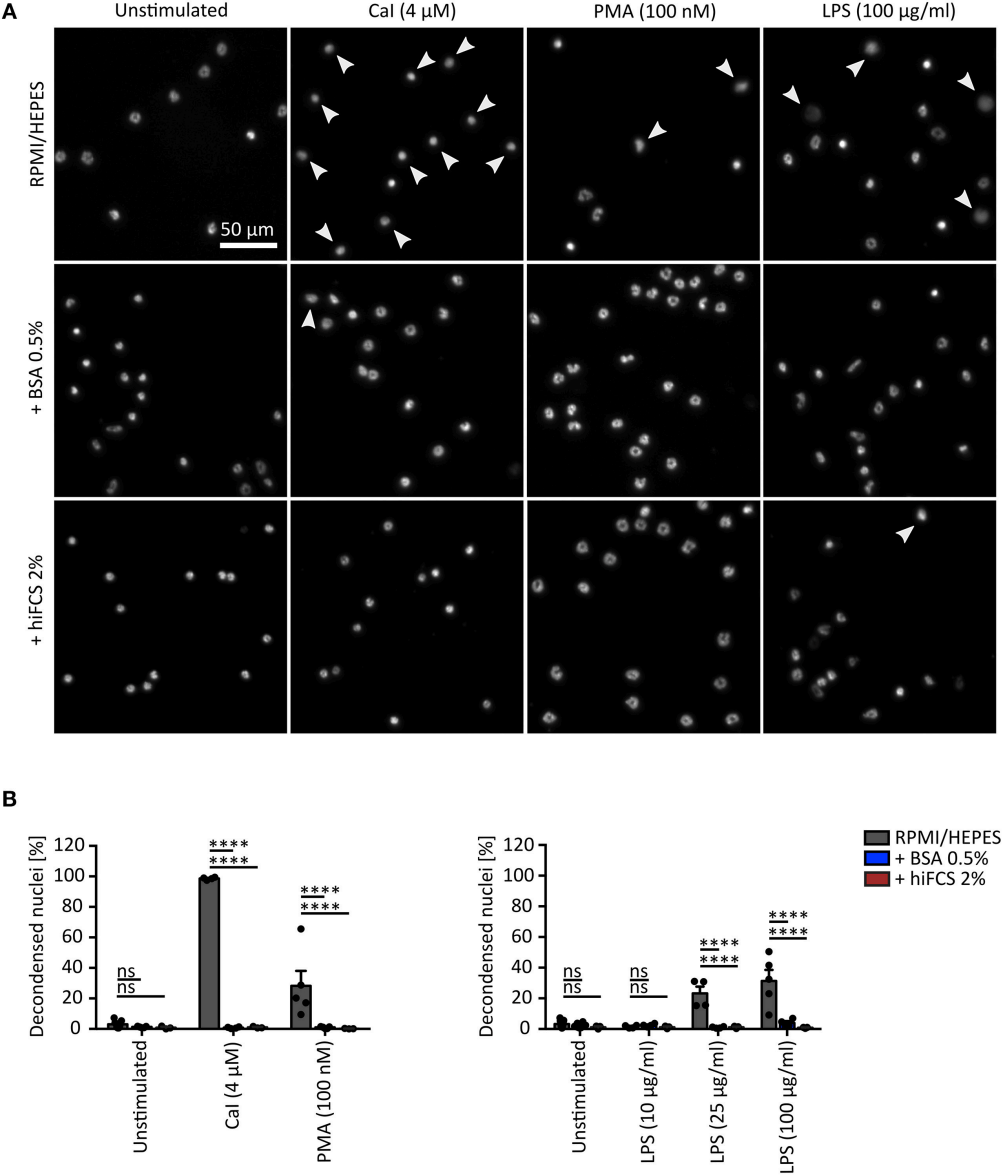
Influence of serum and serum albumin supplements on NET formation of murine neutrophils. **(A)** Representative fluorescence images of nuclei of murine neutrophils (Hoechst) after stimulation with CaI (4 μM), PMA (100 nM) or LPS (25 μg/ml) for 180 min, respectively. NET formation of neutrophils was studied in RPMI/ HEPES without supplements and RPMI/ HEPES supplemented with 0.5% BSA or 2% hiFCS, as indicated. Chromatin decondensation is only inducible in RPMI/ HEPES (white arrow heads). Scale = 50 μm. **(B)** Percentage of decondensed nuclei/ NETs after stimulation with PMA (100 nM), CaI (4 μM), or LPS (10, 25, or 100 μg/ml) for 180 min. Murine neutrophils were studied in RPMI/ HEPES and RPMI/ HEPES supplemented with 0.5% BSA or 2% hiFCS. Addition of 0.5% BSA or 2% hiFCS to RPMI/ HEPES, inhibits chromatin decondensation induced by PMA, CaI and LPS completely. Error bars = mean ± SEM. ns = not significant. ****p < 0.0001. *N* = 3–13 mice [3–13 (unstimulated), 3–5 (100 μg/ml LPS), 3–4 (CaI, 10 and 25 μg/ml LPS), 3–5 (PMA)]. two-way-ANOVA, Bonferroni's multiple comparisons test.

As many studies addressing NETosis are being conducted with murine neutrophils (Supplementary Table 2), we also sought to study whether media supplements such as FCS or BSA would influence murine NETosis in a similar way as described above for human neutrophils. Again, the three well established NET-inducers, CaI, PMA and LPS were used (Figure 3). This question was especially interesting in light of the fact that murine studies typically forego the addition of media supplements for selected stimuli as CaI and LPS, whereas human studies more often include either albumin or serum in the media (see also Figures 5, 6). While CaI was able to induce a strong response, causing almost 100% of neutrophils to undergo NETosis, PMA (at 100 nM) induced NETosis only in 28% of the neutrophils, which is in line with published results from the literature (4). Interestingly, the addition of 0.5% BSA or 2% 56°C hiFCS to RPMI/ HEPES was able to significantly inhibit both CaI- and PMA-induced NET-formation, contrary to what we had observed for human neutrophils. Similar results have been published for PMA-induced NETosis of murine neutrophils after the addition of hiFCS (4). For LPS from *Pseudomonas aeruginosa*, a minimum concentration of 25 μg/ml was needed to induce significant NET formation in murine neutrophils (23%) even without addition of media supplements. In general, LPS-induced NET formation was lower compared to that of human neutrophils at the same LPS concentration (Figure 3). However, similar to human neutrophils, addition of 0.5% BSA, or 2% hiFCS sufficed to completely inhibit LPS-induced NETosis.

### Human and Bovine Serum Albumin Bind LPS From *Pseudomonas aeruginosa*

As bovine and human albumin diminished NET-formation in most tested scenarios, we hypothesized that albumin, as part of its action in our setup, may bind part of the activators and thus lower their biologically active concentration. Indeed, it has already been shown that CaI binds to albumin (35, 36) and it appears likely that a similar mechanism exists for PMA.

**Figure 4:**
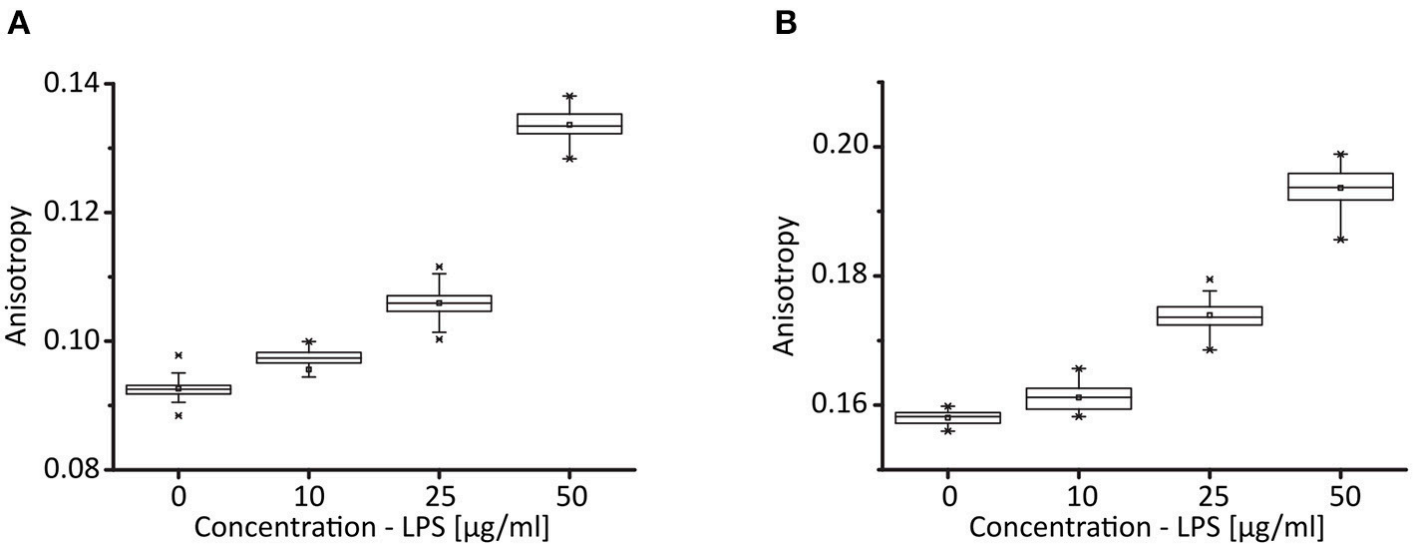
Binding of LPS to serum albumins. Box plots represent fluorescence anisotropy values for **(A)**, bovine serum albumin (0.005%) and **(B)**, human serum albumin (0.005%) after addition of LPS from Pseudomonas aeruginosa at 10, 25, or 50 μg/ml, as indicated. The anisotropy of albumin increases with higher LPS concentrations. For each concentration, the average anisotropy value (monitored for 500s) between two LPS additions is shown. Box plots show 25 and 75 percentiles with the midline as median, the square as arithmetic middle and crosses as 99%/1% values. Whiskers include all data points within the 1.5 interquartile range. *N* = 1.

To test the hypothesis that direct binding of activators to albumin diminishes NETosis, we performed fluorescence anisotropy measurements to quantify binding of LPS to albumin as an example of such an interaction. Fluorescence anisotropy measurements are a quantitative and standardized method to determine interactions between proteins and binding partners in solution (Figure 4). Solubilized albumin performs a certain rotational movement, which results in the emission of a fluorescence signal when the solution is excited by polarized light. The binding of molecules such as LPS to BSA or HSA causes this rotational diffusion to slow, which results in a measurable increase of fluorescence anisotropy. We used a concentration of 0.005% BSA (Figure 4A) or 0.005% HSA (Figure 4B), respectively, in our experimental system and added LPS at concentrations between 10 and 50 μg/ml. We found a dose-dependent increase of anisotropy with LPS, indicating that both BSA and HSA do indeed bind LPS. Even at concentrations of HSA and BSA that were lower than those used in our NETosis experiments, a clear binding between partners could be observed, which would be higher at higher HSA/BSA concentrations. Consequently, the presence of albumins in the medium, even at low concentrations, lowers the effective concentration of LPS, which explains the inhibiting effect in our *in vitro* setup. Due to the small size of PMA and CaI similar measurements could not be performed but it is likely that there is a similar binding as albumins are known to bind many different molecules (37–39), among them CaI, as indicated above (35, 36).

### Media Supplements Reveal Functional Differences Between Human and Mouse Neutrophils

We conducted a systematic literature research regarding human and mouse experiments studying NETosis *ex vivo/in vitro* to assess which supplements were commonly used by other groups working in the NETosis field (Supplementary Figure 1). Media supplements in studies dealing with human neutrophils (460 publications total) ranged from no supplement to FCS (0.05% to 10%), hiFCS (0.1 to 10%), human plasma (HP, 3 to 100%), hiHP (2 to 5%), human serum (HS, 0.2 to 100%), hiHS (0.1 to 100%) and serum albumin (HSA, 0.05 to 2%; BSA, 0.1 to 2%) (Supplementary Table 1). *Ex vivo* studies with murine neutrophils (108 publications) were carried out in media without supplements or medium supplemented with FCS (0.5 to 10%), hiFCS (0.1 to 10%), mouse serum (MS, 1 to 100%), BSA (0.1–2%) or in medium with HSA and bovine growth serum (BGS, 2%) (Supplementary Table 2).

**Figure 5:**
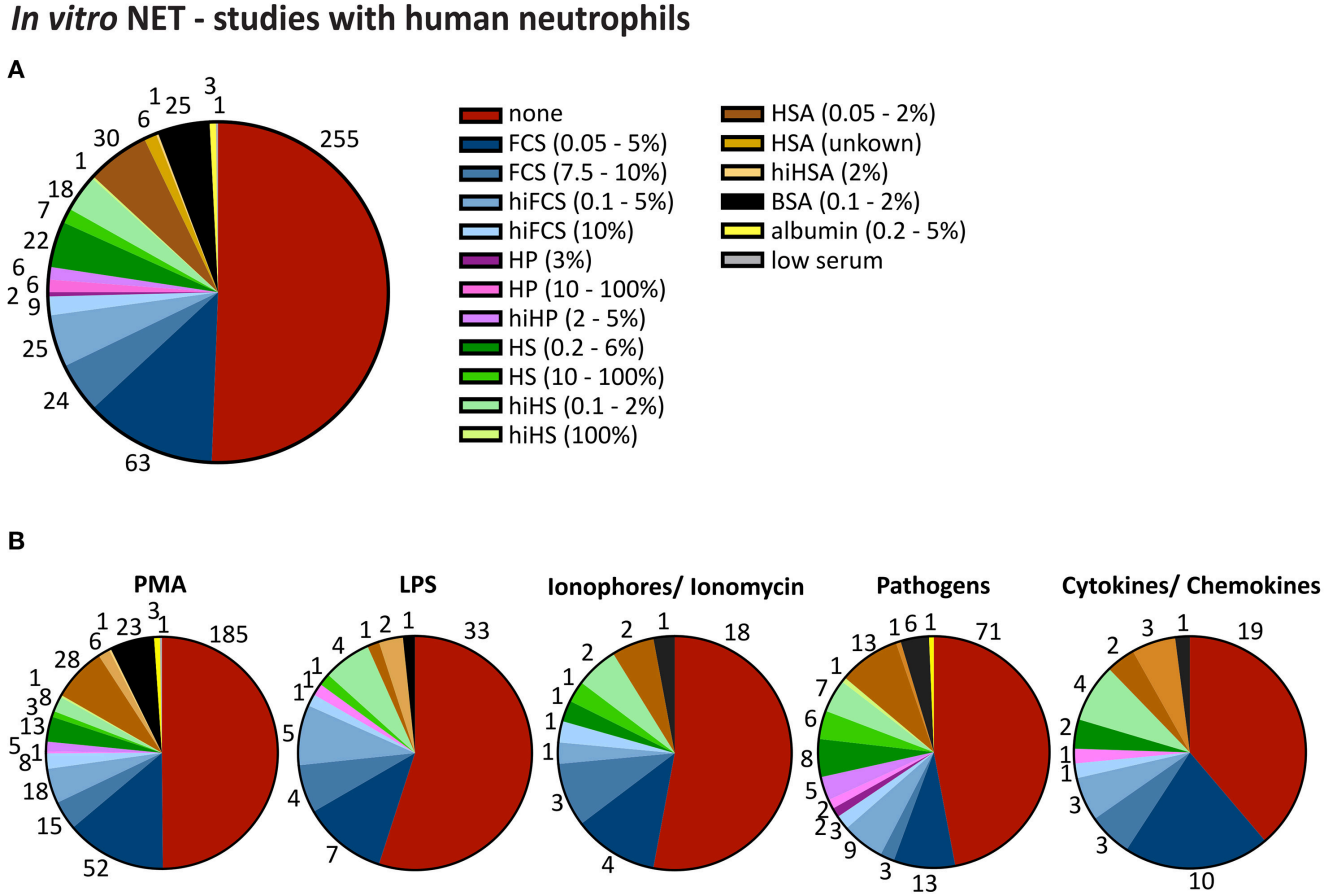
Heterogeneity in media supplements for human *in vitro* NETosis studies. Pie charts of serum, plasma, or serum albumin supplements used in human *in vitro* NETosis studies, as indicated (numbers represent the absolute numbers of publications using a certain supplement while the slices represent percentages of all considered publications). **(A)** chart for all stimuli combined (all publications were counted once). **(B)** charts for PMA, Ionophore/ Ionomycin, LPS, pathogen and cytokines/chemokines, respectively. Publications were counted for multiple activator-specific pie charts if more than one NET-activator was used. *N* = 460 publications up to the 1st of March 2018.

**Figure 6:**
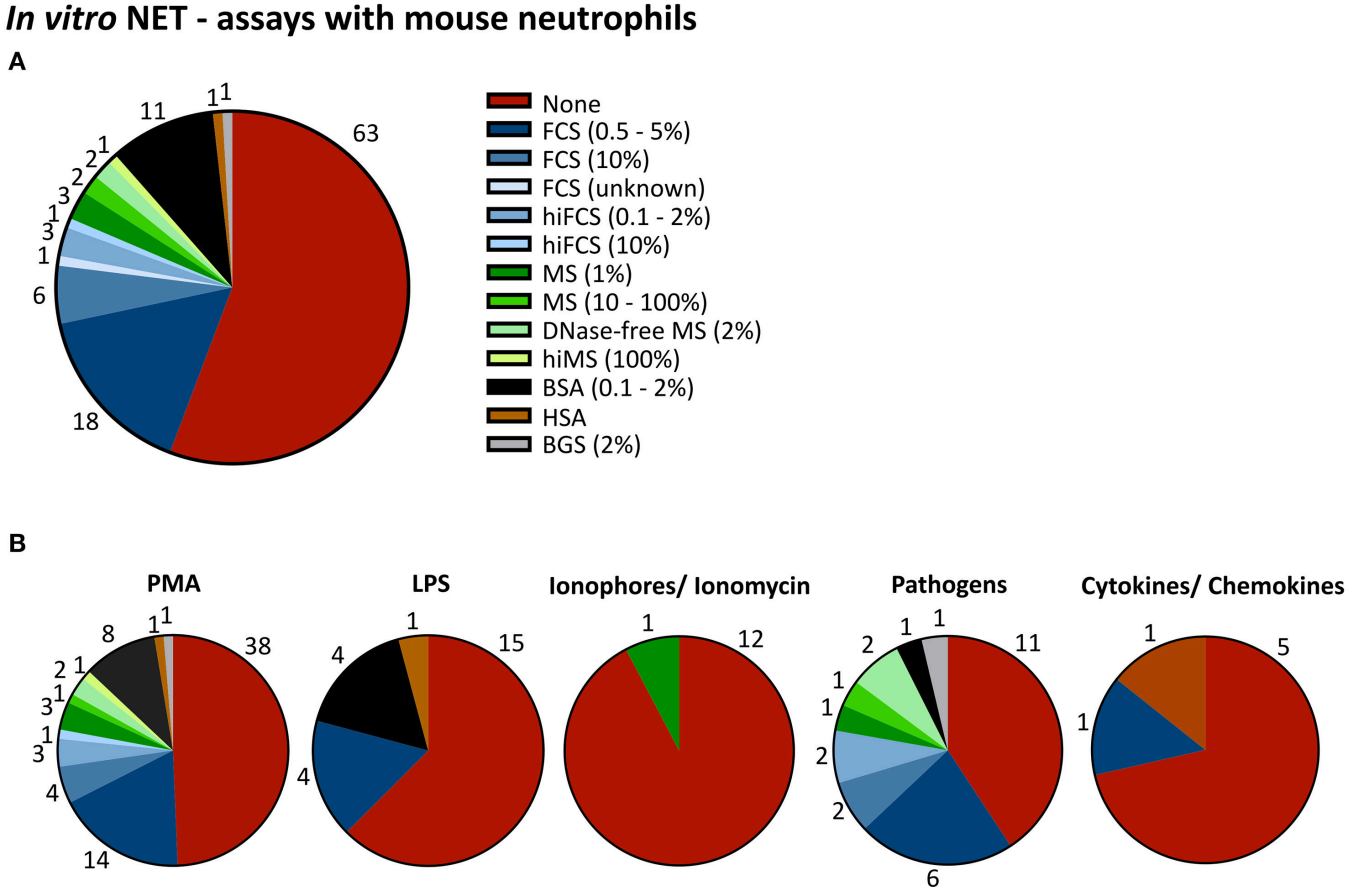
Heterogeneity in media supplements for murine *in vitro* NETosis studies. Pie charts of serum, plasma, or serum albumin supplements used in murine *in vitro* NETosis studies, as indicated (numbers represent the absolute numbers of publications using a certain supplement while the slices represent percentages of all considered publication). **(A)** chart for all stimuli combined. **(B)** charts for PMA, Ionophore/ Ionomycin, LPS, pathogen, and cytokines/chemokines, respectively. Publications were counted for multiple activator-specific pie charts if more than one NET-activator was used. *N* = 108 publications up to the 1st of March 2018.

Approximately half of the experiments in human (51 %) and mouse (56 %) neutrophils were performed without any supplement (Figures 5A, 6A) and the other half with very heterogeneous supplements. Additionally, even within the group that refrained from using media supplements, experimental conditions were overall quite diverse, as different basal media and different stimuli were used (see Supplementary Tables 1, 2).

As it is possible that the choice of media supplements is influenced by the activator of NETosis, we also plotted our extracted data into five groups according to the “major” NETosis stimulants (PMA, LPS, ionophores/ionomycin, pathogens, and cytokines/chemokines). Indeed, after this selection, certain activator-specific characteristics became apparent, such as the fact that for LPS, used in human studies, more than half of the publications forewent the use of media supplements, while in the publications using Ionophores or pathogens this approach was less common. Interestingly, studies using pathogens or cytokines and chemokines as inducers of NETosis generally showed the highest use of human plasma at different concentrations (Figure 5B).

In mice, the specific differences between experiments performed with different inhibitors were even more striking. While the use of PMA and pathogens led to a heterogeneous picture relatively similar to that seen with human neutrophils, LPS and ionophores were rarely or not at all employed in media-containing supplements (Figure 6B).

These striking differences led us to propose that media supplements might differentially influence different activators of NETosis and might lead to different results in human or mouse neutrophils. We would like to emphasize that in this analysis we did not differentiate between “vital” and “suicidal” NETosis and that we did not assess whether an assay successfully caused NET release or not. We included all manuscripts which clearly stated to study *in vitro* NETosis. Nonetheless, several of the included publications reported problems or failure to achieve a robust activation especially in media supplemented with serum or serum albumin. For instance, Wang et al. did not observe NETosis of TNFα-primed mouse neutrophils after stimulation with 100 ng/ml LPS in media containing 0.5% BSA and Papayannopoulos et al. clearly showed that peritoneal mouse neutrophils did not respond to PMA (100 nM) in media supplemented with 10 % FCS (40, 41). In *ex vivo* studies with human neutrophils several groups reported no or only a slight response to LPS in FCS containing media (Hoppenbrouwers et al.: 10% FCS, 5 μg/ml LPS; Barquero-Calvo et al.: 1% FCS, 0.7–100 μg/ml LPS; Hoffmann et al.: 2% FCS, 300 ng/ml LPS; Zhu et al.: 4% hiFCS, around 10% activation with 100 ng/ml LPS) or BSA (Ohbuchi et al.: 1% BSA, 10 ng−1 μg/ml LPS) (25, 29, 42–44) and Clark et al. did not observe any activation after stimulating human neutrophils with 5 μg/ml LPS in the presence of 10% plasma (45). As the publication of negative data is relatively rare, it is reasonable to assume the omission of media supplements in over half of the publications here analyzed indirectly reflects the difficulties of neutrophil stimulation in media containing albumin or serum supplements, at least for certain stimuli. In any case, the starting conditions of different studies are very divergent, which makes the publications in the field virtually impossible to compare, thus potentially diminishing the scientific significance and making the overall picture difficult to interpret.

## Discussion

NETosis and NET-related topics are currently intensely investigated topics with the number of publications steadily rising for the last couple of years. Despite the attention that the process of NETosis has attracted since its first publication in 2004 (1), little effort has been put into the standardization of experimental conditions for *in vitro* experiments. We are therefore advocating a consensus to define the experimental conditions as uniformly as possible in order to be able to better compare the results of different groups and better define important biological processes.

Previous sporadic publications had addressed the question of different culture media and media supplements such as FCS in an arbitrary manner (29), some of them hinting that indeed the addition of such components might greatly influence the outcome of NETosis experiments (4, 28–30). For this reason, we systematically tested the popular media supplements BSA, HSA and hiFCS and compared the effect of their addition to NETosis experiments without supplements. We used three of the currently best-established and most-used inducers of NET-formation, CaI, PMA, and LPS isolated from *Pseudomonas aeruginosa*. We chose these three activators because their mechanism of NET-induction has been well studied and they are known to involve different signaling cascades (46). Other popular inducers of NETosis include CXCL8 (IL-8) (15), reactive oxygen species such as H_2_O_2_ (4), activated platelets (47), microcrystals (48), *Aspergillus fumigatus* and *Candida albicans* hyphae (49) as well as live bacteria (1) [for a comprehensive review on activators of NETosis please see (25)].

We found that in media without albumin or serum components, CaI, PMA and LPS at all tested concentrations induced a strong and reproducible NET-formation in human neutrophils. On the other hand, spontaneous NETosis rates were relatively high in serum-free and serum-low conditions with a mean of 10.6% NET-rates. The increase of spontaneous NETosis rates in serum-free media is in line with results that have been reported in the literature (Supplementary Figure 3) (30).

The addition of BSA or HSA at 0.5% (which is at the lower range of concentrations used in the literature) led to an almost complete inhibition of NETosis induced by CaI or LPS. Thus, the experimental system appeared to be highly vulnerable to the influence of these media components. Astonishingly, PMA-induced NETosis remained unaffected by the addition of albumin (Figures 1A,B). Similarly, PMA-induced NETosis was not inhibited by any of the three tested concentrations of hiFCS (Figures 1A,C). This is in line with a plethora of publications that show robust induction of NETosis of human neutrophils by PMA in media containing albumin or serum components (14, 31, 50). We cannot exclude an inhibitory effect of FCS on PMA-induced NETosis at very high FCS concentrations, as has been shown by Fuchs et al. who used up to 20% FCS to inhibit NETosis (4), however we refrained from reproducing this setup as such high FCS concentrations are rarely used in the literature.

In contrast, CaI-induced and LPS-induced NETosis were inhibited not only by BSA or HSA, as discussed above, but also by hiFCS. Again, this corroborates previously published data which has described LPS as a poor and unreliable inducer of NETosis when using media complemented with FCS or BSA (25, 29, 42–44, 51). It is interesting to note that for human neutrophils, which are often cultured in media containing either albumin or serum components, LPS is used as an inducer of NETosis more rarely than in mouse studies, which are carried out without media supplements in the majority of cases (52). This becomes especially obvious in publications using both human and mouse neutrophils (14, 53). On the other hand, many human studies which do employ LPS as an inducer use serum-free media (54–57) (see also Supplementary Table 1). The reason for these observations may be that LPS, even at high concentrations, fails to adequately activate neutrophils to produce NETs in the presence of BSA, HSA or hiFCS (Figure 1C). A possible explanation for this phenomenon is a strong binding of LPS by large proteins such as albumin (Figure 4) and, very likely, also by other serum components. Additionally, NETosis induction by LPS relies heavily on integrin receptors such as MAC-1 (58) and this outside-in signaling may be influenced by the addition of large proteins and certainly also of serum components which contain a mixture of active components that can engage integrin receptors. In contrast, PMA is a direct activator of protein kinase C (PKC) (59), a vital and relatively far downstream step in most pathways leading to NETosis. Therefore, outside-in signaling could be expected to be less crucial during the activation cascade, at least for human neutrophils.

However, it is important to bear in mind that the isolation and stimulation of neutrophils *ex vivo* is a complex process, which depends on many factors apart from the used media and media supplements. It is likely that the method of neutrophil isolation, activation as well as the predisposition of the donor will also influence whether stimulation of neutrophils is successful or not. To minimize side-effects of the isolation method such as pre-activation of the used neutrophils, we decided to employ well characterized density gradient methods for neutrophil isolation (14, 33). Gradient-based isolation technics are one of the most commonly used methods in NETosis-studies. Compared to isolation by polymorphprep^TM^ or combinations with lysis of erythrocytes, pure gradients reveal comparably low spontaneous NET formation, as reported in the literature and corroborated in our own results (Supplementary Figure 3). Furthermore, the used anticoagulant can significantly influence pre-activation, calcium levels, and number of obtained neutrophils (60). For instance, heparin anticoagulation can influence the expression of adhesion molecules (61). For this reason, we have employed EDTA as an anticoagulant in this study (60).

Additionally, for LPS also the bacterial source has a significant impact on the success of stimulation and concentrations needed for this assay (62). Therefore, it is not surprising that concentrations of LPS used for *ex vivo* studies differ widely and, in some cases, LPS-induced NETosis occurred even in media containing albumin or serum components (63–65) or occasionally revealed only low NET-rates in supplement-free media (66, 67).

For mouse neutrophils, PMA-induced NETosis was, surprisingly, strongly inhibited by addition of 0.5% BSA and 2% hiFCS. In general, mouse neutrophils appear to be harder to activate than human neutrophils, they require higher threshold concentrations for LPS and produce less NETs upon PMA activation. One may speculate that mouse neutrophils may need a higher concentration of PMA to reach the same threshold of activation and that a minimal scavenging of available PMA or a certain unspecific “stabilizing effect” of BSA/HSA would thus affect murine NETosis more profoundly than it would for human cells. This would be in line with the data shown by Fuchs et al. (4) for the inhibitory effects of high concentrations of FCS (up to 20%) on PMA-activated human neutrophils: If human neutrophils required much lower concentrations of PMA to still perform NETosis, then a considerable “scavenging” effect of the activator would be of consequence only at high FCS concentrations.

Another possible explanation that must be taken into account for the differences seen between inductors of NETosis in response to media supplements is a possible influence on extracellular calcium levels. Calcium has been shown to be bound by albumin (68, 69) and appears to be essential for NETosis activation by PMA and, to a lesser extent, for CaI (46, 70). However, in our experimental setup we could not observe a significant influence of HSA on calcium levels (Supplementary Figure 4) and doubling of calcium-concentration (to 0.8 mM) did not reverse the inhibitory effect of HSA in our hands (data not shown).

Concerning the differences seen between PMA-induced NETosis in humans and mice, it must be taken into account that PMA-induced NETosis in mice may in part rely on different biochemical pathways in these two different species. For example, for murine neutrophils an involvement of enzymes such as PAD4 has been reported previously (71), whereas the involvement of PAD4 in PMA-induced human NETosis is still under discussion (46, 72). A similar observation, namely that decondensation of chromatin and NETosis in human neutrophils depends on PAD4 function to a lesser extent, was reported for *Staphylococcus* (73). In the context of different human diseases, a number of endogenous and receptor-mediated, inflammatory pathways have been identified that may lead to and regulate NET formation. For instance, in COPD the chemokine IL-8 (CXCL-8) and signaling through the CXCR2 receptor are thought to be involved in the disease (74) and in lupus nephritis, NET formation is thought to be regulated by engagement of the uniquely human FcγRIIA by soluble immune complexes (75). It remains to be explored how these pathways differ in humans and mice and how environmental conditions may influence them.

The effect of media supplements on NETosis experiments is most likely a complex one, however one may speculate that a great part of this effect is brought about by binding of the activators to albumin or to other serum proteins. In fact, for LPS and CaI binding to albumin has already been reported, and we here confirm and exemplify the binding of LPS to albumin by fluorescence anisotropy measurements. Indeed, binding of toxic and immune-activating substances like LPS or chemoattractants is likely a mechanism to protect the host from an overactive immune system and dysregulated NETosis, which has been shown to have deleterious effects (76–78). Additionally, serum even contains active DNases which are able to degrade already released NETs, which also shows that the composition of the environment in which neutrophils become activated are part of an immune regulation to limit excess inflammation (79).

On the other hand, serum condition in the tissue to which neutrophils are recruited in case of an inflammation to fight pathogens might be different from those of the blood, i.e., serum composition in the blood is arguably different from that in solid tissues. It is thus likely that, in part, a different grade of inhibition of neutrophil function under different conditions directs neutrophil behavior and orchestrates immune responses (30).

Our experimental results are indirectly reflected in the literature of experiments studying NETosis. While generally there is a plethora of different experimental conditions being employed in the field, it is striking to note the differences between human and mouse experiments and particularly in the experimental conditions used when LPS or CaI are employed as activators. Here, the vast majority of all publications refrained from adding any culture supplements, which is in line with our observation that CaI- and LPS-induced NETosis are very strongly inhibited by albumin.

Thus, the choice of media supplements greatly determines the outcome of *in vitro* experiments on NET-formation, which must be considered when planning and comparing NET-studies. Studies using different media settings may not be comparable and in general the question must be asked to which extent experiments omitting media supplements are actually recapitulating the natural function of neutrophils and NETs. On the other side, *in vivo* compartments are also different and differ in concentrations of chemokines, adhesion receptors, and other cells. Therefore, the questions we addressed in this paper is not restricted to *in vitro* experiments but also highly relevant for the assessment of *in vivo* experiments.

A standardization of protocols for NET experiments would allow for a better comparison between results from different groups.

## Author Contributions

EN and SS-S performed most experiments, co-wrote the paper, and designed the study together with LE. VM, JB, and SS performed experiments and analyzed data. EP provided the anisotropy data, supervised by SK. SK and MS provided crucial technical and scientific input and helped design the study. LE designed and supervised all experiments and co-wrote the paper. All authors critically proofread and edited the manuscript.

### Conflict of Interest Statement

The authors declare that the research was conducted in the absence of any commercial or financial relationships that could be construed as a potential conflict of interest.
