# Supplementary material for: Serum and Serum Albumin Inhibit in vitro Formation of Neutrophil Extracellular Traps (NETs)
Source: Front Immunol. 2019 Jan 24;10:12. doi: 10.3389/fimmu.2019.00012 (PMC6354573; doi:10.3389/fimmu.2019.00012)
Supplement: Supplementary file 1 [file Data_Sheet_1.docx]

**Supplementary Information**

**In vitro formation of Neutrophil Extracellular Traps (NETs) is inhibited by the presence of serum and serum albumin**

E. Neubert^1+^, S. Senger-Sander^1+^, V. Manzke^1^, J. Grandke^1^, E. Polo^2^, S.E.F. Scheidmann^1^, M.P. Schön^1,3^, S. Kruss^2^ and L. Erpenbeck^1^*

+ contributed equally

* corresponding author

*^1^Department of Dermatology, Venereology and Allergology, University Medical Center Goettingen, Germany*

*^2^Institute of Physical Chemistry, University of Goettingen, Germany*

*^3^Lower Saxony Institute of Occupational Dermatology, University Medical Center Goettingen and University of Osnabrueck, Germany*

*
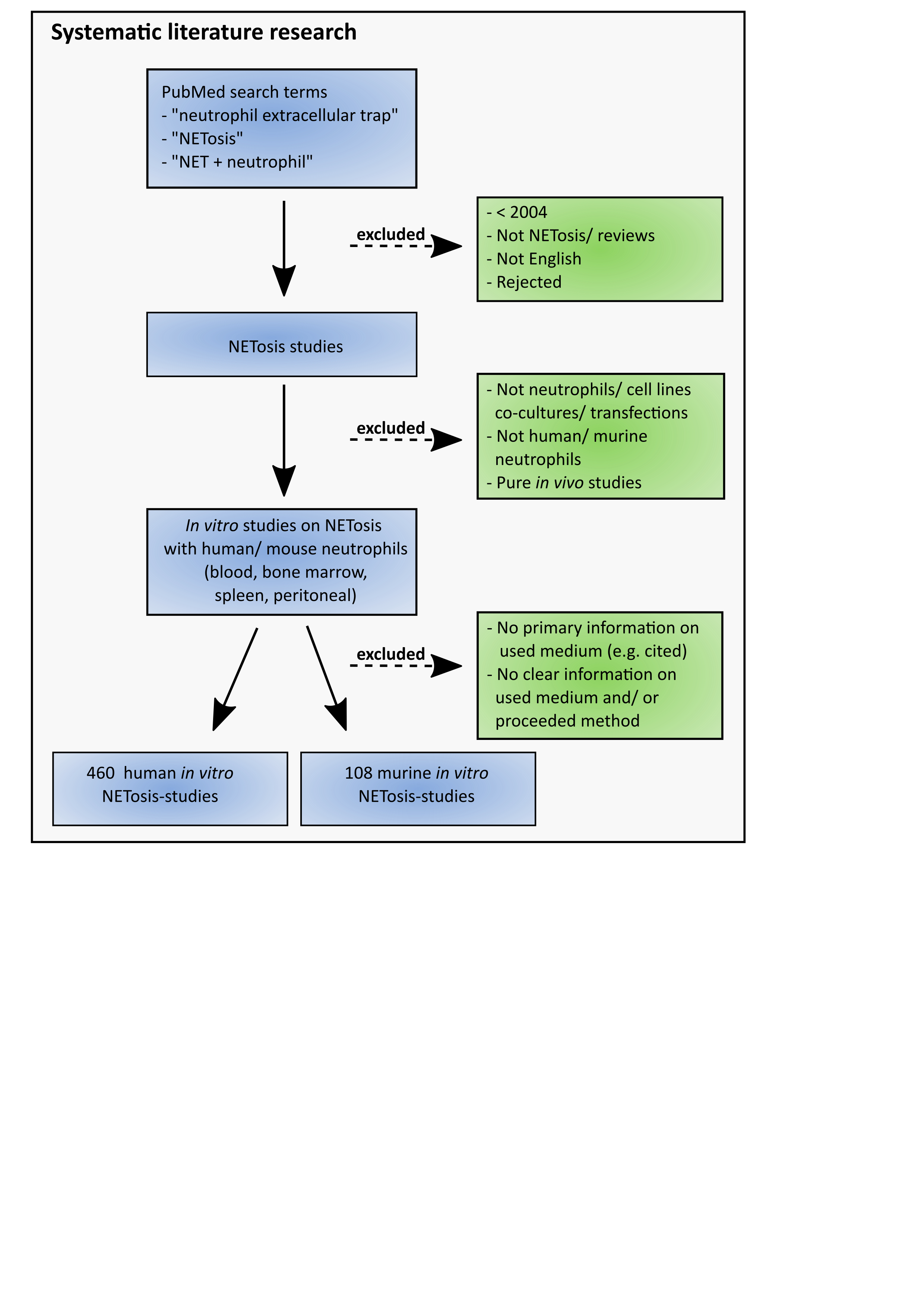
*

**Supplementary Figure 1: Literature research**.

**Supplementary Figure 2: Neutrophil elastase (NE) is released together with extracellular DNA after stimulation.** DNA-bound NE was measured after stimulation of human neutrophils by PMA (100 nM, 3h), CaI (4 µM, 2h) or LPS (100 µg/ml, 3h) in RPMI/HEPES with and without HSA. Neutrophils release DNA-bound NE in response to all stimuli. In media containing 0.5% HSA the release is clearly decreased after LPS and CaI stimulation, but appears stable in response to PMA. Error bars = mean ± SD. N = 2.


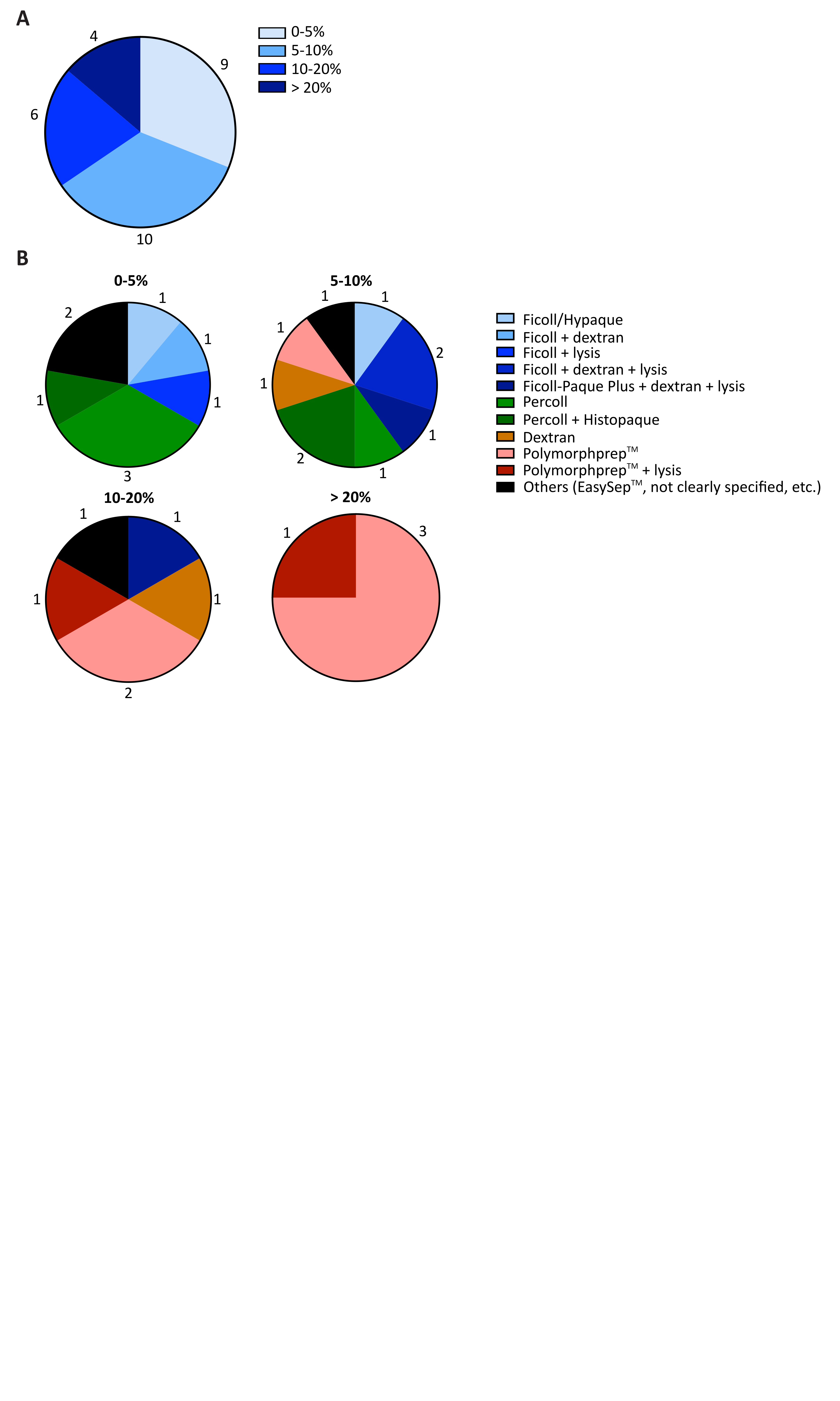


**Supplementary Figure 3: Spontaneous NETosis in literature. (A)** Percent of spontaneous NET formation in publications using serum- and albumin-free culture conditions. NETosis without further stimulation of isolated human neutrophils range between 0% and more than 20% NETotic cells. N = 29 publications with clear displayed percentage of NETs (out of all 255 publication without serum-supplements). **(B)** Spontaneous NET formation depending on neutrophil isolation technique. In literature neutrophils isolated by Polymorphprep^TM^ show a higher tendency for spontaneous NET formation than neutrophils isolated by density-gradient techniques.

**Supplementary Figure 4: HSA does not influence calcium concentrations.** The concentration of calcium in PBS does not significantly change in presence or absence of 0.5% HSA. Statistic: two-tailed t test. ns = not significant. N = 3. Error = SD.

**Supplementary Table 1: Culture media for stimulation of NETosis in human neutrophils**

| Supplement | **Concentration** | **Medium** | **Activator** |
| --- | --- | --- | --- |
| None | Not applicable | RPMI +/- sup. | PMA (10 nM [1], 12 nM [2], 16 nM [3], 20 nM [4-24], 24 nM [25], 25 nM [26-58] (TNFα -primed [45, 59]), 0.1 – 25 nM [60], 30 nM [61, 62], 32 nM [63], 40 nM [64-67], 20-40 nM [68], 50 nM [1, 45, 69-81], 100 nM [1, 82-109], 0-100 nM [110, 111], 162 nM [112, 113], 253 nM [114], 500 nM [81, 115, 116], 600 nM [117], 800 nM [118], 16 µM [119], 50 µM [120], 10 mM [121]) [1, 85, 122-125] |
|  |  |  | Calcium ionophore A23187 (2.5 µM [81, 84, 123], 4 µM [42, 43, 53, 100, 126], 5 µM [117], 25 µM [13, 115]); Ionomycin (1.3 µM [3], 5 µm [42, 43, 53, 126], 8 µM [61]) |
|  |  |  | LPS (10 ng/ml [60], 100 ng/ml [47, 54, 55, 70, 127, 128]+/- TNFα [45], 250 ng/ml [61], 0-10^4^ ng/ml [124], 1 μg/ml [5, 10, 13, 60, 129, 130], 2.5 µg/ml [3], 3 µg/ml [39], 5 µg/ml [53, 81], 0-25 µg/ml [52], 25 µg/ml [42, 53], 100 µg/ml [17], 100 nM [131]) [125] |
|  |  |  | Bacteria (*Staphylococcus aureus* [6, 26, 43, 53, 74, 113, 121, 124, 125, 132, 133] (+/- LL-37 [48]), *Neisseria gonorrhoeae* [9], *Pseudomonas aeruginosa* [23, 52, 53, 67, 125, 134, 135], *Streptococcus suis* [29]*, Streptococcus pneumonia* [98], *Streptococcus sanguinis* [136], *Streptococcus mutans* (+combinations of IgG /platelet/P-selectin [101]),  *Escherichia coli* [52], *Yersinia enterocolitica* [31], *Yersinia pseudotuberculosis* [137], *Helicobacter pylori* [68], *Haemophilus influenza* [138], *Aggregatibacter actinomycetemcomitans* [74, 112, 133]*,* *Fusobacterium nucleatum* [74, 133]*, Actinomyces viscosus* [133], *Vibrio cholerae* [96], *Staphylococcus epidermidis* biofilms [139], Periodontal bacteria [77], isolated bacterial cultured from plaques/ mixed bacterial population from plaque [131]) |
|  |  |  | Virus (Respiratory Syncytial Virus (RSV) [70], Dengue virus [113]) |
|  |  |  | Fungi (*Paraoccidioides brasiliensis* [39], *Candida albicans* [39, 56, 83, 104, 140], *Aspergillus* *fumigatus* [50], *Cryptococcus gattii/+ mutants* [141], *Candida glabrata* [141]*, Aspergillus* species [142]) |
|  |  |  | Other pathogens/parasites (*Naegleria fowleri* trophozoites [15], *Brugia malayi* microfilariae [38], *Leishmania amazonensis* promastigotes [82, 102, 109, 127], *Cryptosporidium parvum* sporozoites [143]) |
|  |  |  | Cytokines/ Chemokines (IL-8 [47, 54, 55, 83] +/- TNFα [45], IL17 +/- TNFα [130], IL-18 [10], TNF [61], TNFα [85, 144], platelet activating factor (PAF) [67, 68, 115, 145]) |
|  |  |  | Other stimuli [1-5, 11, 14, 17, 24, 25, 27, 30, 34, 40, 41, 45, 47, 48, 54-56, 59, 61, 63, 66-70, 74, 76-78, 80, 81, 85, 90, 91, 93, 95, 98, 99, 102, 104, 107, 111, 112, 114, 115, 119, 120, 122-124, 128, 130, 143, 144, 146-158] |
|  |  | HBSS +/- sup. | PMA (1.5 nM [159, 160], 10 nM [161], 20 nM [134, 162-164], 25 nM [30, 43, 165-172], 40.5 nM [173], 50 nM [174, 175], 100 nM [104, 176-180], 1-100 nM [181], 162 nM [182] [183], 1.6 µM [184], 1-100 nM [185], 10-1000 nM [186], (2 nM - 20 μM) [187], 1.6 mM [139]) [135, 188] |
|  |  |  | LPS (100 ng/mL [184], 1 µg/ml [159]) |
|  |  |  | Calcium ionophore (1 µM [189], 4 μM [43]) [188]; Ionomycin (5 μM [43]) |
|  |  |  | Bacteria (*Mycobacterium tuberculosis* [190], *Burkholderia pseudomallei* [162], *Staphylococcus aureus* [162], *Staphylococcus epidermidis* biofilms [139], *Streptococcus pyogenes* [172], *Pseudomonas* *aeruginosa* [191, 192], *Escherichia coli* [137], *Porphyromonas gingivalis* [193], mid-log bacterial cultures [194], *Bacillus anthracis* [171]) |
|  |  |  | Fungi (*Candida albicans* [104]) |
|  |  |  | Other pathogens/Parasites (*Trypanosoma cruzi* and soluble antigen [169]) |
|  |  |  | Cytokines/ Chemokines (IL8 [184]) |
|  |  |  | Other stimuli [3, 30, 139, 159, 160, 163, 166, 170, 172, 173, 178, 179, 182, 193, 195-201] |
|  |  | Other media and buffer | PMA (0.32 nM [202], 5 nM [203, 204], 20 nM [16, 205-208] (+/- TGF-β-pretreated [14]), 25 nM [37, 209], 40 nM [210], 50 nM [211], 81 nM [212, 213], 100 nM [162, 208, 214-220], 50-100 nM [221], 150 nM [222], 162 nM [202, 223], 600 nM [224], 649 nM [225, 226], 1 µM [227], 1.6µM [210]) [228] |
|  |  |  | Calcium ionophore (5 µM [229], 1 µM [230], 25 µM [231]) |
|  |  |  | LPS (0.1 ng/ml [232], 100 ng/ml [207, 232-235], 0.1-100 ng/mL [228, 236], 6 or 8 pg/neutrophil (TNFα/ IL-6/ IFNα-primed) [218]) |
|  |  |  | Virus (Influenza A virus (Phil82) (+/-LL-37-preincubation [225, 226]), dengue virus [207] |
|  |  |  | Bacteria (*Staphylococcus aureus* [207, 208, 228, 230, 237-239], *Escherichia coli* [228, 237], *Pseudomonas aeruginosa* [227, 230], *Mycoplasma pneumoniae* [222],  *Mycobacterium tuberculosis* [209]) |
|  |  |  | Fungi (*Candida albicans* (+/- fibronectin-precoating) [240, 241], *Aspergillus fumigatus* or *nidulans* [242]) |
|  |  |  | Cytokines/ Chemokines (PAF [228], IL-8 [223, 243, 244]) |
|  |  |  | Other stimuli [14, 204, 206, 212, 215, 220, 223, 228, 235, 240, 241, 243-255] |
| Fetal calf serum (FCS) | 0.05% | HBSS +/- sup. | PMA (2-250 nM [256]) |
|  |  |  | Calcium ionophore (0.2-25 µM [256]) |
|  | 0.5% | RPMI +/- sup. | PMA (10 nM [257, 258], 20 nM [259, 260], 25 nM [261, 262], 30 nM [263], 10–50 nM [264], 50 nM [265], 100 nM [266]) |
|  |  |  | Cytokines/ Chemokines (PAF [265], IL-8 [257, 265], TNFα [257, 258, 265], IL-1β [257, 267]) |
|  |  |  | Other stimuli [257, 259-261, 263, 264, 266, 268] |
|  |  | HBSS +/- sup. | PMA (2-250 nM [269]) |
|  |  |  | Calcium ionophore (5 µM [270]) |
|  | 1% | RPMI +/- sup. | PMA (20 nM [271], 25 nM [272, 273], 100 nM [274], 600 nM [275]) |
|  |  |  | LPS (0.7–100 μg/ ml [271], 2 -200 µg/mL [276]) [277] |
|  |  |  | Fungi (*Candida albicans* [274] |
|  |  |  | Cytokines/ Chemokines (TNFα [277]) |
|  |  |  | Other stimuli [272, 277] |
|  | 2% | RPMI +/- sup. | PMA (4 nM [278], 8 nM [279], 10 nM [280, 281], 20 nM [15, 280, 282-288], 25 nM [289], 32 nM [290], 40 nM [291], 50 nM [78], 100 nM [292-294], 120 nM [295], 600 nM [296]) |
|  |  |  | Ionomycin (0.626–5 µg/ml [285], 5 µM [288]) |
|  |  |  | LPS (100 ng/ml [278, 279], 300 ng/ml [287]) |
|  |  |  | Bacteria (*Staphylococcus aureus* [285, 289, 291], *Escherichia coli* [285]*, Pseudomonas aeruginosa* [285], *Wolbachia* (soluble, from supernatants of infected mosquito cells, conjugated fluorescent beads [296]) |
|  |  |  | Fungi (*Aspergillus fumigatus* [278], *C. albicans* [297] (biofilm and planktonic cells [294])) |
|  |  |  | Virus (RSV (+/- PMA) [295]) |
|  |  |  | Other pathogens/parasites (*Naegleria fowleri* [15]) |
|  |  |  | Cytokines/ Chemokines (IL-8 [278, 287, 298, 299], IL-17 [287], PAF [287, 288]) |
|  |  |  | Other stimuli [78, 281, 286, 291, 300] |
|  |  | Other media and buffer | PMA (20 nM [301], 25 nM [302], 100 nM [303]) |
|  |  |  | Bacteria (*Pseudomonas aeruginosa* [302], *Porphyromonas gingivalis* [303]) |
|  |  |  | Other stimuli [301] |
|  | 3% | RPMI +/- sup. | PMA (500 nM [304, 305]) |
|  | 4% | RPMI +/- sup. | PMA (50 nM [149], 100 nM [306]) |
|  |  |  | Other stimuli [306] |
|  | 5% | RPMI +/- sup. | PMA (10 nM [180, 307], 20 nM [308], 50 nM [309], 0-100 nM [310], 16.2 – 1620 nM [311], 1.62 µM (+/- retinoic acid) [312]) |
|  |  |  | LPS (1 µg/ml [311]) |
|  |  |  | Fungi (*Arthroderma benhamiae* [313]) |
|  |  |  | Other stimuli [4, 307, 309, 312] |
|  | 7.5% | Other media and buffer | PMA (100 nM [314]) |
|  |  |  | Other stimuli [314] |
|  | 10 % | RPMI +/- sup. | PMA (16 nM [315, 316], 10 nM [317], 20 nM [318], 32 nM [315], 50 nM [318, 319], 100 nM [104, 320, 321], 200 nM [136, 322], 600 nM [323]) |
|  |  |  | Ionomycin (5 μM [324]) [325] |
|  |  |  | LPS (200 ng/ml [319], LPS + IL-8 [325]) |
|  |  |  | Bacteria (*Streptococcus mutans* [320], *Streptococcus sanguinis* [136]) |
|  |  |  | Other pathogens/parasites (*Entamoeba histolytica* trophozoites [326]) |
|  |  |  | Cytokines/Chemokines (C5a (IFNα-, IFNγ- or GM-CSF-primed) [327], IL-6 [323], IL-9 [328]) |
|  |  |  | Other stimuli [136, 324, 329-331] |
|  |  | HBSS +/- sup. | PMA (100 nM [104, 332]) |
|  |  | Other media and buffer | PMA (81 nM [237], 40 nM (TNFα -primed [195]), 405 nM [237]) |
|  |  |  | LPS (10, 100, 1000, 5000 ng/ml [237], 0.2 μg/ml [333]) |
|  |  |  | Ionomycin (3 and 5 µg/ml [237]) |
|  |  |  | Other stimuli [195, 237, 333] |
| Heat inactivated FCS (hiFCS) | 0.1% | RPMI +/- sup. | PMA (100 nM[334]) |
|  |  |  | LPS (1 µg/ml [334]) |
|  |  |  | Virus (Hantaan virus (HTNV) and HTNV-infected supernatant (Vero E6 cells) [334], Vaccinia virus (VV) [334], Adenovirus [334]) |
|  |  |  | Other stimuli [334] |
|  | 0.5% | RPMI +/- sup. | PMA (50 nM (TNFα-primed) [335]) |
|  |  |  | LPS (100 ng/ml (TNFα-primed) [335]) |
|  |  |  | Other stimuli [335] |
|  | 1% | RPMI +/- sup. | PMA (20 nM [336]) |
|  |  |  | Bacteria (*Escherichia coli* [337]) |
|  |  |  | Other stimuli [337-339] |
|  | 2% | RPMI +/- sup. | PMA (40 nM [124, 150], 25 nM [35, 340-342] (normal/high glucose) [343], 50 nM [344], 100 nM [345-347] (normal/high glucose or mannitol) [343]) |
|  |  |  | LPS (1 µg/ml [124], 2 µg/ml (normal/high glucose or mannitol) [343]) |
|  |  |  | Ionomycin (normal/high glucose or mannitol [348]) |
|  |  |  | Bacteria (*Staphylococcus aureus* [124] (+ PMA [341])) |
|  |  |  | Fungi (*Candida albicans* biofilms [345], *candida glabrata* (biofilms and planktonic) [347]) |
|  |  |  | Cytokins/Chemokins (TNFα (normal/high glucose) [343], IL-6 (normal/high glucose) [343], IL-8 [346]) |
|  |  |  | Other stimuli [124, 150, 196, 343, 346] |
|  | 4% | RPMI +/- sup. | PMA (25 nM [349], 100 nM [350]) |
|  |  |  | LPS (100 ng/ml [351]) |
|  |  |  | Cytokines/chemokines (IL-8 [351]) |
|  | 5% | RPMI +/- sup. | PMA (25 nM [35], 81 nM [352], 100 nM [353], 3.2 µM [352]) |
|  |  |  | Bacteria (*Neisseria gonorrhoeae* [352]) |
|  |  |  | Fungi (*Candida albicans* [353], *Aspergillus fumigatus* [354]) |
|  | 10% | RPMI +/- sup. | PMA (5 nM [355], 20 nM [356], 25 nM [35], 32 nM[357], 40 nM [325] 65 nM [357], 162 nM [358, 359], 50 nM [355]) |
|  |  |  | LPS (100 ng/ml TNFα-or IL-8-primed [325]) |
|  |  |  | Ionomycin (1 µM [325]) |
|  |  |  | Fungi (*Paracoccidioides brasiliensis* [358, 359]) |
|  |  |  | Cytokins/Chemokins (IL-8 [325]) |
|  |  |  | Other stimuli [325, 360] |
|  |  | Other media and buffer | PMA (20 nM [361], 100 nM [361]) |
|  |  |  | Bacteria (*Pseudomona aeruginosa* [361]*, Bordetella parapertussis* [361]) |
| Human plasma (HP) | 3% | RPMI +/- sup. | Fungi (*Candida albicans* [274]) |
|  |  | HBSS +/- sup. | Bacteria (*Mycobacterium bovis* [362]) |
|  |  |  | Fungi (C*andida albicans* [362]) |
|  | 10% | PPMI +/- sup. | Cytokines/Chemokines (IL-8 [363]) |
|  |  | RPMI or HBSS +/- sup. | Fungi (*Candida. albicans* [104]) |
|  |  | Other media and buffer | LPS (5 µg/ml [247]) |
|  | 20% | RPMI +/- sup. | PMA (100 nM [98]) |
|  |  |  | Bacteria (*Streptococcus pneumonia* [98]) |
|  |  |  | Other stimuli [98] |
|  | 100% | - | PMA (100 nM [98]) |
|  |  |  | Bacteria (*Streptococcus pneumonia* [98]) |
|  |  |  | Other stimuli [98, 254, 364] |
| hiHP | 2% | RPMI +/- sup. | PMA (25 nM [365-367], 600 nM [368]) |
|  |  |  | Bacteria (group A *Streptococcus* (GAS) [365, 367, 369], *Lactococcus lactis* [367, 369]*, Pseudomonas aeruginosa* [366]) |
|  |  |  | Other pathogens/parasites (*Schistosoma japonicum* eggs, *soluble egg antigen or excretory/secretory products of* S. japonicum *eggs* [368]) |
|  |  |  | Other Stimuli [367] |
|  | 5% | RPMI +/- sup. | PMA (25 nM [37]) |
| Human Serum (HS) | 0.2% | HBSS +/- sup. | Other stimuli [370] |
|  | 1% | HBSS +/- sup. | PMA (100 nM [192, 371-374]) |
|  |  |  | Bacteria (*Pseudomonas aeruginosa* [192, 371, 372], *Escherichia coli* [375]) |
|  |  |  | Other stimuli [373, 374] |
|  | 2% | RPMI +/- sup. | PMA (20 nM [376], 25 nM [377], 65 nM [158], 200 nM [378], 600 nM [379]) [147, 380] |
|  |  |  | Ionophore (25 µM [376]) |
|  |  |  | Bacteria (*Escherichia coli* (+/- PMA) [378], *Acinetobacter baumannii* [381] (+/- PMA) [378], *Pseudomonas aeruginosa* [381]) |
|  |  |  | Cytokines/Chemokines (IL-1β [152], TNFα [379]) |
|  |  |  | Other stimuli [147, 148, 151, 152, 158, 376, 377, 380] |
|  |  | Other media and buffer | PMA (25 nM [382]) |
|  |  |  | Bacteria (*Yersinia enterocolitica/ Y.pestis* [382]) |
|  | 5% | RPMI +/- sup. | Other stimuli [383, 384] |
|  | 6% | RPMI +/- sup. | Bacteria (*E. coli* +/- TNFα/ G-CS/ IL-1β [385]) |
|  |  |  | Other stimuli [385] |
|  | 10% | RPMI +/- sup. | PMA (25 nM [229]) |
|  |  |  | LPS (50 ng/mL (+/- gallic acid) [386]) |
|  |  |  | Calcium ionophore (5 µM [229]) |
|  |  |  | Bacteria (*Mycobacterium bovis* bacillus Calmette–Guérin Pasteur [229], *Listeria monocytogenes* [387], *Klebsiella pneumoniae* [388]) |
|  |  |  | Other stimuli [386, 387] |
|  |  | HBSS +/- sup. | Fungi (*Candida albicans* [332]) |
|  | 25% | Other media and buffer | PMA [389] |
|  |  |  | Bacteria (*Klebsiella pneumoniae* [389]) |
|  | 100% | - | PMA (100 nM) [390] |
|  |  |  | Other pathogens/parasites (*Strongyloides stercoralis* infective larvae [390]) |
| hiHS | 0.1% | HBSS +/- sup. | Other stimuli [391] |
|  | 0.5% | HBSS +/- sup. | Other stimuli [392] |
|  |  | Other media and buffer | LPS (100 ng/ml [393-395]) |
|  |  |  | Ionophore [394] |
|  |  |  | Bacteria (*Staphylococcus aureus* [396]) |
|  |  |  | Other stimuli [393, 394] |
|  | 2% | RPMI +/- sup. | PMA (25 nM [397], 25 nM [398-400], 32 nM [401, 402], 10-50 nM [403]) [404] |
|  |  |  | LPS (2 µg/ml [401]) |
|  |  |  | Ionomycin (1–5 µM [403]) |
|  |  |  | Bacteria (GAS [397], Streptococcus pneumoniae [404] (PMA-pretreated) [399, 400]) |
|  |  |  | Fungi (*C. albicans* [33]) |
|  |  |  | Other pathogens/parasites (*Leishmania donovani/ major* [405]) |
|  |  |  | Cytokines/Chemokines (IL-8 [403], TNFα [398], IL-6 [401]) |
|  |  |  | Other stimuli [398, 401, 405] |
|  |  | HBSS +/- sup. | Other stimuli [60] |
|  |  | Other media and buffer | Cytokines/Chemokines [406] |
|  | 100% | - | PMA (100 nM [390]) |
|  |  |  | Other pathogens/parasites (*Strongyloides stercoralis* infective larvae [390]) |
| Human serum albumin (HSA) | 0.05% | RPMI +/- sup. | PMA (50 nM [407], 100 nM [191]) |
|  |  |  | Calcium ionophore (5 µM [407]) |
|  |  |  | Bacteria (GBS [407], *Staphylococcus aureus* [407]) |
|  |  |  | Fungi (*C. albicans* [407]) |
|  |  |  | Other stimuli [191, 407] |
|  | 0.1% | RPMI +/- sup. | Other stimuli [191] |
|  |  | HBSS +/- sup. | PMA (100 nM [197]) |
|  |  |  | Other stimuli [197] |
|  |  | Other media and buffer | PMA (50 µM [407]) |
|  |  |  | Calcium ionophore (5 µM [407]) |
|  |  |  | Bacteria (GBS [407]) |
|  |  |  | Fungi (*C. albicans* [407]) |
|  |  |  | Other stimuli [407] |
|  | 0.2% | RPMI +/- sup. | PMA (20 nM [408, 409], 100 nM [410]) |
|  |  |  | Bacteria (*Staphylococcus aureus* [408, 409], *Escherichia coli* [409]) |
|  |  |  | Fungi (*Candida albicans* [410]) |
|  |  |  | Other stimuli [408, 409] |
|  |  | HBSS +/- sup. | PMA (50 nM [411]) |
|  | 0.5% | RPMI +/- sup. | PMA (20 nM [412-414], 25 nM [415], 50 nM [416]) |
|  |  |  | Bacteria (*Pseudomonas aeruginosa* [416], [417]) |
|  |  |  | Cytokines/ Chemokines (IL-1β/TNFα/G-CSF [416]) |
|  |  |  | Other stimuli [414-416] |
|  |  | HBSS +/- sup. | PMA (60 nM [418]) |
|  |  |  | Other pathogens/parasites (*toxoplasma gondii* [418]) |
|  |  | Other media and buffer | PMA (162 nM [419]) |
|  | 1% | RPMI +/- sup. | PMA (10 nM [420], 5 nM - 50 nM [421]) |
|  |  |  | Bacteria (*Neisseria meningitidis* [421]) |
|  |  |  | Other stimuli [421] |
|  | 2% | RPMI +/- sup. | PMA (10-20 nM [396], 25 nM [194, 209, 422, 423], 100 nM [194, 424-426], 200 nM [322]) |
|  |  |  | LPS (1 μg/ml + LPS- binding protein [396]) |
|  |  |  | Bacteria (mid-log bacterial cultures [194], GBS [423], *Mycobacterium tuberculosis* [209]) |
|  |  |  | Fungi (*C. albicans* [33]) |
|  |  |  | Other pathogens/parasites (*Entamoeba histolytica* trophozoites [422]) |
|  |  |  | Cytokines/Chemokines (IL-8 [396]) |
|  |  |  | Other stimuli [396, 422] |
|  |  | HBSS +/- sup. | PMA (50 nM [427, 428], 100 nM [427]) |
|  |  |  | Other stimuli [428] |
|  |  | Other media and buffer | PMA (50 nM [429]) |
|  |  |  | Ionophore (5 µM [429]) |
|  |  |  | Other stimuli [429] |
|  | unknown | X-Vivo^TM^ 15 | PMA (20 nM – 81 nM (+/- human Ig coated well plates [287]), 20 nM [288], 25 nM [430-433], 600 nM [431]) |
|  |  |  | LPS (0.3 µg/mL (GM-CSF-primed) [430], 100 ng/ml (GM-CSF-primed) [432]) |
|  |  |  | Bacteria (*Escherichia coli* [430]) |
|  |  |  | Cytokines/Chemokines (C5a (GM-CSF-primed) [430, 432, 433]) |
|  |  |  | Other stimuli [431] |
| hiHSA | 2% | Other media and buffer | PMA (12.5–25 nM [434]) |
|  |  |  | Other stimuli [434] |
| Bovine serum albumin (BSA) | 0.1% | Other media and buffer | PMA (10 nM [435]) |
|  | 0.2% | RPMI +/- sup. | PMA (81 nM [436]) |
|  |  |  | Other stimuli [436] |
|  | 0.25% | HBSS +/- sup. | PMA (100 nM [437]) |
|  | 0.3% | Other media and buffer | PMA (50 nM [438]) |
|  | 0.5% | RPMI +/- sup. | PMA (10 nM [439], 25 nM [440], 27 nM [441], 50 nM [442], 100 nM [443], 250 nM [443]) |
|  |  |  | Bacteria (*Streptococcus gordonii* [442]*, Peptoanaerobacter stomatis* [442]*, Filifactor alocis* (+/- TNFα-primed) [442]) |
|  |  |  | Fungi (*Aspergillus fumigatus* [439]) |
|  |  |  | Other stimuli [440, 441] |
|  | 1% | RPMI +/- sup. | PMA (20 nM [444], 600 nM [117]) |
|  |  |  | Calcium ionophore (5 µM [117]) |
|  |  |  | Bacteria (GBS strains or hemolytic GBS pigment [444], *Escherichia coli* [445]) |
|  |  |  | Other stimuli [445] |
|  |  | HBSS +/- sup. | PMA (10 nM [446, 447] (PAF-pretreated) [448]) |
|  |  |  | Other stimuli [3, 446, 448] |
|  |  | Other media and buffer | Bacteria (*Escherichia coli* [445]) |
|  |  |  | Other stimuli [445] |
|  | 2% | RPMI +/- sup. | PMA (16 nM [449], 20 nM [450, 451], 50 nM [452-454], 81 nM [455]) |
|  |  |  | LPS (1 µg/ml [449]) |
|  |  |  | Bacteria (*Leptospira interrogans and biflexa* [455], *Staphylococcus aureus* [454]) |
|  |  |  | Cytokines/ Chemokines [452] |
|  |  | Other media and buffer | PMA (100 nM [219, 456]) |
| Albumin | 0.2% | RPMI +/- sup. | PMA (20 nM [457]) |
|  |  |  | Other stimuli [457] |
|  | 0.5% | Other media and buffer | PMA [458] |
|  |  |  | Fungi (*Aspergillus fumigatus* [458]) |
|  | 2% | RPMI +/- sup. | PMA (5-20 nM [459]) |
|  |  |  | Other stimuli [459] |
|  | 5% | RPMI +/- sup. | PMA (5-20 nM [459]) |
|  |  |  | Other stimuli [459] |
| Low serum | -- | RPMI +/- sup. | PMA (64 nM [460]) |
|  |  |  | Other stimuli [460] |

**Supplementary Table 2: Culture media for stimulation of NETosis in murine neutrophils**

| **Serum/**  **serum albumin** | **Concentration** | **Medium** | **Activator** |
| --- | --- | --- | --- |
| **None** | Not applicable | RPMI +/- supp. | PMA (20 nM [1, 2], 25 nM [3, 4], 50 nM [5-10], 100 nM [4, 11-17], 200 nM [18], 2.5 µM [13], 16.2 µM [19], 100 mM [20]) [21-23] |
|  |  |  | LPS (0.2 µg/ml [24], 0.5 µg/ml [25], 1 µg/ml [9], 10 µg/ml [10, 26] (TNFα-primed [27]), 2.5-25 µg/ml [28], 25 µg/ml [29]) [22] |
|  |  |  | Ionomycin (4 µM [4, 12, 15, 16, 20, 26, 30, 31], 1 µg/ml [19]); Ionophore (5 µM [9]) |
|  |  |  | Bacteria (*Streptococcus pneumoniae* +/- BALF after infection with PR8 virus [32], *Escherichia coli* +/- PMA [9], *Haemophilus influenzae* [33]) |
|  |  |  | Fungi (*Aspergillus fumigatus* [34]) |
|  |  |  | Parasites (*Plasmodium berghei* [35]) |
|  |  |  | Cytokines/ chemokines (MIP-2 [10], PAF [10, 20]) |
|  |  |  | Other stimuli [1, 3, 9-11, 13, 17, 19-22, 32, 36, 37] |
|  |  | HBSS +/- supp. | PMA (20 nM [38], 32 nM [39], 50 nM [40], 100 nM [41-43] (TNF-α primed [44]), 160 nM [45]) |
|  |  |  | LPS (0.1 µg/ml [42], 1 µg/mL [46], 10 µg/ml [28]) |
|  |  |  | Ionomycin (1 µg/mL [45]) |
|  |  |  | Bacteria (*Klebsiella pneumoniae* [47], *Streptococcus aureus* [41], commensal/ probiotic/ enterohemorrhagic *Escherichia coli* [41], TSB/ LB/ MRS broth fecal bacteria [48, 49]*, Pseudomonas aeruginosa* [50]) |
|  |  |  | Cytokines/ chemokines (PAF [51]) |
|  |  |  | Other stimuli [39, 41, 42, 44, 48, 49, 52] |
|  |  | Other media and buffer | PMA (20 nM [53, 54], 25 nM [55, 56], 50 nM [57], 100 nM [14, 58], 1 µM [59]) |
|  |  |  | LPS (100 ng/ml [55], 1 µg/ml [56, 60], 500 µg/ml [61]) |
|  |  |  | Calcium ionophore (4 µM [59]) |
|  |  |  | Bacteria (*Escherichia coli*, *Shigella flexneri* or GAS (IL-8-primed) [56]) |
|  |  |  | Cytokines/ chemokines (C5a (GM-CSF-primed) [55], IL8 [58]) |
|  |  |  | Other stimuli [53, 56-59, 61-63] |
| **FCS** | 0.5% | RPMI +/- supp. | PMA (10 nM [64]) |
|  |  |  | LPS (100 ng/ml [64]) |
|  |  |  | Other stimuli [64] |
|  |  | HBSS +/- supp. | PMA (500 nM [65]) |
|  | 1% | RPMI +/- supp. | PMA (100 nM [66]) |
|  |  |  | LPS (10 µg/ml [67]) |
|  |  |  | Other stimuli [66, 68, 69] |
|  | 2% | RPMI +/- supp. | PMA (100 nM (TNFα-primed [27], GM-CSF-primed [70]), 600 nM [71], 160 µM [72]) |
|  |  |  | LPS (10 µg/ml (GM-CSF-primed) [70]) |
|  |  |  | Bacteria (*Streptococcus aureus* (GM-CSF-primed) [70]*, Escherichia coli* (GM-CSF-primed) [70]*,* GBS [72], Wolbachia containing supernatant [71]) |
|  |  |  | Other stimuli [71] |
|  |  | Other media and buffer | PMA (20 nM [73], 50 nM [74], 20-80 nM [75], 100 nM [76]) [77] |
|  |  |  | Parasites (*Plasmodium berghei* lysate [74], *Pseudomonas aeruginosa* (+/- PMA) [76]) |
|  |  |  | Cytokines/ chemokines (IFNγ + C5a [73]) |
|  |  |  | Other stimuli [74] |
|  | 5% | RPMI +/- supp. | PMA (100 nM [78]) [79] |
|  |  |  | LPS (100 µg/mL [78]) |
|  |  |  | Fungi (*Candida albicans* [80]) |
|  |  |  | Other stimuli [78, 79] |
|  | 10% | RPMI +/- supp. | PMA (25 nM [81], 0-100 nM [82]) |
|  |  |  | Bacteria (*Streptococcus agalactiae* [83], *Escherichia coli* [84], *Staphylococcus aureus* [84]) |
|  |  | HBSS +/- supp. | PMA (100 nM [85]) |
|  |  | Other media | PMA (40 nM (TNFα –primed) [86]) |
|  |  |  | Other stimuli [86] |
|  | unknown | Other media and buffer | Other stimuli [87] |
| **hiFCS** | 0.1% | RPMI +/- supp. | PMA (3-2000 nM [88]) |
|  |  |  | Virus (Hantaan virus [88]) |
|  |  |  | Other stimuli [88] |
|  | 0.5% | RPMI +/- supp. | PMA [89] |
|  |  |  | Other stimuli [89] |
|  | 2% | RPMI +/- supp. | PMA (200 nM [90]) |
|  |  |  | Bacteria (*Streptococcus suis* (planktonic/ bacteria from biofilm matrix/ biofilms) +/- PMA [90]) |
|  | 10% | Other media and buffer | PMA (324 nM [91]) |
| **BSA** | 0.1% | RPMI +/- supp. | LPS (100 ng/ml [92]) |
|  | 0.5% | RPMI +/- supp. | PMA (324 nM [93]) |
|  |  |  | LPS (100 ng/ml (TNFα-priming) [93]) |
|  |  |  | Other Stimuli [93] |
|  | 1% | RPMI +/- supp. | PMA (162 nM (GM-CSF-primed) [70]) |
|  |  |  | LPS (10 µg/ml (GM-CSF-primed) [70]) |
|  |  | HBSS +/- supp. | LPS (100 ng/ml [94]) |
|  | 2% | RPMI +/- supp. | PMA (16 nM [60], 100 nM [95-98]) [99] |
|  |  |  | Other stimuli [95, 96, 98, 99] |
|  |  | Other media and buffer | Bacteria (*Staphylococcus aureus* (methicillin-resistant) [5]) |
| **HSA** | unknown | X-VIVO 15 | PMA (25 nM [100]) |
|  |  |  | LPS (100 ng/ml (GM-CSF-primed) [100]) |
|  |  |  | Cytokines/ chemokines (C5a (GM-CSF-primed [100]) |
|  |  |  | Other stimuli [100] |
| **Mouse serum (MS)** | 1% | RPMI +/- supp. | PMA (100 nM [101-103]) |
|  |  |  | Ionomycin (4 µM [103]) |
|  |  |  | Bacteria (*Staphylococcus aureus* (methicillin-resistant) [103] |
|  | 10% | RPMI +/- supp. | Bacteria (*Listeria monocytogenes* [104]) |
|  | 100% | - | PMA (100 nM [105]) |
|  |  |  | Parasites (*Strongyloides stercoralis* infective larvae [105]) |
| **DNase (-/-) MS** | 2% | RPMI +/- supp. | PMA (100 nM [106]) |
|  |  |  | Fungi (*Candida albicans* (heat killed) [106]) |
|  |  | HBSS +/- supp. | PMA (100 nM [107]) |
|  |  |  | Fungi (*Candida albicans* [107]) |
|  |  |  | Bacteria (*Listeria monocytogenes* [107]) |
| **hiMS** | 100% | - | PMA (100 nM [105]) |
|  |  |  | Parasites (*Strongyloides stercoralis* infective larvae [105]) |
| **Bovine growth serum (BGS)** | 2% | Other media and buffer | PMA (600 µM [108]) |
|  |  |  | Parasites (*Toxoplasma gondii* [108]) |

**References for human NETosis-experiments**

1. Rodriguez-Espinosa, O., et al., *Metabolic requirements for neutrophil extracellular traps formation.* Immunology, 2015. **145**(2): p. 213-24.

2. Hair, P.S., et al., *Inhibition of Immune Complex Complement Activation and Neutrophil Extracellular Trap Formation by Peptide Inhibitor of Complement C1.* Front Immunol, 2018. **9**: p. 558.

3. Maueroder, C., et al., *Menage-a-Trois: The Ratio of Bicarbonate to CO2 and the pH Regulate the Capacity of Neutrophils to Form NETs.* Front Immunol, 2016. **7**: p. 583.

4. Aleman, O.R., et al., *Differential Use of Human Neutrophil Fcgamma Receptors for Inducing Neutrophil Extracellular Trap Formation.* J Immunol Res, 2016. **2016**: p. 2908034.

5. Carmona-Rivera, C., et al., *Neutrophil extracellular traps induce endothelial dysfunction in systemic lupus erythematosus through the activation of matrix metalloproteinase-2.* Ann Rheum Dis, 2015. **74**(7): p. 1417-24.

6. Csomos, K., et al., *Protein cross-linking by chlorinated polyamines and transglutamylation stabilizes neutrophil extracellular traps.* Cell Death Dis, 2016. **7**(8): p. e2332.

7. Juneau, R.A., et al., *Peroxiredoxin-glutaredoxin and catalase promote resistance of nontypeable Haemophilus influenzae 86-028NP to oxidants and survival within neutrophil extracellular traps.* Infect Immun, 2015. **83**(1): p. 239-46.

8. Bhongir, R.K., et al., *DNA-fragmentation is a source of bactericidal activity against Pseudomonas aeruginosa.* Biochem J, 2017. **474**(3): p. 411-425.

9. Juneau, R.A., et al., *A thermonuclease of Neisseria gonorrhoeae enhances bacterial escape from killing by neutrophil extracellular traps.* J Infect Dis, 2015. **212**(2): p. 316-24.

10. Kahlenberg, J.M., et al., *Neutrophil extracellular trap-associated protein activation of the NLRP3 inflammasome is enhanced in lupus macrophages.* J Immunol, 2013. **190**(3): p. 1217-26.

11. Skrzeczynska-Moncznik, J., et al., *Secretory leukocyte proteinase inhibitor-competent DNA deposits are potent stimulators of plasmacytoid dendritic cells: implication for psoriasis.* J Immunol, 2012. **189**(4): p. 1611-7.

12. Urban, C.F., et al., *Neutrophil extracellular traps contain calprotectin, a cytosolic protein complex involved in host defense against Candida albicans.* PLoS Pathog, 2009. **5**(10): p. e1000639.

13. Zhao, W., D.K. Fogg, and M.J. Kaplan, *A novel image-based quantitative method for the characterization of NETosis.* J Immunol Methods, 2015. **423**: p. 104-10.

14. Aleman, O.R., et al., *Transforming Growth Factor-beta-Activated Kinase 1 Is Required for Human FcgammaRIIIb-Induced Neutrophil Extracellular Trap Formation.* Front Immunol, 2016. **7**: p. 277.

15. Contis-Montes de Oca, A., et al., *Neutrophils extracellular traps damage Naegleria fowleri trophozoites opsonized with human IgG.* Parasite Immunol, 2016. **38**(8): p. 481-95.

16. Smith, C.K., et al., *Neutrophil extracellular trap-derived enzymes oxidize high-density lipoprotein: an additional proatherogenic mechanism in systemic lupus erythematosus.* Arthritis Rheumatol, 2014. **66**(9): p. 2532-44.

17. Yalavarthi, S., et al., *Release of neutrophil extracellular traps by neutrophils stimulated with antiphospholipid antibodies: a newly identified mechanism of thrombosis in the antiphospholipid syndrome.* Arthritis Rheumatol, 2015. **67**(11): p. 2990-3003.

18. Leffler, J., et al., *A subset of patients with systemic lupus erythematosus fails to degrade DNA from multiple clinically relevant sources.* Arthritis Res Ther, 2015. **17**: p. 205.

19. Leffler, J., et al., *Decreased Neutrophil Extracellular Trap Degradation in Shiga Toxin-Associated Haemolytic Uraemic Syndrome.* J Innate Immun, 2017. **9**(1): p. 12-21.

20. Leffler, J., et al., *Degradation of neutrophil extracellular traps co-varies with disease activity in patients with systemic lupus erythematosus.* Arthritis Res Ther, 2013. **15**(4): p. R84.

21. Leffler, J., et al., *Degradation of neutrophil extracellular traps is decreased in patients with antiphospholipid syndrome.* Clin Exp Rheumatol, 2014. **32**(1): p. 66-70.

22. Leffler, J., et al., *Neutrophil extracellular traps that are not degraded in systemic lupus erythematosus activate complement exacerbating the disease.* J Immunol, 2012. **188**(7): p. 3522-31.

23. Yuen, J., et al., *NETosing Neutrophils Activate Complement Both on Their Own NETs and Bacteria via Alternative and Non-alternative Pathways.* Front Immunol, 2016. **7**: p. 137.

24. Lood, C. and G.C. Hughes, *Neutrophil extracellular traps as a potential source of autoantigen in cocaine-associated autoimmunity.* Rheumatology (Oxford), 2017. **56**(4): p. 638-643.

25. Menten-Dedoyart, C., et al., *Neutrophil extracellular traps entrap and kill Borrelia burgdorferi sensu stricto spirochetes and are not affected by Ixodes ricinus tick saliva.* J Immunol, 2012. **189**(11): p. 5393-401.

26. Branitzki-Heinemann, K., et al., *Formation of Neutrophil Extracellular Traps under Low Oxygen Level.* Front Immunol, 2016. **7**: p. 518.

27. Desai, J., et al., *PMA and crystal-induced neutrophil extracellular trap formation involves RIPK1-RIPK3-MLKL signaling.* Eur J Immunol, 2016. **46**(1): p. 223-9.

28. de Buhr, N., et al., *Streptococcus suis DNase SsnA contributes to degradation of neutrophil extracellular traps (NETs) and evasion of NET-mediated antimicrobial activity.* Microbiology, 2014. **160**(Pt 2): p. 385-95.

29. de Buhr, N., et al., *Identification of a novel DNase of Streptococcus suis (EndAsuis) important for neutrophil extracellular trap degradation during exponential growth.* Microbiology, 2015. **161**(Pt 4): p. 838-50.

30. Brogden, G., et al., *Methods to Study Lipid Alterations in Neutrophils and the Subsequent Formation of Neutrophil Extracellular Traps.* J Vis Exp, 2017(121).

31. Mollerherm, H., et al., *Yersinia enterocolitica-mediated degradation of neutrophil extracellular traps (NETs).* FEMS Microbiol Lett, 2015. **362**(23): p. fnv192.

32. Palmer, L.J., et al., *Extracellular deoxyribonuclease production by periodontal bacteria.* J Periodontal Res, 2012. **47**(4): p. 439-45.

33. Urban, C.F., et al., *Neutrophil extracellular traps capture and kill Candida albicans yeast and hyphal forms.* Cell Microbiol, 2006. **8**(4): p. 668-76.

34. Vollger, L., et al., *Iron-chelating agent desferrioxamine stimulates formation of neutrophil extracellular traps (NETs) in human blood-derived neutrophils.* Biosci Rep, 2016. **36**(3).

35. von Kockritz-Blickwede, M., O.A. Chow, and V. Nizet, *Fetal calf serum contains heat-stable nucleases that degrade neutrophil extracellular traps.* Blood, 2009. **114**(25): p. 5245-6.

36. Xu, D., et al., *Heparan Sulfate Modulates Neutrophil and Endothelial Function in Antibacterial Innate Immunity.* Infect Immun, 2015. **83**(9): p. 3648-56.

37. Cogen, A.L., et al., *Staphylococcus epidermidis antimicrobial delta-toxin (phenol-soluble modulin-gamma) cooperates with host antimicrobial peptides to kill group A Streptococcus.* PLoS One, 2010. **5**(1): p. e8557.

38. McCoy, C.J., et al., *Human Leukocytes Kill Brugia malayi Microfilariae Independently of DNA-Based Extracellular Trap Release.* PLoS Negl Trop Dis, 2017. **11**(1): p. e0005279.

39. Mejia, S.P., et al., *Human neutrophils produce extracellular traps against Paracoccidioides brasiliensis.* Microbiology, 2015. **161**(Pt 5): p. 1008-17.

40. Nakazawa, D., et al., *Histones and Neutrophil Extracellular Traps Enhance Tubular Necrosis and Remote Organ Injury in Ischemic AKI.* J Am Soc Nephrol, 2017. **28**(6): p. 1753-1768.

41. Neumann, A., et al., *Lipid alterations in human blood-derived neutrophils lead to formation of neutrophil extracellular traps.* Eur J Cell Biol, 2014. **93**(8-9): p. 347-54.

42. Khan, M.A. and N. Palaniyar, *Transcriptional firing helps to drive NETosis.* Sci Rep, 2017. **7**: p. 41749.

43. Douda, D.N., et al., *SK3 channel and mitochondrial ROS mediate NADPH oxidase-independent NETosis induced by calcium influx.* Proc Natl Acad Sci U S A, 2015. **112**(9): p. 2817-22.

44. Farrera, C. and B. Fadeel, *Macrophage clearance of neutrophil extracellular traps is a silent process.* J Immunol, 2013. **191**(5): p. 2647-56.

45. Hazeldine, J., et al., *Impaired neutrophil extracellular trap formation: a novel defect in the innate immune system of aged individuals.* Aging Cell, 2014. **13**(4): p. 690-8.

46. Farrera, C., et al., *Extracellular entrapment and degradation of single-walled carbon nanotubes.* Nanoscale, 2014. **6**(12): p. 6974-83.

47. Greenwood, H., et al., *Simvastatin to modify neutrophil function in older patients with septic pneumonia (SNOOPI): study protocol for a randomised placebo-controlled trial.* Trials, 2014. **15**: p. 332.

48. Neumann, A., et al., *The antimicrobial peptide LL-37 facilitates the formation of neutrophil extracellular traps.* Biochem J, 2014. **464**(1): p. 3-11.

49. Schilcher, K., et al., *Increased neutrophil extracellular trap-mediated Staphylococcus aureus clearance through inhibition of nuclease activity by clindamycin and immunoglobulin.* J Infect Dis, 2014. **210**(3): p. 473-82.

50. McCormick, A., et al., *NETs formed by human neutrophils inhibit growth of the pathogenic mold Aspergillus fumigatus.* Microbes Infect, 2010. **12**(12-13): p. 928-36.

51. Neumann, A., et al., *Novel role of the antimicrobial peptide LL-37 in the protection of neutrophil extracellular traps against degradation by bacterial nucleases.* J Innate Immun, 2014. **6**(6): p. 860-8.

52. Khan, M.A., et al., *JNK Activation Turns on LPS- and Gram-Negative Bacteria-Induced NADPH Oxidase-Dependent Suicidal NETosis.* Sci Rep, 2017. **7**(1): p. 3409.

53. Nadesalingam, A., et al., *Hypertonic Saline Suppresses NADPH Oxidase-Dependent Neutrophil Extracellular Trap Formation and Promotes Apoptosis.* Front Immunol, 2018. **9**: p. 359.

54. Richardson, J.J.R., et al., *Neutrophil Extracellular Trap Production in Patients with Colorectal Cancer In Vitro.* Int J Inflam, 2017. **2017**: p. 4915062.

55. Richardson, J.J.R., et al., *Characterization of systemic neutrophil function in patients undergoing colorectal cancer resection.* J Surg Res, 2017. **220**: p. 410-418.e1.

56. Zawrotniak, M., et al., *Aspartic Proteases and Major Cell Wall Components in Candida albicans Trigger the Release of Neutrophil Extracellular Traps.* Front Cell Infect Microbiol, 2017. **7**: p. 414.

57. Flores, R., et al., *The Selective Estrogen Receptor Modulator Raloxifene Inhibits Neutrophil Extracellular Trap Formation.* Front Immunol, 2016. **7**: p. 566.

58. Palmer, L.J., et al., *Hypochlorous acid regulates neutrophil extracellular trap release in humans.* Clin Exp Immunol, 2012. **167**(2): p. 261-8.

59. Thomas, G.M., et al., *Extracellular DNA traps are associated with the pathogenesis of TRALI in humans and mice.* Blood, 2012. **119**(26): p. 6335-43.

60. Ohbuchi, A., et al., *Quantitative analysis of hemin-induced neutrophil extracellular trap formation and effects of hydrogen peroxide on this phenomenon.* Biochem Biophys Rep, 2017. **11**: p. 147-153.

61. Liu, C.L., et al., *Specific post-translational histone modifications of neutrophil extracellular traps as immunogens and potential targets of lupus autoantibodies.* Arthritis Res Ther, 2012. **14**(1): p. R25.

62. Chen, X., et al., *ATAC-see reveals the accessible genome by transposase-mediated imaging and sequencing.* Nat Methods, 2016. **13**(12): p. 1013-1020.

63. Nani, S., et al., *Src family kinases and Syk are required for neutrophil extracellular trap formation in response to beta-glucan particles.* J Innate Immun, 2015. **7**(1): p. 59-73.

64. Bianchi, M., et al., *Restoration of NET formation by gene therapy in CGD controls aspergillosis.* Blood, 2009. **114**(13): p. 2619-22.

65. Bianchi, M., et al., *Restoration of anti-Aspergillus defense by neutrophil extracellular traps in human chronic granulomatous disease after gene therapy is calprotectin-dependent.* J Allergy Clin Immunol, 2011. **127**(5): p. 1243-52.e7.

66. Malachowa, N., et al., *Staphylococcus aureus leukotoxin GH promotes formation of neutrophil extracellular traps.* J Immunol, 2013. **191**(12): p. 6022-9.

67. Martinez, N.E., et al., *Tetrahydroisoquinolines: New Inhibitors of Neutrophil Extracellular Trap (NET) Formation.* Chembiochem, 2017. **18**(10): p. 888-893.

68. Hakkim, A., et al., *Activation of the Raf-MEK-ERK pathway is required for neutrophil extracellular trap formation.* Nat Chem Biol, 2011. **7**(2): p. 75-7.

69. Bjornsdottir, H., et al., *Phenol-Soluble Modulin alpha Peptide Toxins from Aggressive Staphylococcus aureus Induce Rapid Formation of Neutrophil Extracellular Traps through a Reactive Oxygen Species-Independent Pathway.* Front Immunol, 2017. **8**: p. 257.

70. Funchal, G.A., et al., *Respiratory syncytial virus fusion protein promotes TLR-4-dependent neutrophil extracellular trap formation by human neutrophils.* PLoS One, 2015. **10**(4): p. e0124082.

71. Agraz-Cibrian, J.M., et al., *Alterations in neutrophil extracellular traps is associated with the degree of decompensation of liver cirrhosis.* J Infect Dev Ctries, 2016. **10**(5): p. 512-7.

72. Gogol, M., et al., *Inactivation of alpha1-proteinase inhibitor by Candida albicans aspartic proteases favors the epithelial and endothelial cell colonization in the presence of neutrophil extracellular traps.* Acta Biochim Pol, 2016. **63**(1): p. 167-175.

73. Mohammed, B.M., et al., *Vitamin C: a novel regulator of neutrophil extracellular trap formation.* Nutrients, 2013. **5**(8): p. 3131-51.

74. Roberts, H., et al., *Characterization of neutrophil function in Papillon-Lefevre syndrome.* J Leukoc Biol, 2016. **100**(2): p. 433-44.

75. Saffarzadeh, M., et al., *Neutrophil extracellular traps directly induce epithelial and endothelial cell death: a predominant role of histones.* PLoS One, 2012. **7**(2): p. e32366.

76. White, P., et al., *Peripheral blood neutrophil extracellular trap production and degradation in chronic periodontitis.* J Clin Periodontol, 2016. **43**(12): p. 1041-1049.

77. White, P.C., et al., *Characterization, Quantification, and Visualization of Neutrophil Extracellular Traps.* Methods Mol Biol, 2017. **1537**: p. 481-497.

78. Bjornsdottir, H., et al., *Neutrophil NET formation is regulated from the inside by myeloperoxidase-processed reactive oxygen species.* Free Radic Biol Med, 2015. **89**: p. 1024-35.

79. O'Donoghue, A.J., et al., *Global substrate profiling of proteases in human neutrophil extracellular traps reveals consensus motif predominantly contributed by elastase.* PLoS One, 2013. **8**(9): p. e75141.

80. Chicca, I.J., et al., *Development and Application of High-Content Biological Screening for Modulators of NET Production.* Front Immunol, 2018. **9**: p. 337.

81. Irizarry-Caro, J.A., et al., *Brief Report: Drugs Implicated in Systemic Autoimmunity Modulate Neutrophil Extracellular Trap Formation.* Arthritis Rheumatol, 2018. **70**(3): p. 468-474.

82. DeSouza-Vieira, T., et al., *Neutrophil extracellular traps release induced by Leishmania: role of PI3Kgamma, ERK, PI3Ksigma, PKC, and [Ca2+].* J Leukoc Biol, 2016. **100**(4): p. 801-810.

83. Hosseinzadeh, A., P.K. Messer, and C.F. Urban, *Stable Redox-Cycling Nitroxide Tempol Inhibits NET Formation.* Front Immunol, 2012. **3**: p. 391.

84. Carmona-Rivera, C. and M.J. Kaplan, *Induction and Quantification of NETosis.* Curr Protoc Immunol, 2016. **115**: p. 14.41.1-14.41.14.

85. Lee, J., et al., *Nicotine drives neutrophil extracellular traps formation and accelerates collagen-induced arthritis.* Rheumatology (Oxford), 2017. **56**(4): p. 644-653.

86. Manzenreiter, R., et al., *Ultrastructural characterization of cystic fibrosis sputum using atomic force and scanning electron microscopy.* J Cyst Fibros, 2012. **11**(2): p. 84-92.

87. Pieterse, E., et al., *Neutrophil Extracellular Traps Drive Endothelial-to-Mesenchymal Transition.* Arterioscler Thromb Vasc Biol, 2017.

88. Pratesi, F., et al., *Antibodies from patients with rheumatoid arthritis target citrullinated histone 4 contained in neutrophils extracellular traps.* Ann Rheum Dis, 2014. **73**(7): p. 1414-22.

89. Thomas, K.A., et al., *TREM-like transcript 2 is stored in human neutrophil primary granules and is up-regulated in response to inflammatory mediators.* J Leukoc Biol, 2016. **100**(1): p. 177-84.

90. Huang, Y.M., et al., *Promotion of hypercoagulability in antineutrophil cytoplasmic antibody-associated vasculitis by C5a-induced tissue factor-expressing microparticles and neutrophil extracellular traps.* Arthritis Rheumatol, 2015. **67**(10): p. 2780-90.

91. Jansen, M.P., et al., *Release of extracellular DNA influences renal ischemia reperfusion injury by platelet activation and formation of neutrophil extracellular traps.* Kidney Int, 2017. **91**(2): p. 352-364.

92. Guimaraes-Costa, A.B., et al., *3'-nucleotidase/nuclease activity allows Leishmania parasites to escape killing by neutrophil extracellular traps.* Infect Immun, 2014. **82**(4): p. 1732-40.

93. Domingo-Gonzalez, R., et al., *Inhibition of Neutrophil Extracellular Trap Formation after Stem Cell Transplant by Prostaglandin E2.* Am J Respir Crit Care Med, 2016. **193**(2): p. 186-97.

94. Achouiti, A., et al., *Myeloid-related protein-14 contributes to protective immunity in gram-negative pneumonia derived sepsis.* PLoS Pathog, 2012. **8**(10): p. e1002987.

95. Bystrzycka, W., et al., *The effect of clindamycin and amoxicillin on neutrophil extracellular trap (NET) release.* Cent Eur J Immunol, 2016. **41**(1): p. 1-5.

96. Seper, A., et al., *Vibrio cholerae evades neutrophil extracellular traps by the activity of two extracellular nucleases.* PLoS Pathog, 2013. **9**(9): p. e1003614.

97. Niemiec, M.J., et al., *Trace element landscape of resting and activated human neutrophils on the sub-micrometer level.* Metallomics, 2015. **7**(6): p. 996-1010.

98. Schorn, C., et al., *Monosodium urate crystals induce extracellular DNA traps in neutrophils, eosinophils, and basophils but not in mononuclear cells.* Front Immunol, 2012. **3**: p. 277.

99. Tang, S., et al., *Neutrophil extracellular trap formation is associated with autophagy-related signalling in ANCA-associated vasculitis.* Clin Exp Immunol, 2015. **180**(3): p. 408-18.

100. Manda-Handzlik, A., et al., *Antibiotics Modulate the Ability of Neutrophils to Release Neutrophil Extracellular Traps.* Adv Exp Med Biol, 2017. **944**: p. 47-52.

101. Jung, C.J., et al., *Endocarditis pathogen promotes vegetation formation by inducing intravascular neutrophil extracellular traps through activated platelets.* Circulation, 2015. **131**(6): p. 571-81.

102. Guimaraes-Costa, A.B., et al., *Leishmania amazonensis promastigotes induce and are killed by neutrophil extracellular traps.* Proc Natl Acad Sci U S A, 2009. **106**(16): p. 6748-53.

103. Thomas, M.P., et al., *Leukocyte protease binding to nucleic acids promotes nuclear localization and cleavage of nucleic acid binding proteins.* J Immunol, 2014. **192**(11): p. 5390-7.

104. Metzler, K.D., et al., *Myeloperoxidase is required for neutrophil extracellular trap formation: implications for innate immunity.* Blood, 2011. **117**(3): p. 953-9.

105. Grasso, S., et al., *Interaction of factor VII activating protease (FSAP) with neutrophil extracellular traps (NETs).* Thromb Res, 2018. **161**: p. 36-42.

106. Niemiec, M.J., et al., *Dual transcriptome of the immediate neutrophil and Candida albicans interplay.* BMC Genomics, 2017. **18**(1): p. 696.

107. Sperling, C., et al., *Neutrophil extracellular trap formation upon exposure of hydrophobic materials to human whole blood causes thrombogenic reactions.* Biomater Sci, 2017. **5**(10): p. 1998-2008.

108. Bystrzycka, W., et al., *Azithromycin and Chloramphenicol Diminish Neutrophil Extracellular Traps (NETs) Release.* Int J Mol Sci, 2017. **18**(12).

109. Rochael, N.C., et al., *Classical ROS-dependent and early/rapid ROS-independent release of Neutrophil Extracellular Traps triggered by Leishmania parasites.* Sci Rep, 2015. **5**: p. 18302.

110. Golbach, L.A., et al., *Low-Frequency Electromagnetic Field Exposure Enhances Extracellular Trap Formation by Human Neutrophils through the NADPH Pathway.* J Innate Immun, 2015. **7**(5): p. 459-65.

111. Schneider, A.E., et al., *Complement factor H modulates the activation of human neutrophil granulocytes and the generation of neutrophil extracellular traps.* Mol Immunol, 2016. **72**: p. 37-48.

112. Hirschfeld, J., et al., *Effects of Aggregatibacter actinomycetemcomitans leukotoxin on neutrophil migration and extracellular trap formation.* J Oral Microbiol, 2016. **8**: p. 33070.

113. Moreno-Altamirano, M.M., et al., *Dengue Virus Serotype-2 Interferes with the Formation of Neutrophil Extracellular Traps.* Intervirology, 2015. **58**(4): p. 250-9.

114. Chow, O.A., et al., *Statins enhance formation of phagocyte extracellular traps.* Cell Host Microbe, 2010. **8**(5): p. 445-54.

115. Gupta, S., et al., *A High-Throughput Real-Time Imaging Technique To Quantify NETosis and Distinguish Mechanisms of Cell Death in Human Neutrophils.* J Immunol, 2018. **200**(2): p. 869-879.

116. Zou, Y., et al., *Neutrophil extracellular traps promote lipopolysaccharide-induced airway inflammation and mucus hypersecretion in mice.* Oncotarget, 2018. **9**(17): p. 13276-13286.

117. Noubouossie, D.F., et al., *In vitro activation of coagulation by human neutrophil DNA and histone proteins but not neutrophil extracellular traps.* Blood, 2017. **129**(8): p. 1021-1029.

118. Cools-Lartigue, J., et al., *Neutrophil extracellular traps sequester circulating tumor cells and promote metastasis.* J Clin Invest, 2013.

119. Biermann, M.H., et al., *Oxidative Burst-Dependent NETosis Is Implicated in the Resolution of Necrosis-Associated Sterile Inflammation.* Front Immunol, 2016. **7**: p. 557.

120. Liz, R., et al., *Silver nanoparticles rapidly induce atypical human neutrophil cell death by a process involving inflammatory caspases and reactive oxygen species and induce neutrophil extracellular traps release upon cell adhesion.* Int Immunopharmacol, 2015. **28**(1): p. 616-25.

121. Grassle, S., et al., *von Willebrand factor directly interacts with DNA from neutrophil extracellular traps.* Arterioscler Thromb Vasc Biol, 2014. **34**(7): p. 1382-9.

122. Berkes, E., et al., *Association of neutrophil extracellular traps with endometriosis-related chronic inflammation.* Eur J Obstet Gynecol Reprod Biol, 2014. **183**: p. 193-200.

123. Carmona-Rivera, C., et al., *A role for muscarinic receptors in neutrophil extracellular trap formation and levamisole-induced autoimmunity.* JCI Insight, 2017. **2**(3): p. e89780.

124. Van Avondt, K., et al., *Signal Inhibitory Receptor on Leukocytes-1 Limits the Formation of Neutrophil Extracellular Traps, but Preserves Intracellular Bacterial Killing.* J Immunol, 2016. **196**(9): p. 3686-94.

125. Khan, M.A., et al., *Regulating NETosis: Increasing pH Promotes NADPH Oxidase-Dependent NETosis.* Front Med (Lausanne), 2018. **5**: p. 19.

126. Naffah de Souza, C., et al., *Alkaline pH Promotes NADPH Oxidase-Independent Neutrophil Extracellular Trap Formation: A Matter of Mitochondrial Reactive Oxygen Species Generation and Citrullination and Cleavage of Histone.* Front Immunol, 2017. **8**: p. 1849.

127. Guimaraes-Costa, A.B., et al., *Neutrophil Extracellular Traps Reprogram IL-4/GM-CSF-Induced Monocyte Differentiation to Anti-inflammatory Macrophages.* Front Immunol, 2017. **8**: p. 523.

128. Mori, Y., et al., *alpha-Enolase of Streptococcus pneumoniae induces formation of neutrophil extracellular traps.* J Biol Chem, 2012. **287**(13): p. 10472-81.

129. Carmona-Rivera, C. and M.J. Kaplan, *Detection of SLE antigens in neutrophil extracellular traps (NETs).* Methods Mol Biol, 2014. **1134**: p. 151-61.

130. Khandpur, R., et al., *NETs are a source of citrullinated autoantigens and stimulate inflammatory responses in rheumatoid arthritis.* Sci Transl Med, 2013. **5**(178): p. 178ra40.

131. Hirschfeld, J., et al., *Neutrophil extracellular trap formation in supragingival biofilms.* Int J Med Microbiol, 2015. **305**(4-5): p. 453-63.

132. Lewis, H.D., et al., *Inhibition of PAD4 activity is sufficient to disrupt mouse and human NET formation.* Nat Chem Biol, 2015. **11**(3): p. 189-91.

133. Palmer, L.J., et al., *Influence of complement on neutrophil extracellular trap release induced by bacteria.* J Periodontal Res, 2016. **51**(1): p. 70-6.

134. Dwyer, M., et al., *Cystic fibrosis sputum DNA has NETosis characteristics and neutrophil extracellular trap release is regulated by macrophage migration-inhibitory factor.* J Innate Immun, 2014. **6**(6): p. 765-79.

135. Shan, Q., et al., *Distinct susceptibilities of corneal Pseudomonas aeruginosa clinical isolates to neutrophil extracellular trap-mediated immunity.* Infect Immun, 2014. **82**(10): p. 4135-43.

136. Sumioka, R., et al., *Streptococcus sanguinis induces neutrophil cell death by production of hydrogen peroxide.* PLoS One, 2017. **12**(2): p. e0172223.

137. Gillenius, E. and C.F. Urban, *The adhesive protein invasin of Yersinia pseudotuberculosis induces neutrophil extracellular traps via beta1 integrins.* Microbes Infect, 2015. **17**(5): p. 327-36.

138. Juneau, R.A., et al., *Nontypeable Haemophilus influenzae initiates formation of neutrophil extracellular traps.* Infect Immun, 2011. **79**(1): p. 431-8.

139. Dapunt, U., et al., *Activation of phagocytic cells by Staphylococcus epidermidis biofilms: effects of extracellular matrix proteins and the bacterial stress protein GroEL on netosis and MRP-14 release.* Pathog Dis, 2016. **74**(5).

140. Kenno, S., et al., *Autophagy and Reactive Oxygen Species Are Involved in Neutrophil Extracellular Traps Release Induced by C. albicans Morphotypes.* Front Microbiol, 2016. **7**: p. 879.

141. Springer, D.J., et al., *Extracellular fibrils of pathogenic yeast Cryptococcus gattii are important for ecological niche, murine virulence and human neutrophil interactions.* PLoS One, 2010. **5**(6): p. e10978.

142. Imbert, S., et al., *Calcineurin inhibitors impair neutrophil activity against Aspergillus fumigatus in allogeneic hematopoietic stem cell transplant recipients.* J Allergy Clin Immunol, 2016. **138**(3): p. 860-8.

143. Munoz-Caro, T., et al., *NADPH oxidase, MPO, NE, ERK1/2, p38 MAPK and Ca2+ influx are essential for Cryptosporidium parvum-induced NET formation.* Dev Comp Immunol, 2015. **52**(2): p. 245-54.

144. Yu, Y., et al., *Celastrol inhibits inflammatory stimuli-induced neutrophil extracellular trap formation.* Curr Mol Med, 2015. **15**(4): p. 401-10.

145. Fuchs, T.A., et al., *Extracellular DNA traps promote thrombosis.* Proc Natl Acad Sci U S A, 2010. **107**(36): p. 15880-5.

146. He, Z., et al., *Phosphotidylserine exposure and neutrophil extracellular traps enhance procoagulant activity in patients with inflammatory bowel disease.* Thromb Haemost, 2016. **115**(4): p. 738-51.

147. Apostolidou, E., et al., *Neutrophil extracellular traps regulate IL-1beta-mediated inflammation in familial Mediterranean fever.* Ann Rheum Dis, 2016. **75**(1): p. 269-77.

148. Skendros, P., et al., *Regulated in development and DNA damage responses 1 (REDD1) links stress with IL-1beta-mediated familial Mediterranean fever attack through autophagy-driven neutrophil extracellular traps.* J Allergy Clin Immunol, 2017.

149. Zhang, S., et al., *Enhanced formation and impaired degradation of neutrophil extracellular traps in dermatomyositis and polymyositis: a potential contributor to interstitial lung disease complications.* Clin Exp Immunol, 2014. **177**(1): p. 134-41.

150. Van Avondt, K., et al., *Ligation of signal inhibitory receptor on leukocytes-1 suppresses the release of neutrophil extracellular traps in systemic lupus erythematosus.* PLoS One, 2013. **8**(10): p. e78459.

151. Stakos, D.A., et al., *Expression of functional tissue factor by neutrophil extracellular traps in culprit artery of acute myocardial infarction.* Eur Heart J, 2015. **36**(22): p. 1405-14.

152. Mitroulis, I., et al., *Neutrophil extracellular trap formation is associated with IL-1beta and autophagy-related signaling in gout.* PLoS One, 2011. **6**(12): p. e29318.

153. Desai, J., et al., *Particles of different sizes and shapes induce neutrophil necroptosis followed by the release of neutrophil extracellular trap-like chromatin.* Sci Rep, 2017. **7**(1): p. 15003.

154. Lutaty, A., et al., *A 17-kDa Fragment of Lactoferrin Associates With the Termination of Inflammation and Peptides Within Promote Resolution.* Front Immunol, 2018. **9**: p. 644.

155. Zhu, L., et al., *Competence-independent activity of pneumococcal EndA [corrected] mediates degradation of extracellular dna and nets and is important for virulence.* PLoS One, 2013. **8**(7): p. e70363.

156. Douda, D.N., et al., *A lipid mediator hepoxilin A3 is a natural inducer of neutrophil extracellular traps in human neutrophils.* Mediators Inflamm, 2015. **2015**: p. 520871.

157. Rebernick, R., et al., *DNA Area and NETosis Analysis (DANA): a High-Throughput Method to Quantify Neutrophil Extracellular Traps in Fluorescent Microscope Images.* Biol Proced Online, 2018. **20**: p. 7.

158. Chrysanthopoulou, A., et al., *Neutrophil extracellular traps promote differentiation and function of fibroblasts.* J Pathol, 2014. **233**(3): p. 294-307.

159. Lipp, P., et al., *Less Neutrophil Extracellular Trap Formation in Term Newborns than in Adults.* Neonatology, 2017. **111**(2): p. 182-188.

160. Ruhnau, J., et al., *Stroke alters respiratory burst in neutrophils and monocytes.* Stroke, 2014. **45**(3): p. 794-800.

161. Bokaba, R.P., et al., *Cigarette smoke condensate attenuates phorbol ester-mediated neutrophil extracellular trap formation.* Afr Health Sci, 2017. **17**(3): p. 896-904.

162. de Jong, H.K., et al., *Neutrophil extracellular traps in the host defense against sepsis induced by Burkholderia pseudomallei (melioidosis).* Intensive Care Med Exp, 2014. **2**(1): p. 21.

163. Bekeschus, S., et al., *Neutrophil extracellular trap formation is elicited in response to cold physical plasma.* J Leukoc Biol, 2016. **100**(4): p. 791-799.

164. Arroyo, A.B., et al., *MiR-146a Regulates Neutrophil Extracellular Trap Formation That Predicts Adverse Cardiovascular Events in Patients With Atrial Fibrillation.* Arterioscler Thromb Vasc Biol, 2018. **38**(4): p. 892-902.

165. Iversen, M.B., et al., *Extracellular superoxide dismutase is present in secretory vesicles of human neutrophils and released upon stimulation.* Free Radic Biol Med, 2016. **97**: p. 478-88.

166. Corriden, R., et al., *Tamoxifen augments the innate immune function of neutrophils through modulation of intracellular ceramide.* Nat Commun, 2015. **6**: p. 8369.

167. Tillack, K., et al., *T lymphocyte priming by neutrophil extracellular traps links innate and adaptive immune responses.* J Immunol, 2012. **188**(7): p. 3150-9.

168. Tillack, K., et al., *Gender differences in circulating levels of neutrophil extracellular traps in serum of multiple sclerosis patients.* J Neuroimmunol, 2013. **261**(1-2): p. 108-19.

169. Sousa-Rocha, D., et al., *Trypanosoma cruzi and Its Soluble Antigens Induce NET Release by Stimulating Toll-Like Receptors.* PLoS One, 2015. **10**(10): p. e0139569.

170. Haripriyan, J., et al., *Clove Bud Oil Modulates Pathogenicity Phenotypes of the Opportunistic Human Pathogen Pseudomonas aeruginosa.* Sci Rep, 2018. **8**(1): p. 3437.

171. Liu, J.Z., et al., *Innate Immune Interactions between Bacillus anthracis and Host Neutrophils.* Front Cell Infect Microbiol, 2018. **8**: p. 2.

172. Neumann, A., et al., *Immunoregulation of Neutrophil Extracellular Trap Formation by Endothelial-Derived p33 (gC1q Receptor).* J Innate Immun, 2018. **10**(1): p. 30-43.

173. Chen, K., et al., *Endocytosis of soluble immune complexes leads to their clearance by FcgammaRIIIB but induces neutrophil extracellular traps via FcgammaRIIA in vivo.* Blood, 2012. **120**(22): p. 4421-31.

174. Ricci-Azevedo, R., et al., *Neutrophils Contribute to the Protection Conferred by ArtinM against Intracellular Pathogens: A Study on Leishmania major.* PLoS Negl Trop Dis, 2016. **10**(4): p. e0004609.

175. Costa, M.F., et al., *Eugenia aurata and Eugenia punicifolia HBK inhibit inflammatory response by reducing neutrophil adhesion, degranulation and NET release.* BMC Complement Altern Med, 2016. **16**(1): p. 403.

176. Syu, G.D., H.I. Chen, and C.J. Jen, *Acute severe exercise facilitates neutrophil extracellular trap formation in sedentary but not active subjects.* Med Sci Sports Exerc, 2013. **45**(2): p. 238-44.

177. Tonello, S., et al., *Low concentrations of neutrophil extracellular traps induce proliferation in human keratinocytes via NF-kB activation.* J Dermatol Sci, 2017.

178. Azevedo, E.P., et al., *A Metabolic Shift toward Pentose Phosphate Pathway Is Necessary for Amyloid Fibril- and Phorbol 12-Myristate 13-Acetate-induced Neutrophil Extracellular Trap (NET) Formation.* J Biol Chem, 2015. **290**(36): p. 22174-83.

179. Andrade, M.F., et al., *The 3-phenylcoumarin derivative 6,7-dihydroxy-3-[3',4'-methylenedioxyphenyl]-coumarin downmodulates the FcgammaR- and CR-mediated oxidative metabolism and elastase release in human neutrophils: Possible mechanisms underlying inhibition of the formation and release of neutrophil extracellular traps.* Free Radic Biol Med, 2018. **115**: p. 421-435.

180. Gray, R.D., et al., *Delayed neutrophil apoptosis enhances NET formation in cystic fibrosis.* Thorax, 2018. **73**(2): p. 134-144.

181. Dicker, A.J., et al., *Neutrophil extracellular traps are associated with disease severity and microbiota diversity in patients with chronic obstructive pulmonary disease.* J Allergy Clin Immunol, 2018. **141**(1): p. 117-127.

182. Arai, Y., et al., *Uric acid induces NADPH oxidase-independent neutrophil extracellular trap formation.* Biochem Biophys Res Commun, 2014. **443**(2): p. 556-61.

183. Maueroder, C., et al., *Capability of Neutrophils to Form NETs Is Not Directly Influenced by a CMA-Targeting Peptide.* Front Immunol, 2017. **8**: p. 16.

184. Alfaro, C., et al., *Tumor-Produced Interleukin-8 Attracts Human Myeloid-Derived Suppressor Cells and Elicits Extrusion of Neutrophil Extracellular Traps (NETs).* Clin Cancer Res, 2016. **22**(15): p. 3924-36.

185. Dicker, A.J., et al., *Neutrophil Extracellular Traps are associated with disease severity and microbiota diversity in Chronic Obstructive Pulmonary Disease.* J Allergy Clin Immunol, 2017.

186. Gray, R.D., et al., *Activation of conventional protein kinase C (PKC) is critical in the generation of human neutrophil extracellular traps.* J Inflamm (Lond), 2013. **10**(1): p. 12.

187. Jimenez-Valdes, R.J., et al., *Massive Parallel Analysis of Single Cells in an Integrated Microfluidic Platform.* Anal Chem, 2017. **89**(10): p. 5210-5220.

188. Neeli, I. and M. Radic, *Opposition between PKC isoforms regulates histone deimination and neutrophil extracellular chromatin release.* Front Immunol, 2013. **4**: p. 38.

189. Dwivedi, N., et al., *Deimination of linker histones links neutrophil extracellular trap release with autoantibodies in systemic autoimmunity.* Faseb j, 2014. **28**(7): p. 2840-51.

190. Arcos, J., et al., *Lung Mucosa Lining Fluid Modification of Mycobacterium tuberculosis to Reprogram Human Neutrophil Killing Mechanisms.* J Infect Dis, 2015. **212**(6): p. 948-58.

191. Rada, B., et al., *Pyocyanin-enhanced neutrophil extracellular trap formation requires the NADPH oxidase.* PLoS One, 2013. **8**(1): p. e54205.

192. Yoo, D.G., et al., *Release of cystic fibrosis airway inflammatory markers from Pseudomonas aeruginosa-stimulated human neutrophils involves NADPH oxidase-dependent extracellular DNA trap formation.* J Immunol, 2014. **192**(10): p. 4728-38.

193. Delbosc, S., et al., *Porphyromonas gingivalis participates in pathogenesis of human abdominal aortic aneurysm by neutrophil activation. Proof of concept in rats.* PLoS One, 2011. **6**(4): p. e18679.

194. Halverson, T.W., et al., *DNA is an antimicrobial component of neutrophil extracellular traps.* PLoS Pathog, 2015. **11**(1): p. e1004593.

195. Schreiber, A., et al., *Necroptosis controls NET generation and mediates complement activation, endothelial damage, and autoimmune vasculitis.* Proc Natl Acad Sci U S A, 2017. **114**(45): p. E9618-e9625.

196. Hollands, A., et al., *Natural Product Anacardic Acid from Cashew Nut Shells Stimulates Neutrophil Extracellular Trap Production and Bactericidal Activity.* J Biol Chem, 2016. **291**(27): p. 13964-73.

197. Pang, L., et al., *Pseudogout-associated inflammatory calcium pyrophosphate dihydrate microcrystals induce formation of neutrophil extracellular traps.* J Immunol, 2013. **190**(12): p. 6488-500.

198. Warnatsch, A., et al., *Inflammation. Neutrophil extracellular traps license macrophages for cytokine production in atherosclerosis.* Science, 2015. **349**(6245): p. 316-20.

199. J, G.N., et al., *Pneumolysin activates neutrophil extracellular trap formation.* Clin Exp Immunol, 2016. **184**(3): p. 358-67.

200. Leppkes, M., et al., *Externalized decondensed neutrophil chromatin occludes pancreatic ducts and drives pancreatitis.* Nat Commun, 2016. **7**: p. 10973.

201. Arumugam, S., et al., *Neutrophil extracellular traps in acrolein promoted hepatic ischemia reperfusion injury: Therapeutic potential of NOX2 and p38MAPK inhibitors.* J Cell Physiol, 2018. **233**(4): p. 3244-3261.

202. Kono, M., et al., *Iron-chelating agent, deferasirox, inhibits neutrophil activation and extracellular trap formation.* Clin Exp Pharmacol Physiol, 2016. **43**(10): p. 915-20.

203. Waisberg, M., et al., *Plasmodium falciparum infection induces expression of a mosquito salivary protein (Agaphelin) that targets neutrophil function and inhibits thrombosis without impairing hemostasis.* PLoS Pathog, 2014. **10**(9): p. e1004338.

204. Mizurini, D.M., et al., *Salivary Thromboxane A2-Binding Proteins from Triatomine Vectors of Chagas Disease Inhibit Platelet-Mediated Neutrophil Extracellular Traps (NETs) Formation and Arterial Thrombosis.* PLoS Negl Trop Dis, 2015. **9**(6): p. e0003869.

205. Pieterse, E., et al., *Acetylated histones contribute to the immunostimulatory potential of neutrophil extracellular traps in systemic lupus erythematosus.* Clin Exp Immunol, 2015. **179**(1): p. 68-74.

206. Itagaki, K., et al., *Mitochondrial DNA released by trauma induces neutrophil extracellular traps.* PLoS One, 2015. **10**(3): p. e0120549.

207. Yost, C.C., et al., *Neonatal NET-inhibitory factor and related peptides inhibit neutrophil extracellular trap formation.* J Clin Invest, 2016. **126**(10): p. 3783-3798.

208. Pilsczek, F.H., et al., *A novel mechanism of rapid nuclear neutrophil extracellular trap formation in response to Staphylococcus aureus.* J Immunol, 2010. **185**(12): p. 7413-25.

209. Braian, C., V. Hogea, and O. Stendahl, *Mycobacterium tuberculosis- induced neutrophil extracellular traps activate human macrophages.* J Innate Immun, 2013. **5**(6): p. 591-602.

210. Clark, S.R., et al., *Esterified eicosanoids are acutely generated by 5-lipoxygenase in primary human neutrophils and in human and murine infection.* Blood, 2011. **117**(6): p. 2033-43.

211. Bjornsdottir, H., et al., *Quantification of heterotypic granule fusion in human neutrophils by imaging flow cytometry.* Data Brief, 2016. **6**: p. 386-93.

212. Haase, H., et al., *Ethylmercury and Hg2+ induce the formation of neutrophil extracellular traps (NETs) by human neutrophil granulocytes.* Arch Toxicol, 2016. **90**(3): p. 543-50.

213. Haas, C.M., et al., *Proton-pump inhibitors elevate infection rate in cardiothoracic surgery patients by influencing PMN function in vitro and in vivo.* J Leukoc Biol, 2018. **103**(4): p. 777-788.

214. Pires, R.H., S.B. Felix, and M. Delcea, *The architecture of neutrophil extracellular traps investigated by atomic force microscopy.* Nanoscale, 2016. **8**(29): p. 14193-202.

215. Miyoshi, A., et al., *Circulating Neutrophil Extracellular Trap Levels in Well-Controlled Type 2 Diabetes and Pathway Involved in Their Formation Induced by High-Dose Glucose.* Pathobiology, 2016. **83**(5): p. 243-51.

216. Jimenez-Alcazar, M., et al., *Impaired DNase1-mediated degradation of neutrophil extracellular traps is associated with acute thrombotic microangiopathies.* J Thromb Haemost, 2015. **13**(5): p. 732-42.

217. Panda, R., et al., *Neutrophil Extracellular Traps Contain Selected Antigens of Anti-Neutrophil Cytoplasmic Antibodies.* Front Immunol, 2017. **8**: p. 439.

218. Pieterse, E., et al., *Neutrophils Discriminate between Lipopolysaccharides of Different Bacterial Sources and Selectively Release Neutrophil Extracellular Traps.* Front Immunol, 2016. **7**: p. 484.

219. Jimenez-Alcazar, M., et al., *Host DNases prevent vascular occlusion by neutrophil extracellular traps.* Science, 2017. **358**(6367): p. 1202-1206.

220. Rother, N., et al., *Acetylated Histones in Apoptotic Microparticles Drive the Formation of Neutrophil Extracellular Traps in Active Lupus Nephritis.* Front Immunol, 2017. **8**: p. 1136.

221. Varju, I., et al., *DNA, histones and neutrophil extracellular traps exert anti-fibrinolytic effects in a plasma environment.* Thromb Haemost, 2015. **113**(6): p. 1289-98.

222. Yamamoto, T., et al., *Mpn491, a secreted nuclease of Mycoplasma pneumoniae, plays a critical role in evading killing by neutrophil extracellular traps.* Cell Microbiol, 2017. **19**(3).

223. Manfredi, A.A., et al., *Low molecular weight heparins prevent the induction of autophagy of activated neutrophils and the formation of neutrophil extracellular traps.* Pharmacol Res, 2017.

224. van der Spek, A.H., et al., *The Thyroid Hormone Inactivating Enzyme Type 3 Deiodinase is Present in Bactericidal Granules and the Cytoplasm of Human Neutrophils.* Endocrinology, 2016. **157**(8): p. 3293-305.

225. Tripathi, S., et al., *LL-37 modulates human neutrophil responses to influenza A virus.* J Leukoc Biol, 2014. **96**(5): p. 931-8.

226. Tripathi, S., et al., *Identifying the Critical Domain of LL-37 Involved in Mediating Neutrophil Activation in the Presence of Influenza Virus: Functional and Structural Analysis.* PLoS One, 2015. **10**(8): p. e0133454.

227. Moussavi-Harami, S.F., et al., *Microfluidic device for simultaneous analysis of neutrophil extracellular traps and production of reactive oxygen species.* Integr Biol (Camb), 2016. **8**(2): p. 243-52.

228. Yost, C.C., et al., *Impaired neutrophil extracellular trap (NET) formation: a novel innate immune deficiency of human neonates.* Blood, 2009. **113**(25): p. 6419-27.

229. Stephan, A., et al., *LL37:DNA complexes provide antimicrobial activity against intracellular bacteria in human macrophages.* Immunology, 2016. **148**(4): p. 420-32.

230. Dubois, A.V., et al., *Influence of DNA on the activities and inhibition of neutrophil serine proteases in cystic fibrosis sputum.* Am J Respir Cell Mol Biol, 2012. **47**(1): p. 80-6.

231. Ammollo, C.T., et al., *Histones Differentially Modulate the Anticoagulant and Profibrinolytic Activities of Heparin, Heparin Derivatives, and Dabigatran.* J Pharmacol Exp Ther, 2016. **356**(2): p. 305-13.

232. Araujo, C.V., et al., *A PPARgamma AGONIST ENHANCES BACTERIAL CLEARANCE THROUGH NEUTROPHIL EXTRACELLULAR TRAP FORMATION AND IMPROVES SURVIVAL IN SEPSIS.* Shock, 2016. **45**(4): p. 393-403.

233. Glenn, J.W., et al., *Deficient Neutrophil Extracellular Trap Formation in Patients Undergoing Bone Marrow Transplantation.* Front Immunol, 2016. **7**: p. 250.

234. MacQueen, B.C., et al., *Elevated fecal calprotectin levels during necrotizing enterocolitis are associated with activated neutrophils extruding neutrophil extracellular traps.* J Perinatol, 2016. **36**(10): p. 862-9.

235. Kraemer, B.F., et al., *Novel anti-bacterial activities of beta-defensin 1 in human platelets: suppression of pathogen growth and signaling of neutrophil extracellular trap formation.* PLoS Pathog, 2011. **7**(11): p. e1002355.

236. McInturff, A.M., et al., *Mammalian target of rapamycin regulates neutrophil extracellular trap formation via induction of hypoxia-inducible factor 1 alpha.* Blood, 2012. **120**(15): p. 3118-25.

237. Hoppenbrouwers, T., et al., *In vitro induction of NETosis: Comprehensive live imaging comparison and systematic review.* PLoS One, 2017. **12**(5): p. e0176472.

238. Laabei, M., et al., *Evolutionary Trade-Offs Underlie the Multi-faceted Virulence of Staphylococcus aureus.* PLoS Biol, 2015. **13**(9): p. e1002229.

239. Hoppenbrouwers, T., et al., *Staphylococcal Protein A Is a Key Factor in Neutrophil Extracellular Traps Formation.* Front Immunol, 2018. **9**: p. 165.

240. Byrd, A.S., et al., *NETosis in Neonates: Evidence of a Reactive Oxygen Species-Independent Pathway in Response to Fungal Challenge.* J Infect Dis, 2016. **213**(4): p. 634-9.

241. Byrd, A.S., et al., *An extracellular matrix-based mechanism of rapid neutrophil extracellular trap formation in response to Candida albicans.* J Immunol, 2013. **190**(8): p. 4136-48.

242. Lee, M.J., et al., *The Fungal Exopolysaccharide Galactosaminogalactan Mediates Virulence by Enhancing Resistance to Neutrophil Extracellular Traps.* PLoS Pathog, 2015. **11**(10): p. e1005187.

243. Oehmcke, S., M. Morgelin, and H. Herwald, *Activation of the human contact system on neutrophil extracellular traps.* J Innate Immun, 2009. **1**(3): p. 225-30.

244. Manfredi, A.A., et al., *Instructive influences of phagocytic clearance of dying cells on neutrophil extracellular trap generation.* Clin Exp Immunol, 2015. **179**(1): p. 24-9.

245. Jovic, S., et al., *The neutrophil-recruiting chemokine GCP-2/CXCL6 is expressed in cystic fibrosis airways and retains its functional properties after binding to extracellular DNA.* Mucosal Immunol, 2016. **9**(1): p. 112-23.

246. Maugeri, N., et al., *Activated platelets present high mobility group box 1 to neutrophils, inducing autophagy and promoting the extrusion of neutrophil extracellular traps.* J Thromb Haemost, 2014. **12**(12): p. 2074-88.

247. Clark, S.R., et al., *Platelet TLR4 activates neutrophil extracellular traps to ensnare bacteria in septic blood.* Nat Med, 2007. **13**(4): p. 463-9.

248. von Bruhl, M.L., et al., *Monocytes, neutrophils, and platelets cooperate to initiate and propagate venous thrombosis in mice in vivo.* J Exp Med, 2012. **209**(4): p. 819-35.

249. Munoz, L.E., et al., *Nanoparticles size-dependently initiate self-limiting NETosis-driven inflammation.* Proc Natl Acad Sci U S A, 2016. **113**(40): p. E5856-e5865.

250. Xu, P.C., et al., *C-reactive protein enhances activation of coagulation system and inflammatory response through dissociating into monomeric form in antineutrophil cytoplasmic antibody-associated vasculitis.* BMC Immunol, 2015. **16**: p. 10.

251. Sandler, N., et al., *Mitochondrial DAMPs Are Released During Cardiopulmonary Bypass Surgery and Are Associated With Postoperative Atrial Fibrillation.* Heart Lung Circ, 2017.

252. Johnson, C.M., et al., *Integrin Cross-Talk Regulates the Human Neutrophil Response to Fungal beta-Glucan in the Context of the Extracellular Matrix: A Prominent Role for VLA3 in the Antifungal Response.* J Immunol, 2017. **198**(1): p. 318-334.

253. Park, J., et al., *Cancer cells induce metastasis-supporting neutrophil extracellular DNA traps.* Sci Transl Med, 2016. **8**(361): p. 361ra138.

254. Ciciliano, J.C., et al., *Probing blood cell mechanics of hematologic processes at the single micron level.* Lab Chip, 2017. **17**(22): p. 3804-3816.

255. Kono, M., et al., *Heme-related molecules induce rapid production of neutrophil extracellular traps.* Transfusion, 2014. **54**(11): p. 2811-9.

256. Barrientos, L., et al., *An improved strategy to recover large fragments of functional human neutrophil extracellular traps.* Front Immunol, 2013. **4**: p. 166.

257. Keshari, R.S., et al., *Cytokines induced neutrophil extracellular traps formation: implication for the inflammatory disease condition.* PLoS One, 2012. **7**(10): p. e48111.

258. Soderberg, D., et al., *Increased levels of neutrophil extracellular trap remnants in the circulation of patients with small vessel vasculitis, but an inverse correlation to anti-neutrophil cytoplasmic antibodies during remission.* Rheumatology (Oxford), 2015. **54**(11): p. 2085-94.

259. Keshari, R.S., et al., *Reactive oxygen species-induced activation of ERK and p38 MAPK mediates PMA-induced NETs release from human neutrophils.* J Cell Biochem, 2013. **114**(3): p. 532-40.

260. Keshari, R.S., et al., *Neutrophil extracellular traps contain mitochondrial as well as nuclear DNA and exhibit inflammatory potential.* Cytometry A, 2012. **81**(3): p. 238-47.

261. Clapp, P.W., et al., *Flavored e-cigarette liquids and cinnamaldehyde impair respiratory innate immune cell function.* Am J Physiol Lung Cell Mol Physiol, 2017. **313**(2): p. L278-l292.

262. Reidel, B., et al., *E-Cigarette Use Causes a Unique Innate Immune Response in the Lung, Involving Increased Neutrophilic Activation and Altered Mucin Secretion.* Am J Respir Crit Care Med, 2018. **197**(4): p. 492-501.

263. Awasthi, D., et al., *Oxidized LDL induced extracellular trap formation in human neutrophils via TLR-PKC-IRAK-MAPK and NADPH-oxidase activation.* Free Radic Biol Med, 2016. **93**: p. 190-203.

264. Patel, S., et al., *Nitric oxide donors release extracellular traps from human neutrophils by augmenting free radical generation.* Nitric Oxide, 2010. **22**(3): p. 226-34.

265. Marin Oyarzun, C.P., et al., *Neutrophil extracellular trap formation and circulating nucleosomes in patients with chronic myeloproliferative neoplasms.* Sci Rep, 2016. **6**: p. 38738.

266. Ma, Y.H., et al., *High-mobility group box 1 potentiates antineutrophil cytoplasmic antibody-inducing neutrophil extracellular traps formation.* Arthritis Res Ther, 2016. **18**: p. 2.

267. Meher, A.K., et al., *Novel Role of IL (Interleukin)-1beta in Neutrophil Extracellular Trap Formation and Abdominal Aortic Aneurysms.* Arterioscler Thromb Vasc Biol, 2018. **38**(4): p. 843-853.

268. Carestia, A., et al., *Mediators and molecular pathways involved in the regulation of neutrophil extracellular trap formation mediated by activated platelets.* J Leukoc Biol, 2016. **99**(1): p. 153-62.

269. Marin-Esteban, V., et al., *Afa/Dr diffusely adhering Escherichia coli strain C1845 induces neutrophil extracellular traps that kill bacteria and damage human enterocyte-like cells.* Infect Immun, 2012. **80**(5): p. 1891-9.

270. Barrientos, L., et al., *Neutrophil extracellular traps downregulate lipopolysaccharide-induced activation of monocyte-derived dendritic cells.* J Immunol, 2014. **193**(11): p. 5689-98.

271. Barquero-Calvo, E., et al., *Brucella abortus Induces the Premature Death of Human Neutrophils through the Action of Its Lipopolysaccharide.* PLoS Pathog, 2015. **11**(5): p. e1004853.

272. Caudrillier, A., et al., *Platelets induce neutrophil extracellular traps in transfusion-related acute lung injury.* J Clin Invest, 2012. **122**(7): p. 2661-71.

273. de Buhr, N., et al., *Neutrophil extracellular trap formation in the Streptococcus suis-infected cerebrospinal fluid compartment.* Cell Microbiol, 2017. **19**(2).

274. Metzler, K.D., et al., *A myeloperoxidase-containing complex regulates neutrophil elastase release and actin dynamics during NETosis.* Cell Rep, 2014. **8**(3): p. 883-96.

275. McIlroy, D.J., et al., *Mitochondrial DNA neutrophil extracellular traps are formed after trauma and subsequent surgery.* J Crit Care, 2014. **29**(6): p. 1133.e1-5.

276. Tanaka, K., et al., *In vivo characterization of neutrophil extracellular traps in various organs of a murine sepsis model.* PLoS One, 2014. **9**(11): p. e111888.

277. Kaufman, T., et al., *Nucleosomes and neutrophil extracellular traps in septic and burn patients.* Clin Immunol, 2017. **183**: p. 254-262.

278. Jaillon, S., et al., *The humoral pattern recognition receptor PTX3 is stored in neutrophil granules and localizes in extracellular traps.* J Exp Med, 2007. **204**(4): p. 793-804.

279. Pulze, L., et al., *NET amyloidogenic backbone in human activated neutrophils.* Clin Exp Immunol, 2016. **183**(3): p. 469-79.

280. Lukasova, E., et al., *Granulocyte maturation determines ability to release chromatin NETs and loss of DNA damage response; these properties are absent in immature AML granulocytes.* Biochim Biophys Acta, 2013. **1833**(3): p. 767-79.

281. Tibrewal, S., et al., *Hyperosmolar stress induces neutrophil extracellular trap formation: implications for dry eye disease.* Invest Ophthalmol Vis Sci, 2014. **55**(12): p. 7961-9.

282. Bozonet, S.M., et al., *Enhanced human neutrophil vitamin C status, chemotaxis and oxidant generation following dietary supplementation with vitamin C-rich SunGold kiwifruit.* Nutrients, 2015. **7**(4): p. 2574-88.

283. Parker, H., et al., *Myeloperoxidase associated with neutrophil extracellular traps is active and mediates bacterial killing in the presence of hydrogen peroxide.* J Leukoc Biol, 2012. **91**(3): p. 369-76.

284. Welin, A., et al., *The human neutrophil subsets defined by the presence or absence of OLFM4 both transmigrate into tissue in vivo and give rise to distinct NETs in vitro.* PLoS One, 2013. **8**(7): p. e69575.

285. Parker, H., et al., *Requirements for NADPH oxidase and myeloperoxidase in neutrophil extracellular trap formation differ depending on the stimulus.* J Leukoc Biol, 2012. **92**(4): p. 841-9.

286. Ohlsson, S.M., et al., *Neutrophils from vasculitis patients exhibit an increased propensity for activation by anti-neutrophil cytoplasmic antibodies.* Clin Exp Immunol, 2014. **176**(3): p. 363-72.

287. Hoffmann, J.H., et al., *Interindividual variation of NETosis in healthy donors: introduction and application of a refined method for extracellular trap quantification.* Exp Dermatol, 2016. **25**(11): p. 895-900.

288. Hoffmann, J.H.O., et al., *Dimethyl fumarate modulates neutrophil extracellular trap formation in a glutathione- and superoxide-dependent manner.* Br J Dermatol, 2018. **178**(1): p. 207-214.

289. Yaseen, R., et al., *Antimicrobial activity of HL-60 cells compared to primary blood-derived neutrophils against Staphylococcus aureus.* J Negat Results Biomed, 2017. **16**(1): p. 2.

290. Daigo, K., et al., *The proteomic profile of circulating pentraxin 3 (PTX3) complex in sepsis demonstrates the interaction with azurocidin 1 and other components of neutrophil extracellular traps.* Mol Cell Proteomics, 2012. **11**(6): p. M111.015073.

291. van der Linden, M., et al., *Differential Signalling and Kinetics of Neutrophil Extracellular Trap Release Revealed by Quantitative Live Imaging.* Sci Rep, 2017. **7**(1): p. 6529.

292. Donis-Maturano, L., et al., *Prolonged exposure to neutrophil extracellular traps can induce mitochondrial damage in macrophages and dendritic cells.* Springerplus, 2015. **4**: p. 161.

293. Choi, Y., et al., *Neutrophil Extracellular DNA Traps Induce Autoantigen Production by Airway Epithelial Cells.* Mediators Inflamm, 2017. **2017**: p. 5675029.

294. Kernien, J.F., C.J. Johnson, and J.E. Nett, *Conserved Inhibition of Neutrophil Extracellular Trap Release by Clinical Candida albicans Biofilms.* J Fungi (Basel), 2017. **3**(3).

295. Cortjens, B., et al., *Neutrophil extracellular traps cause airway obstruction during respiratory syncytial virus disease.* J Pathol, 2016. **238**(3): p. 401-11.

296. Tamarozzi, F., et al., *Wolbachia endosymbionts induce neutrophil extracellular trap formation in human onchocerciasis.* Sci Rep, 2016. **6**: p. 35559.

297. Zhang, X., et al., *Different virulence of candida albicans is attributed to the ability of escape from neutrophil extracellular traps by secretion of DNase.* Am J Transl Res, 2017. **9**(1): p. 50-62.

298. Savchenko, A.S., et al., *Long pentraxin 3 (PTX3) expression and release by neutrophils in vitro and in ulcerative colitis.* Pathol Int, 2011. **61**(5): p. 290-7.

299. Razvina, O., et al., *Differential expression of pentraxin 3 in neutrophils.* Exp Mol Pathol, 2015. **98**(1): p. 33-40.

300. Geerdink, R.J., et al., *LAIR-1 limits neutrophil extracellular trap formation in viral bronchiolitis.* J Allergy Clin Immunol, 2018. **141**(2): p. 811-814.

301. Paunel-Gorgulu, A., et al., *cfDNA correlates with endothelial damage after cardiac surgery with prolonged cardiopulmonary bypass and amplifies NETosis in an intracellular TLR9-independent manner.* Sci Rep, 2017. **7**(1): p. 17421.

302. Akong-Moore, K., et al., *Influences of chloride and hypochlorite on neutrophil extracellular trap formation.* PLoS One, 2012. **7**(8): p. e42984.

303. Jayaprakash, K., et al., *The role of phagocytosis, oxidative burst and neutrophil extracellular traps in the interaction between neutrophils and the periodontal pathogen Porphyromonas gingivalis.* Mol Oral Microbiol, 2015. **30**(5): p. 361-75.

304. Najmeh, S., et al., *Simplified Human Neutrophil Extracellular Traps (NETs) Isolation and Handling.* J Vis Exp, 2015(98).

305. Najmeh, S., et al., *Neutrophil extracellular traps sequester circulating tumor cells via beta1-integrin mediated interactions.* Int J Cancer, 2017. **140**(10): p. 2321-2330.

306. Sha, L.L., et al., *Autophagy is induced by anti-neutrophil cytoplasmic Abs and promotes neutrophil extracellular traps formation.* Innate Immun, 2016. **22**(8): p. 658-665.

307. Shida, H., et al., *The Presence of Anti-Lactoferrin Antibodies in a Subgroup of Eosinophilic Granulomatosis with Polyangiitis Patients and Their Possible Contribution to Enhancement of Neutrophil Extracellular Trap Formation.* Front Immunol, 2016. **7**: p. 636.

308. Kusunoki, Y., et al., *Peptidylarginine Deiminase Inhibitor Suppresses Neutrophil Extracellular Trap Formation and MPO-ANCA Production.* Front Immunol, 2016. **7**: p. 227.

309. Nakazawa, D., et al., *The responses of macrophages in interaction with neutrophils that undergo NETosis.* J Autoimmun, 2016. **67**: p. 19-28.

310. Masuda, S., et al., *Measurement of NET formation in vitro and in vivo by flow cytometry.* Cytometry A, 2017. **91**(8): p. 822-829.

311. Park, S.Y., et al., *Autophagy Primes Neutrophils for Neutrophil Extracellular Trap Formation During Sepsis.* Am J Respir Crit Care Med, 2017.

312. Shrestha, S., et al., *Retinoic acid induces hypersegmentation and enhances cytotoxicity of neutrophils against cancer cells.* Immunol Lett, 2017. **182**: p. 24-29.

313. Heddergott, C., et al., *The Arthroderma benhamiae hydrophobin HypA mediates hydrophobicity and influences recognition by human immune effector cells.* Eukaryot Cell, 2012. **11**(5): p. 673-82.

314. Proust, A., et al., *Contrasting effect of the latency-reversing agents bryostatin-1 and JQ1 on astrocyte-mediated neuroinflammation and brain neutrophil invasion.* J Neuroinflammation, 2017. **14**(1): p. 242.

315. Wang, H., et al., *Neutrophil Extracellular Trap Mitochondrial DNA and Its Autoantibody in Systemic Lupus Erythematosus and a Proof-of-Concept Trial of Metformin.* Arthritis Rheumatol, 2015. **67**(12): p. 3190-200.

316. Guo, R., et al., *A Role for Receptor-Interacting Protein Kinase-1 in Neutrophil Extracellular Trap Formation in Patients with Systemic Lupus Erythematosus: a Preliminary Study.* Cell Physiol Biochem, 2018. **45**(6): p. 2317-2328.

317. Handing, J.W. and A.K. Criss, *The lipooligosaccharide-modifying enzyme LptA enhances gonococcal defence against human neutrophils.* Cell Microbiol, 2015. **17**(6): p. 910-21.

318. Handono, K., et al., *Vitamin D prevents endothelial damage induced by increased neutrophil extracellular traps formation in patients with systemic lupus erythematosus.* Acta Med Indones, 2014. **46**(3): p. 189-98.

319. Romero, V., et al., *Immune-mediated pore-forming pathways induce cellular hypercitrullination and generate citrullinated autoantigens in rheumatoid arthritis.* Sci Transl Med, 2013. **5**(209): p. 209ra150.

320. Liu, J., et al., *A Nuclease from Streptococcus mutans Facilitates Biofilm Dispersal and Escape from Killing by Neutrophil Extracellular Traps.* Front Cell Infect Microbiol, 2017. **7**: p. 97.

321. Gomez-Lopez, N., et al., *Neutrophil Extracellular Traps in the Amniotic Cavity of Women with Intra-Amniotic Infection: A New Mechanism of Host Defense.* Reprod Sci, 2016.

322. Morita, C., et al., *Cell wall-anchored nuclease of Streptococcus sanguinis contributes to escape from neutrophil extracellular trap-mediated bacteriocidal activity.* PLoS One, 2014. **9**(8): p. e103125.

323. Ruiz-Limon, P., et al., *Tocilizumab improves the proatherothrombotic profile of rheumatoid arthritis patients modulating endothelial dysfunction, NETosis, and inflammation.* Transl Res, 2017. **183**: p. 87-103.

324. Francis, R.J., R.E. Butler, and G.R. Stewart, *Mycobacterium tuberculosis ESAT-6 is a leukocidin causing Ca2+ influx, necrosis and neutrophil extracellular trap formation.* Cell Death Dis, 2014. **5**: p. e1474.

325. Podaza, E., et al., *Neutrophils from chronic lymphocytic leukemia patients exhibit an increased capacity to release extracellular traps (NETs).* Cancer Immunol Immunother, 2017. **66**(1): p. 77-89.

326. Ventura-Juarez, J., et al., *Entamoeba histolytica induces human neutrophils to form NETs.* Parasite Immunol, 2016. **38**(8): p. 503-9.

327. Martinelli, S., et al., *Induction of genes mediating interferon-dependent extracellular trap formation during neutrophil differentiation.* J Biol Chem, 2004. **279**(42): p. 44123-32.

328. Guggino, G., et al., *Interleukin-9 over-expression and T helper 9 polarization in systemic sclerosis patients.* Clin Exp Immunol, 2017. **190**(2): p. 208-216.

329. Perez-Sanchez, C., et al., *Diagnostic potential of NETosis-derived products for disease activity, atherosclerosis and therapeutic effectiveness in Rheumatoid Arthritis patients.* J Autoimmun, 2017.

330. Kurnellas, M.P., et al., *Mechanisms of action of therapeutic amyloidogenic hexapeptides in amelioration of inflammatory brain disease.* J Exp Med, 2014. **211**(9): p. 1847-56.

331. Lindau, D., et al., *TLR9 independent interferon alpha production by neutrophils on NETosis in response to circulating chromatin, a key lupus autoantigen.* Ann Rheum Dis, 2014. **73**(12): p. 2199-207.

332. Papayannopoulos, V., et al., *Neutrophil elastase and myeloperoxidase regulate the formation of neutrophil extracellular traps.* J Cell Biol, 2010. **191**(3): p. 677-91.

333. Shimomura, Y., et al., *Recombinant human thrombomodulin inhibits neutrophil extracellular trap formation in vitro.* J Intensive Care, 2016. **4**: p. 48.

334. Raftery, M.J., et al., *beta2 integrin mediates hantavirus-induced release of neutrophil extracellular traps.* J Exp Med, 2014. **211**(7): p. 1485-97.

335. Wang, H., et al., *Neutrophil extracellular traps can activate alternative complement pathways.* Clin Exp Immunol, 2015. **181**(3): p. 518-27.

336. Vorobjeva, N.V. and B.V. Pinegin, *Effects of the antioxidants Trolox, Tiron and Tempol on neutrophil extracellular trap formation.* Immunobiology, 2016. **221**(2): p. 208-19.

337. Rodriguez-Rodrigues, N., et al., *Prokaryotic RNA Associated to Bacterial Viability Induces Polymorphonuclear Neutrophil Activation.* Front Cell Infect Microbiol, 2017. **7**: p. 306.

338. Aleyd, E., et al., *IgA Complexes in Plasma and Synovial Fluid of Patients with Rheumatoid Arthritis Induce Neutrophil Extracellular Traps via FcalphaRI.* J Immunol, 2016. **197**(12): p. 4552-4559.

339. Aleyd, E., et al., *IgA enhances NETosis and release of neutrophil extracellular traps by polymorphonuclear cells via Fcalpha receptor I.* J Immunol, 2014. **192**(5): p. 2374-83.

340. van Sorge, N.M., et al., *Methicillin-resistant Staphylococcus aureus bacterial nitric-oxide synthase affects antibiotic sensitivity and skin abscess development.* J Biol Chem, 2013. **288**(9): p. 6417-26.

341. Berends, E.T., et al., *Nuclease expression by Staphylococcus aureus facilitates escape from neutrophil extracellular traps.* J Innate Immun, 2010. **2**(6): p. 576-86.

342. Secundino, I., et al., *Host and pathogen hyaluronan signal through human siglec-9 to suppress neutrophil activation.* J Mol Med (Berl), 2016. **94**(2): p. 219-33.

343. Joshi, M.B., et al., *High glucose modulates IL-6 mediated immune homeostasis through impeding neutrophil extracellular trap formation.* FEBS Lett, 2013. **587**(14): p. 2241-6.

344. Farley, K., et al., *A serpinB1 regulatory mechanism is essential for restricting neutrophil extracellular trap generation.* J Immunol, 2012. **189**(9): p. 4574-81.

345. Johnson, C.J., et al., *The Extracellular Matrix of Candida albicans Biofilms Impairs Formation of Neutrophil Extracellular Traps.* PLoS Pathog, 2016. **12**(9): p. e1005884.

346. Pham, D.L., et al., *Neutrophil autophagy and extracellular DNA traps contribute to airway inflammation in severe asthma.* Clin Exp Allergy, 2017. **47**(1): p. 57-70.

347. Johnson, C.J., et al., *Mechanisms involved in the triggering of neutrophil extracellular traps (NETs) by Candida glabrata during planktonic and biofilm growth.* Sci Rep, 2017. **7**(1): p. 13065.

348. Wong, S.L., et al., *Diabetes primes neutrophils to undergo NETosis, which impairs wound healing.* Nat Med, 2015. **21**(7): p. 815-9.

349. Hashiba, M., et al., *Neutrophil extracellular traps in patients with sepsis.* J Surg Res, 2015. **194**(1): p. 248-54.

350. Remijsen, Q., et al., *Neutrophil extracellular trap cell death requires both autophagy and superoxide generation.* Cell Res, 2011. **21**(2): p. 290-304.

351. Zhu, L., et al., *High Level of Neutrophil Extracellular Traps Correlates With Poor Prognosis of Severe Influenza A Infection.* J Infect Dis, 2018. **217**(3): p. 428-437.

352. Gunderson, C.W. and H.S. Seifert, *Neisseria gonorrhoeae elicits extracellular traps in primary neutrophil culture while suppressing the oxidative burst.* MBio, 2015. **6**(1).

353. Romao, S., et al., *Defective nuclear entry of hydrolases prevents neutrophil extracellular trap formation in patients with chronic granulomatous disease.* J Allergy Clin Immunol, 2015. **136**(6): p. 1703-6.e1-5.

354. Bruns, S., et al., *Production of extracellular traps against Aspergillus fumigatus in vitro and in infected lung tissue is dependent on invading neutrophils and influenced by hydrophobin RodA.* PLoS Pathog, 2010. **6**(4): p. e1000873.

355. Meng, W., et al., *Deoxyribonuclease is a potential counter regulator of aberrant neutrophil extracellular traps formation after major trauma.* Mediators Inflamm, 2012. **2012**: p. 149560.

356. Eby, J.C., M.C. Gray, and E.L. Hewlett, *Cyclic AMP-mediated suppression of neutrophil extracellular trap formation and apoptosis by the Bordetella pertussis adenylate cyclase toxin.* Infect Immun, 2014. **82**(12): p. 5256-69.

357. Calo, G., et al., *Trophoblast cells inhibit neutrophil extracellular trap formation and enhance apoptosis through vasoactive intestinal peptide-mediated pathways.* Hum Reprod, 2017. **32**(1): p. 55-64.

358. Della Coletta, A.M., et al., *Neutrophil Extracellular Traps Identification in Tegumentary Lesions of Patients with Paracoccidioidomycosis and Different Patterns of NETs Generation In Vitro.* PLoS Negl Trop Dis, 2015. **9**(9): p. e0004037.

359. Bachiega, T.F., et al., *Participation of dectin-1 receptor on NETs release against Paracoccidioides brasiliensis: Role on extracellular killing.* Immunobiology, 2016. **221**(2): p. 228-35.

360. Sabbione, F., et al., *Neutrophil Extracellular Traps Stimulate Proinflammatory Responses in Human Airway Epithelial Cells.* J Innate Immun, 2017.

361. Gorgojo, J., et al., *Bordetella parapertussis Circumvents Neutrophil Extracellular Bactericidal Mechanisms.* PLoS One, 2017. **12**(1): p. e0169936.

362. Branzk, N., et al., *Neutrophils sense microbe size and selectively release neutrophil extracellular traps in response to large pathogens.* Nat Immunol, 2014. **15**(11): p. 1017-25.

363. Millrud, C.R., et al., *NET-producing CD16high CD62Ldim neutrophils migrate to tumor sites and predict improved survival in patients with HNSCC.* Int J Cancer, 2017. **140**(11): p. 2557-2567.

364. Schorn, C., et al., *Bonding the foe - NETting neutrophils immobilize the pro-inflammatory monosodium urate crystals.* Front Immunol, 2012. **3**: p. 376.

365. Cole, J.N., et al., *M protein and hyaluronic acid capsule are essential for in vivo selection of covRS mutations characteristic of invasive serotype M1T1 group A Streptococcus.* MBio, 2010. **1**(4).

366. Young, R.L., et al., *Neutrophil extracellular trap (NET)-mediated killing of Pseudomonas aeruginosa: evidence of acquired resistance within the CF airway, independent of CFTR.* PLoS One, 2011. **6**(9): p. e23637.

367. Lauth, X., et al., *M1 protein allows Group A streptococcal survival in phagocyte extracellular traps through cathelicidin inhibition.* J Innate Immun, 2009. **1**(3): p. 202-14.

368. Chuah, C., et al., *Defining a pro-inflammatory neutrophil phenotype in response to schistosome eggs.* Cell Microbiol, 2014. **16**(11): p. 1666-77.

369. Buchanan, J.T., et al., *DNase expression allows the pathogen group A Streptococcus to escape killing in neutrophil extracellular traps.* Curr Biol, 2006. **16**(4): p. 396-400.

370. Fetz, A.E., et al., *Localized Delivery of Cl-Amidine From Electrospun Polydioxanone Templates to Regulate Acute Neutrophil NETosis: A Preliminary Evaluation of the PAD4 Inhibitor for Tissue Engineering.* Front Pharmacol, 2018. **9**: p. 289.

371. Sil, P., et al., *High Throughput Measurement of Extracellular DNA Release and Quantitative NET Formation in Human Neutrophils In Vitro.* J Vis Exp, 2016(112).

372. Floyd, M., et al., *Swimming Motility Mediates the Formation of Neutrophil Extracellular Traps Induced by Flagellated Pseudomonas aeruginosa.* PLoS Pathog, 2016. **12**(11): p. e1005987.

373. Sil, P., et al., *P2Y6 Receptor Antagonist MRS2578 Inhibits Neutrophil Activation and Aggregated Neutrophil Extracellular Trap Formation Induced by Gout-Associated Monosodium Urate Crystals.* J Immunol, 2017. **198**(1): p. 428-442.

374. Sil, P., et al., *Macrophage-derived IL-1beta enhances monosodium urate crystal-triggered NET formation.* Inflamm Res, 2017. **66**(3): p. 227-237.

375. Itoh, H., et al., *Enhancement of neutrophil autophagy by an IVIG preparation against multidrug-resistant bacteria as well as drug-sensitive strains.* J Leukoc Biol, 2015. **98**(1): p. 107-17.

376. Lood, C., et al., *Neutrophil extracellular traps enriched in oxidized mitochondrial DNA are interferogenic and contribute to lupus-like disease.* Nat Med, 2016. **22**(2): p. 146-53.

377. Garcia-Romo, G.S., et al., *Netting neutrophils are major inducers of type I IFN production in pediatric systemic lupus erythematosus.* Sci Transl Med, 2011. **3**(73): p. 73ra20.

378. Kamoshida, G., et al., *Pathogenic Bacterium Acinetobacter baumannii Inhibits the Formation of Neutrophil Extracellular Traps by Suppressing Neutrophil Adhesion.* Front Immunol, 2018. **9**: p. 178.

379. Wright, H.L., et al., *Low-density granulocytes: functionally distinct, immature neutrophils in rheumatoid arthritis with altered properties and defective TNF signalling.* J Leukoc Biol, 2017. **101**(2): p. 599-611.

380. Konstantinidis, T., et al., *Immunomodulatory Role of Clarithromycin in Acinetobacter baumannii Infection via Formation of Neutrophil Extracellular Traps.* Antimicrob Agents Chemother, 2016. **60**(2): p. 1040-8.

381. Kamoshida, G., et al., *Acinetobacter baumannii escape from neutrophil extracellular traps (NETs).* J Infect Chemother, 2015. **21**(1): p. 43-9.

382. Casutt-Meyer, S., et al., *Oligomeric coiled-coil adhesin YadA is a double-edged sword.* PLoS One, 2010. **5**(12): p. e15159.

383. Chrysanthopoulou, A., et al., *Interferon lambda1/IL-29 and inorganic polyphosphate are novel regulators of neutrophil-driven thromboinflammation.* J Pathol, 2017. **243**(1): p. 111-122.

384. Bartneck, M., et al., *Phagocytosis independent extracellular nanoparticle clearance by human immune cells.* Nano Lett, 2010. **10**(1): p. 59-63.

385. Kambas, K., et al., *Autophagy mediates the delivery of thrombogenic tissue factor to neutrophil extracellular traps in human sepsis.* PLoS One, 2012. **7**(9): p. e45427.

386. Haute, G.V., et al., *Gallic acid reduces the effect of LPS on apoptosis and inhibits the formation of neutrophil extracellular traps.* Toxicol In Vitro, 2015. **30**(1 Pt B): p. 309-17.

387. Chen, S.T., et al., *CLEC5A is a critical receptor in innate immunity against Listeria infection.* Nat Commun, 2017. **8**(1): p. 299.

388. Lee, I.R., et al., *Comparison of Diabetic and Non-diabetic Human Leukocytic Responses to Different Capsule Types of Klebsiella pneumoniae Responsible for Causing Pyogenic Liver Abscess.* Front Cell Infect Microbiol, 2017. **7**: p. 401.

389. Wang, L., et al., *Resistance of hypervirulent Klebsiella pneumoniae to both intracellular and extracellular killing of neutrophils.* PLoS One, 2017. **12**(3): p. e0173638.

390. Bonne-Annee, S., et al., *Extracellular traps are associated with human and mouse neutrophil and macrophage mediated killing of larval Strongyloides stercoralis.* Microbes Infect, 2014. **16**(6): p. 502-11.

391. Fetz, A.E., et al., *Electrospun Template Architecture and Composition Regulate Neutrophil NETosis In Vitro and In Vivo.* Tissue Eng Part A, 2017.

392. Chang, H.H., et al., *The W620 Polymorphism in PTPN22 Disrupts Its Interaction With Peptidylarginine Deiminase Type 4 and Enhances Citrullination and NETosis.* Arthritis Rheumatol, 2015. **67**(9): p. 2323-34.

393. Neeli, I., et al., *Regulation of extracellular chromatin release from neutrophils.* J Innate Immun, 2009. **1**(3): p. 194-201.

394. Neeli, I., S.N. Khan, and M. Radic, *Histone deimination as a response to inflammatory stimuli in neutrophils.* J Immunol, 2008. **180**(3): p. 1895-902.

395. Dwivedi, N., et al., *Felty's syndrome autoantibodies bind to deiminated histones and neutrophil extracellular chromatin traps.* Arthritis Rheum, 2012. **64**(4): p. 982-92.

396. Fuchs, T.A., et al., *Novel cell death program leads to neutrophil extracellular traps.* J Cell Biol, 2007. **176**(2): p. 231-41.

397. Crotty Alexander, L.E., et al., *M1T1 group A streptococcal pili promote epithelial colonization but diminish systemic virulence through neutrophil extracellular entrapment.* J Mol Med (Berl), 2010. **88**(4): p. 371-81.

398. Gupta, A.K., et al., *Activated endothelial cells induce neutrophil extracellular traps and are susceptible to NETosis-mediated cell death.* FEBS Lett, 2010. **584**(14): p. 3193-7.

399. Wartha, F., et al., *Capsule and D-alanylated lipoteichoic acids protect Streptococcus pneumoniae against neutrophil extracellular traps.* Cell Microbiol, 2007. **9**(5): p. 1162-71.

400. Beiter, K., et al., *An endonuclease allows Streptococcus pneumoniae to escape from neutrophil extracellular traps.* Curr Biol, 2006. **16**(4): p. 401-7.

401. Joshi, M.B., et al., *Elevated homocysteine levels in type 2 diabetes induce constitutive neutrophil extracellular traps.* Sci Rep, 2016. **6**: p. 36362.

402. Menegazzi, R., E. Decleva, and P. Dri, *Killing by neutrophil extracellular traps: fact or folklore?* Blood, 2012. **119**(5): p. 1214-6.

403. Gupta, A.K., et al., *Efficient neutrophil extracellular trap induction requires mobilization of both intracellular and extracellular calcium pools and is modulated by cyclosporine A.* PLoS One, 2014. **9**(5): p. e97088.

404. Ullah, I., N.D. Ritchie, and T.J. Evans, *The interrelationship between phagocytosis, autophagy and formation of neutrophil extracellular traps following infection of human neutrophils by Streptococcus pneumoniae.* Innate Immun, 2017: p. 1753425917704299.

405. Gabriel, C., et al., *Leishmania donovani promastigotes evade the antimicrobial activity of neutrophil extracellular traps.* J Immunol, 2010. **185**(7): p. 4319-27.

406. Brinkmann, V., et al., *Neutrophil extracellular traps kill bacteria.* Science, 2004. **303**(5663): p. 1532-5.

407. Kenny, E.F., et al., *Diverse stimuli engage different neutrophil extracellular trap pathways.* Elife, 2017. **6**.

408. Mohanty, T., et al., *A novel mechanism for NETosis provides antimicrobial defense at the oral mucosa.* Blood, 2015. **126**(18): p. 2128-37.

409. Mohanty, T., O.E. Sorensen, and P. Nordenfelt, *NETQUANT: Automated Quantification of Neutrophil Extracellular Traps.* Front Immunol, 2017. **8**: p. 1999.

410. Sollberger, G., B. Amulic, and A. Zychlinsky, *Neutrophil Extracellular Trap Formation Is Independent of De Novo Gene Expression.* PLoS One, 2016. **11**(6): p. e0157454.

411. Boneschansker, L., et al., *Capillary plexuses are vulnerable to neutrophil extracellular traps.* Integr Biol (Camb), 2016. **8**(2): p. 149-55.

412. Kirchner, T., et al., *The impact of various reactive oxygen species on the formation of neutrophil extracellular traps.* Mediators Inflamm, 2012. **2012**: p. 849136.

413. Kirchner, T., et al., *Flavonoids and 5-aminosalicylic acid inhibit the formation of neutrophil extracellular traps.* Mediators Inflamm, 2013. **2013**: p. 710239.

414. Behnen, M., et al., *Extracellular Acidification Inhibits the ROS-Dependent Formation of Neutrophil Extracellular Traps.* Front Immunol, 2017. **8**: p. 184.

415. Hu, S.C., et al., *Neutrophil extracellular trap formation is increased in psoriasis and induces human beta-defensin-2 production in epidermal keratinocytes.* Sci Rep, 2016. **6**: p. 31119.

416. Brinkmann, V., et al., *Automatic quantification of in vitro NET formation.* Front Immunol, 2012. **3**: p. 413.

417. Fuxman Bass, J.I., et al., *Extracellular DNA: a major proinflammatory component of Pseudomonas aeruginosa biofilms.* J Immunol, 2010. **184**(11): p. 6386-95.

418. Abi Abdallah, D.S., et al., *Toxoplasma gondii triggers release of human and mouse neutrophil extracellular traps.* Infect Immun, 2012. **80**(2): p. 768-77.

419. van de Geer, A., et al., *Characterization of buffy coat-derived granulocytes for clinical use: a comparison with granulocyte colony-stimulating factor/dexamethasone-pretreated donor-derived products.* Vox Sang, 2017. **112**(2): p. 173-182.

420. Ermert, D., A. Zychlinsky, and C. Urban, *Fungal and bacterial killing by neutrophils.* Methods Mol Biol, 2009. **470**: p. 293-312.

421. Lappann, M., et al., *In vitro resistance mechanisms of Neisseria meningitidis against neutrophil extracellular traps.* Mol Microbiol, 2013. **89**(3): p. 433-49.

422. Avila, E.E., et al., *Entamoeba histolytica Trophozoites and Lipopeptidophosphoglycan Trigger Human Neutrophil Extracellular Traps.* PLoS One, 2016. **11**(7): p. e0158979.

423. Carey, A.J., et al., *Infection and cellular defense dynamics in a novel 17beta-estradiol murine model of chronic human group B streptococcus genital tract colonization reveal a role for hemolysin in persistence and neutrophil accumulation.* J Immunol, 2014. **192**(4): p. 1718-31.

424. Brinkmann, V., et al., *Neutrophil extracellular traps: how to generate and visualize them.* J Vis Exp, 2010(36).

425. Thammavongsa, V., D.M. Missiakas, and O. Schneewind, *Staphylococcus aureus degrades neutrophil extracellular traps to promote immune cell death.* Science, 2013. **342**(6160): p. 863-6.

426. Chang, A., et al., *Functional analysis of Streptococcus pyogenes nuclease A (SpnA), a novel group A streptococcal virulence factor.* Mol Microbiol, 2011. **79**(6): p. 1629-42.

427. Gould, T.J., et al., *Neutrophil extracellular traps promote thrombin generation through platelet-dependent and platelet-independent mechanisms.* Arterioscler Thromb Vasc Biol, 2014. **34**(9): p. 1977-84.

428. Swethakumar, B., et al., *Inhibition of Echis carinatus venom by DNA, a promising therapeutic molecule for snakebite management.* Biochim Biophys Acta, 2018. **1862**(5): p. 1115-1125.

429. Katkar, G.D., et al., *NETosis and lack of DNase activity are key factors in Echis carinatus venom-induced tissue destruction.* Nat Commun, 2016. **7**: p. 11361.

430. Amini, P., et al., *NET formation can occur independently of RIPK3 and MLKL signaling.* Eur J Immunol, 2016. **46**(1): p. 178-84.

431. Abdol Razak, N., O. Elaskalani, and P. Metharom, *Pancreatic Cancer-Induced Neutrophil Extracellular Traps: A Potential Contributor to Cancer-Associated Thrombosis.* Int J Mol Sci, 2017. **18**(3).

432. Germic, N., et al., *Neither eosinophils nor neutrophils require ATG5-dependent autophagy for extracellular DNA trap formation.* Immunology, 2017. **152**(3): p. 517-525.

433. Stojkov, D., et al., *ROS and glutathionylation balance cytoskeletal dynamics in neutrophil extracellular trap formation.* J Cell Biol, 2017. **216**(12): p. 4073-4090.

434. Okubo, K., et al., *Lactoferrin Suppresses Neutrophil Extracellular Traps Release in Inflammation.* EBioMedicine, 2016. **10**: p. 204-15.

435. Curcic, S., et al., *Neutrophil effector responses are suppressed by secretory phospholipase A2 modified HDL.* Biochim Biophys Acta, 2015. **1851**(2): p. 184-93.

436. Ramos, M.V., et al., *Induction of Neutrophil Extracellular Traps in Shiga Toxin-Associated Hemolytic Uremic Syndrome.* J Innate Immun, 2016. **8**(4): p. 400-11.

437. Clancy, D.M., et al., *Neutrophil extracellular traps can serve as platforms for processing and activation of IL-1 family cytokines.* Febs j, 2017. **284**(11): p. 1712-1725.

438. Sandlin, R.D., et al., *Preservative solution that stabilizes erythrocyte morphology and leukocyte viability under ambient conditions.* Sci Rep, 2017. **7**(1): p. 5658.

439. Weckmann, M., et al., *Treatment with rhDNase in patients with cystic fibrosis alters in-vitro CHIT-1 activity of isolated leucocytes.* Clin Exp Immunol, 2016. **185**(3): p. 382-91.

440. Kessenbrock, K., et al., *Netting neutrophils in autoimmune small-vessel vasculitis.* Nat Med, 2009. **15**(6): p. 623-5.

441. Chatfield, S.M., et al., *Monosodium Urate Crystals Generate Nuclease-Resistant Neutrophil Extracellular Traps via a Distinct Molecular Pathway.* J Immunol, 2018. **200**(5): p. 1802-1816.

442. Armstrong, C.L., et al., *Filifactor alocis manipulates human neutrophils affecting their ability to release neutrophil extracellular traps induced by PMA.* Innate Immun, 2018: p. 1753425918767507.

443. Ostafin, M., et al., *Different procedures of diphenyleneiodonium chloride addition affect neutrophil extracellular trap formation.* Anal Biochem, 2016. **509**: p. 60-6.

444. Boldenow, E., et al., *GROUP B STREPTOCOCCUS CIRCUMVENTS NEUTROPHILS AND NEUTROPHIL EXTRACELLULAR TRAPS DURING AMNIOTIC CAVITY INVASION AND PRETERM LABOR.* Sci Immunol, 2016. **1**(4).

445. Alghamdi, A.S. and D.N. Foster, *Seminal DNase frees spermatozoa entangled in neutrophil extracellular traps.* Biol Reprod, 2005. **73**(6): p. 1174-81.

446. Itakura, A. and O.J. McCarty, *Pivotal role for the mTOR pathway in the formation of neutrophil extracellular traps via regulation of autophagy.* Am J Physiol Cell Physiol, 2013. **305**(3): p. C348-54.

447. Healy, L.D., et al., *Colocalization of neutrophils, extracellular DNA and coagulation factors during NETosis: Development and utility of an immunofluorescence-based microscopy platform.* J Immunol Methods, 2016. **435**: p. 77-84.

448. Healy, L.D., et al., *Activated protein C inhibits neutrophil extracellular trap formation in vitro and activation in vivo.* J Biol Chem, 2017. **292**(21): p. 8616-8629.

449. Mor-Vaknin, N., et al., *DEK-targeting DNA aptamers as therapeutics for inflammatory arthritis.* Nat Commun, 2017. **8**: p. 14252.

450. Lin, A.M., et al., *Mast cells and neutrophils release IL-17 through extracellular trap formation in psoriasis.* J Immunol, 2011. **187**(1): p. 490-500.

451. Villanueva, E., et al., *Netting neutrophils induce endothelial damage, infiltrate tissues, and expose immunostimulatory molecules in systemic lupus erythematosus.* J Immunol, 2011. **187**(1): p. 538-52.

452. Lapponi, M.J., et al., *Regulation of neutrophil extracellular trap formation by anti-inflammatory drugs.* J Pharmacol Exp Ther, 2013. **345**(3): p. 430-7.

453. Shishikura, K., et al., *Prostaglandin E2 inhibits neutrophil extracellular trap formation through production of cyclic AMP.* Br J Pharmacol, 2016. **173**(2): p. 319-31.

454. Wan, T., et al., *Dexamethasone Inhibits S. aureus-Induced Neutrophil Extracellular Pathogen-Killing Mechanism, Possibly through Toll-Like Receptor Regulation.* Front Immunol, 2017. **8**: p. 60.

455. Scharrig, E., et al., *Neutrophil Extracellular Traps are Involved in the Innate Immune Response to Infection with Leptospira.* PLoS Negl Trop Dis, 2015. **9**(7): p. e0003927.

456. Li, H., et al., *Mitochondrial damage-associated molecular patterns from fractures suppress pulmonary immune responses via formyl peptide receptors 1 and 2.* J Trauma Acute Care Surg, 2015. **78**(2): p. 272-9; discussion 279-81.

457. Sorensen, O.E., et al., *Papillon-Lefevre syndrome patient reveals species-dependent requirements for neutrophil defenses.* J Clin Invest, 2014. **124**(10): p. 4539-48.

458. Gazendam, R.P., et al., *Human Neutrophils Use Different Mechanisms To Kill Aspergillus fumigatus Conidia and Hyphae: Evidence from Phagocyte Defects.* J Immunol, 2016. **196**(3): p. 1272-83.

459. Cheng, M.L., et al., *Effective NET formation in neutrophils from individuals with G6PD Taiwan-Hakka is associated with enhanced NADP(+) biosynthesis.* Free Radic Res, 2013. **47**(9): p. 699-709.

460. Arelaki, S., et al., *Gradient Infiltration of Neutrophil Extracellular Traps in Colon Cancer and Evidence for Their Involvement in Tumour Growth.* PLoS One, 2016. **11**(5): p. e0154484.

**References for murine NETosis-experiments**

1. Grund, L.Z., et al., *Neutrophils releasing IL-17A into NETs are essential to plasma cell differentiation in inflamed tissue dependent on IL-1R.* Autoimmunity, 2017. **50**(2): p. 86-101.

2. Watanabe, M., et al., *DOCK2 and DOCK5 act additively in neutrophils to regulate chemotaxis, superoxide production, and extracellular trap formation.* J Immunol, 2014. **193**(11): p. 5660-7.

3. Neumann, A., et al., *The antimicrobial peptide LL-37 facilitates the formation of neutrophil extracellular traps.* Biochem J, 2014. **464**(1): p. 3-11.

4. Hayashi, H., et al., *Sirt3 deficiency does not affect venous thrombosis or NETosis despite mild elevation of intracellular ROS in platelets and neutrophils in mice.* PLoS One, 2017. **12**(12): p. e0188341.

5. Khan, Z., et al., *Angiotensin converting enzyme enhances the oxidative response and bactericidal activity of neutrophils.* Blood, 2017.

6. Luo, L., et al., *Proinflammatory role of neutrophil extracellular traps in abdominal sepsis.* Am J Physiol Lung Cell Mol Physiol, 2014. **307**(7): p. L586-96.

7. Merza, M., et al., *Neutrophil Extracellular Traps Induce Trypsin Activation, Inflammation, and Tissue Damage in Mice With Severe Acute Pancreatitis.* Gastroenterology, 2015. **149**(7): p. 1920-1931.e8.

8. Landoni, V.I., et al., *Tolerance to lipopolysaccharide promotes an enhanced neutrophil extracellular traps formation leading to a more efficient bacterial clearance in mice.* Clin Exp Immunol, 2012. **168**(1): p. 153-63.

9. Saha, P., et al., *Bacterial Siderophores Hijack Neutrophil Functions.* J Immunol, 2017. **198**(11): p. 4293-4303.

10. Farley, K., et al., *A serpinB1 regulatory mechanism is essential for restricting neutrophil extracellular trap generation.* J Immunol, 2012. **189**(9): p. 4574-81.

11. Domingo-Gonzalez, R., et al., *Inhibition of Neutrophil Extracellular Trap Formation after Stem Cell Transplant by Prostaglandin E2.* Am J Respir Crit Care Med, 2016. **193**(2): p. 186-97.

12. Gavillet, M., et al., *Flow cytometric assay for direct quantification of neutrophil extracellular traps in blood samples.* Am J Hematol, 2015. **90**(12): p. 1155-8.

13. Jacob, C.O., et al., *Haploinsufficiency of NADPH oxidase subunit NCF2 is sufficient to accelerate full-blown lupus in NZM.2328 mice.* Arthritis Rheumatol, 2017.

14. Smith, C.K., et al., *Neutrophil extracellular trap-derived enzymes oxidize high-density lipoprotein: an additional proatherogenic mechanism in systemic lupus erythematosus.* Arthritis Rheumatol, 2014. **66**(9): p. 2532-44.

15. Martinod, K., et al., *Neutrophil elastase-deficient mice form neutrophil extracellular traps in an experimental model of deep vein thrombosis.* J Thromb Haemost, 2016. **14**(3): p. 551-8.

16. Gavillet, M., et al., *A key role for Rac and Pak signaling in neutrophil extracellular traps (NETs) formation defines a new potential therapeutic target.* Am J Hematol, 2018. **93**(2): p. 269-276.

17. Qiu, S.L., et al., *Neutrophil extracellular traps induced by cigarette smoke activate plasmacytoid dendritic cells.* Thorax, 2017. **72**(12): p. 1084-1093.

18. Zhao, J., et al., *Streptococcus suis serotype 2 strains can induce the formation of neutrophil extracellular traps and evade trapping.* FEMS Microbiol Lett, 2015. **362**(6).

19. Biermann, M.H., et al., *Oxidative Burst-Dependent NETosis Is Implicated in the Resolution of Necrosis-Associated Sterile Inflammation.* Front Immunol, 2016. **7**: p. 557.

20. Etulain, J., et al., *P-selectin promotes neutrophil extracellular trap formation in mice.* Blood, 2015. **126**(2): p. 242-6.

21. Carmona-Rivera, C., et al., *A role for muscarinic receptors in neutrophil extracellular trap formation and levamisole-induced autoimmunity.* JCI Insight, 2017. **2**(3): p. e89780.

22. Desai, J., et al., *PMA and crystal-induced neutrophil extracellular trap formation involves RIPK1-RIPK3-MLKL signaling.* Eur J Immunol, 2016. **46**(1): p. 223-9.

23. Ma, F., X. Guo, and H. Fan, *Extracellular Nucleases of Streptococcus equi subsp. zooepidemicus Degrade Neutrophil Extracellular Traps and Impair Macrophage Activity of the Host.* Appl Environ Microbiol, 2017. **83**(2).

24. Hu, Z., et al., *Neutrophil extracellular traps induce IL-1beta production by macrophages in combination with lipopolysaccharide.* Int J Mol Med, 2017.

25. Furumoto, Y., et al., *Tofacitinib Ameliorates Murine Lupus and Its Associated Vascular Dysfunction.* Arthritis Rheumatol, 2017. **69**(1): p. 148-160.

26. Martinod, K., et al., *Neutrophil histone modification by peptidylarginine deiminase 4 is critical for deep vein thrombosis in mice.* Proc Natl Acad Sci U S A, 2013. **110**(21): p. 8674-9.

27. Bawadekar, M., et al., *Peptidylarginine deiminase 2 is required for tumor necrosis factor alpha-induced citrullination and arthritis, but not neutrophil extracellular trap formation.* J Autoimmun, 2017. **80**: p. 39-47.

28. Wong, S.L., et al., *Diabetes primes neutrophils to undergo NETosis, which impairs wound healing.* Nat Med, 2015. **21**(7): p. 815-9.

29. Das, S.K., Y.F. Yuan, and M.Q. Li, *Specific PKC beta II Inhibitor: One Stone Two Birds in the Treatment of Diabetic Foot Ulcers.* Biosci Rep, 2018.

30. Lewis, H.D., et al., *Inhibition of PAD4 activity is sufficient to disrupt mouse and human NET formation.* Nat Chem Biol, 2015. **11**(3): p. 189-91.

31. Savchenko, A.S., et al., *VWF-mediated leukocyte recruitment with chromatin decondensation by PAD4 increases myocardial ischemia/reperfusion injury in mice.* Blood, 2014. **123**(1): p. 141-8.

32. Moorthy, A.N., et al., *Capsules of virulent pneumococcal serotypes enhance formation of neutrophil extracellular traps during in vivo pathogenesis of pneumonia.* Oncotarget, 2016. **7**(15): p. 19327-40.

33. Juneau, R.A., et al., *Nontypeable Haemophilus influenzae initiates formation of neutrophil extracellular traps.* Infect Immun, 2011. **79**(1): p. 431-8.

34. Rohm, M., et al., *NADPH oxidase promotes neutrophil extracellular trap formation in pulmonary aspergillosis.* Infect Immun, 2014. **82**(5): p. 1766-77.

35. Chang, Z., et al., *The TatD-like DNase of Plasmodium is a virulence factor and a potential malaria vaccine candidate.* Nat Commun, 2016. **7**: p. 11537.

36. Desai, J., et al., *Particles of different sizes and shapes induce neutrophil necroptosis followed by the release of neutrophil extracellular trap-like chromatin.* Sci Rep, 2017. **7**(1): p. 15003.

37. Hamaguchi, S., et al., *Origin of Circulating Free DNA in Sepsis: Analysis of the CLP Mouse Model.* Mediators Inflamm, 2015. **2015**: p. 614518.

38. Arroyo, A.B., et al., *MiR-146a Regulates Neutrophil Extracellular Trap Formation That Predicts Adverse Cardiovascular Events in Patients With Atrial Fibrillation.* Arterioscler Thromb Vasc Biol, 2018. **38**(4): p. 892-902.

39. Nani, S., et al., *Src family kinases and Syk are required for neutrophil extracellular trap formation in response to beta-glucan particles.* J Innate Immun, 2015. **7**(1): p. 59-73.

40. Iversen, M.B., et al., *Extracellular superoxide dismutase is present in secretory vesicles of human neutrophils and released upon stimulation.* Free Radic Biol Med, 2016. **97**: p. 478-88.

41. Vong, L., et al., *Probiotic Lactobacillus rhamnosus inhibits the formation of neutrophil extracellular traps.* J Immunol, 2014. **192**(4): p. 1870-7.

42. Lim, M.B., et al., *Rac2 is required for the formation of neutrophil extracellular traps.* J Leukoc Biol, 2011. **90**(4): p. 771-6.

43. Braster, Q., et al., *Inhibition of NET Release Fails to Reduce Adipose Tissue Inflammation in Mice.* PLoS One, 2016. **11**(10): p. e0163922.

44. Chen, G., et al., *Heme-induced neutrophil extracellular traps contribute to the pathogenesis of sickle cell disease.* Blood, 2014. **123**(24): p. 3818-27.

45. Maueroder, C., et al., *Capability of Neutrophils to Form NETs Is Not Directly Influenced by a CMA-Targeting Peptide.* Front Immunol, 2017. **8**: p. 16.

46. Westhorpe, C.L., et al., *In Vivo Imaging of Inflamed Glomeruli Reveals Dynamics of Neutrophil Extracellular Trap Formation in Glomerular Capillaries.* Am J Pathol, 2017. **187**(2): p. 318-331.

47. Barletta, K.E., et al., *Adenosine A(2B) receptor deficiency promotes host defenses against gram-negative bacterial pneumonia.* Am J Respir Crit Care Med, 2012. **186**(10): p. 1044-50.

48. Vong, L., et al., *Selective enrichment of commensal gut bacteria protects against Citrobacter rodentium-induced colitis.* Am J Physiol Gastrointest Liver Physiol, 2015. **309**(3): p. G181-92.

49. Vong, L., et al., *Adherent-invasive Escherichia coli Exacerbates Antibiotic-associated Intestinal Dysbiosis and Neutrophil Extracellular Trap Activation.* Inflamm Bowel Dis, 2016. **22**(1): p. 42-54.

50. Dwyer, M., et al., *Cystic fibrosis sputum DNA has NETosis characteristics and neutrophil extracellular trap release is regulated by macrophage migration-inhibitory factor.* J Innate Immun, 2014. **6**(6): p. 765-79.

51. Boone, B.A., et al., *The receptor for advanced glycation end products (RAGE) enhances autophagy and neutrophil extracellular traps in pancreatic cancer.* Cancer Gene Ther, 2015. **22**(6): p. 326-34.

52. Corriden, R., et al., *Tamoxifen augments the innate immune function of neutrophils through modulation of intracellular ceramide.* Nat Commun, 2015. **6**: p. 8369.

53. Park, J., et al., *Cancer cells induce metastasis-supporting neutrophil extracellular DNA traps.* Sci Transl Med, 2016. **8**(361): p. 361ra138.

54. Thomas, D.C., et al., *Eros is a novel transmembrane protein that controls the phagocyte respiratory burst and is essential for innate immunity.* J Exp Med, 2017. **214**(4): p. 1111-1128.

55. Germic, N., et al., *Neither eosinophils nor neutrophils require ATG5-dependent autophagy for extracellular DNA trap formation.* Immunology, 2017. **152**(3): p. 517-525.

56. Li, P., et al., *PAD4 is essential for antibacterial innate immunity mediated by neutrophil extracellular traps.* J Exp Med, 2010. **207**(9): p. 1853-62.

57. Leal, A.C., et al., *Tumor-Derived Exosomes Induce the Formation of Neutrophil Extracellular Traps: Implications For The Establishment of Cancer-Associated Thrombosis.* Sci Rep, 2017. **7**(1): p. 6438.

58. Maugeri, N., et al., *Activated platelets present high mobility group box 1 to neutrophils, inducing autophagy and promoting the extrusion of neutrophil extracellular traps.* J Thromb Haemost, 2014. **12**(12): p. 2074-88.

59. Stavrou, E.X., et al., *Factor XII and uPAR upregulate neutrophil functions to influence wound healing.* J Clin Invest, 2018. **128**(3): p. 944-959.

60. Mor-Vaknin, N., et al., *DEK-targeting DNA aptamers as therapeutics for inflammatory arthritis.* Nat Commun, 2017. **8**: p. 14252.

61. Kimura, H., et al., *The effect and possible clinical efficacy of in vivo inhibition of neutrophil extracellular traps by blockade of PI3K-gamma on the pathogenesis of microscopic polyangiitis.* Mod Rheumatol, 2018. **28**(3): p. 530-541.

62. Allen, C., et al., *Neutrophil cerebrovascular transmigration triggers rapid neurotoxicity through release of proteases associated with decondensed DNA.* J Immunol, 2012. **189**(1): p. 381-92.

63. Tarantino, E., et al., *Role of thromboxane-dependent platelet activation in venous thrombosis: Aspirin effects in mouse model.* Pharmacol Res, 2016. **107**: p. 415-25.

64. Tadie, J.M., et al., *HMGB1 promotes neutrophil extracellular trap formation through interactions with Toll-like receptor 4.* Am J Physiol Lung Cell Mol Physiol, 2013. **304**(5): p. L342-9.

65. Najmeh, S., et al., *Neutrophil extracellular traps sequester circulating tumor cells via beta1-integrin mediated interactions.* Int J Cancer, 2017. **140**(10): p. 2321-2330.

66. Cervantes-Luevano, K.E., et al., *Neutrophils drive type I interferon production and autoantibodies in patients with Wiskott-Aldrich syndrome.* J Allergy Clin Immunol, 2018.

67. Guglietta, S., et al., *Coagulation induced by C3aR-dependent NETosis drives protumorigenic neutrophils during small intestinal tumorigenesis.* Nat Commun, 2016. **7**: p. 11037.

68. Rossaint, J., et al., *Synchronized integrin engagement and chemokine activation is crucial in neutrophil extracellular trap-mediated sterile inflammation.* Blood, 2014. **123**(16): p. 2573-84.

69. Gaertner, F., et al., *Migrating Platelets Are Mechano-scavengers that Collect and Bundle Bacteria.* Cell, 2017. **171**(6): p. 1368-1382.e23.

70. Uotila, L.M., et al., *Filamin A Regulates Neutrophil Adhesion, Production of Reactive Oxygen Species, and Neutrophil Extracellular Trap Release.* J Immunol, 2017. **199**(10): p. 3644-3653.

71. Tamarozzi, F., et al., *Wolbachia endosymbionts induce neutrophil extracellular trap formation in human onchocerciasis.* Sci Rep, 2016. **6**: p. 35559.

72. Xu, D., et al., *Heparan Sulfate Modulates Neutrophil and Endothelial Function in Antibacterial Innate Immunity.* Infect Immun, 2015. **83**(9): p. 3648-56.

73. Sangaletti, S., et al., *Defective stromal remodeling and neutrophil extracellular traps in lymphoid tissues favor the transition from autoimmunity to lymphoma.* Cancer Discov, 2014. **4**(1): p. 110-29.

74. Sercundes, M.K., et al., *Targeting Neutrophils to Prevent Malaria-Associated Acute Lung Injury/Acute Respiratory Distress Syndrome in Mice.* PLoS Pathog, 2016. **12**(12): p. e1006054.

75. Sangaletti, S., et al., *Neutrophil extracellular traps mediate transfer of cytoplasmic neutrophil antigens to myeloid dendritic cells toward ANCA induction and associated autoimmunity.* Blood, 2012. **120**(15): p. 3007-18.

76. Akong-Moore, K., et al., *Influences of chloride and hypochlorite on neutrophil extracellular trap formation.* PLoS One, 2012. **7**(8): p. e42984.

77. Tripodo, C., et al., *Persistent immune stimulation exacerbates genetically-driven myeloproliferative disorders via stromal remodeling.* Cancer Res, 2017.

78. Al-Khafaji, A.B., et al., *Superoxide induces Neutrophil Extracellular Trap Formation in a TLR-4 and NOX-dependent mechanism.* Mol Med, 2016. **22**.

79. Yazdani, H.O., et al., *IL-33 exacerbates liver sterile inflammation by amplifying neutrophil extracellular trap formation.* J Hepatol, 2017.

80. Hopke, A., et al., *Neutrophil Attack Triggers Extracellular Trap-Dependent Candida Cell Wall Remodeling and Altered Immune Recognition.* PLoS Pathog, 2016. **12**(5): p. e1005644.

81. Zhang, S., et al., *Tanshinone IIA ameliorates chronic arthritis in mice by modulating neutrophil activities.* Clin Exp Immunol, 2017. **190**(1): p. 29-39.

82. Meng, W., et al., *Depletion of neutrophil extracellular traps in vivo results in hypersusceptibility to polymicrobial sepsis in mice.* Crit Care, 2012. **16**(4): p. R137.

83. Kothary, V., et al., *Group B Streptococcus Induces Neutrophil Recruitment to Gestational Tissues and Elaboration of Extracellular Traps and Nutritional Immunity.* Front Cell Infect Microbiol, 2017. **7**: p. 19.

84. Jin, L., S. Batra, and S. Jeyaseelan, *Diminished neutrophil extracellular trap (NET) formation is a novel innate immune deficiency induced by acute ethanol exposure in polymicrobial sepsis, which can be rescued by CXCL1.* PLoS Pathog, 2017. **13**(9): p. e1006637.

85. Papayannopoulos, V., et al., *Neutrophil elastase and myeloperoxidase regulate the formation of neutrophil extracellular traps.* J Cell Biol, 2010. **191**(3): p. 677-91.

86. Schreiber, A., et al., *Necroptosis controls NET generation and mediates complement activation, endothelial damage, and autoimmune vasculitis.* Proc Natl Acad Sci U S A, 2017. **114**(45): p. E9618-e9625.

87. Lima, T.F., et al., *Warifteine, an alkaloid purified from Cissampelos sympodialis, inhibits neutrophil migration in vitro and in vivo.* J Immunol Res, 2014. **2014**: p. 752923.

88. Raftery, M.J., et al., *beta2 integrin mediates hantavirus-induced release of neutrophil extracellular traps.* J Exp Med, 2014. **211**(7): p. 1485-97.

89. Ma, Y.H., et al., *High-mobility group box 1 potentiates antineutrophil cytoplasmic antibody-inducing neutrophil extracellular traps formation.* Arthritis Res Ther, 2016. **18**: p. 2.

90. Ma, F., et al., *Streptococcus suis Serotype 2 Biofilms Inhibit the Formation of Neutrophil Extracellular Traps.* Front Cell Infect Microbiol, 2017. **7**: p. 86.

91. Derre-Bobillot, A., et al., *Nuclease A (Gbs0661), an extracellular nuclease of Streptococcus agalactiae, attacks the neutrophil extracellular traps and is needed for full virulence.* Mol Microbiol, 2013. **89**(3): p. 518-31.

92. Hemmers, S., et al., *PAD4-mediated neutrophil extracellular trap formation is not required for immunity against influenza infection.* PLoS One, 2011. **6**(7): p. e22043.

93. Wang, Y., et al., *Mitochondrial Oxidative Stress Promotes Atherosclerosis and Neutrophil Extracellular Traps in Aged Mice.* Arterioscler Thromb Vasc Biol, 2017. **37**(8): p. e99-e107.

94. Leppkes, M., et al., *Externalized decondensed neutrophil chromatin occludes pancreatic ducts and drives pancreatitis.* Nat Commun, 2016. **7**: p. 10973.

95. Knight, J.S., et al., *Peptidylarginine deiminase inhibition is immunomodulatory and vasculoprotective in murine lupus.* J Clin Invest, 2013. **123**(7): p. 2981-93.

96. Knight, J.S., et al., *Peptidylarginine deiminase inhibition reduces vascular damage and modulates innate immune responses in murine models of atherosclerosis.* Circ Res, 2014. **114**(6): p. 947-56.

97. Subramanian, V., et al., *Design, synthesis, and biological evaluation of tetrazole analogs of Cl-amidine as protein arginine deiminase inhibitors.* J Med Chem, 2015. **58**(3): p. 1337-44.

98. Meng, H., et al., *In Vivo Role of Neutrophil Extracellular Traps in Antiphospholipid Antibody-Mediated Venous Thrombosis.* Arthritis Rheumatol, 2017. **69**(3): p. 655-667.

99. Papadaki, G., et al., *Neutrophil extracellular traps exacerbate Th1-mediated autoimmune responses in rheumatoid arthritis by promoting DC maturation.* Eur J Immunol, 2016. **46**(11): p. 2542-2554.

100. Stojkov, D., et al., *ROS and glutathionylation balance cytoskeletal dynamics in neutrophil extracellular trap formation.* J Cell Biol, 2017. **216**(12): p. 4073-4090.

101. Achouiti, A., et al., *Myeloid-related protein-14 contributes to protective immunity in gram-negative pneumonia derived sepsis.* PLoS Pathog, 2012. **8**(10): p. e1002987.

102. Bianchi, M., et al., *Restoration of anti-Aspergillus defense by neutrophil extracellular traps in human chronic granulomatous disease after gene therapy is calprotectin-dependent.* J Allergy Clin Immunol, 2011. **127**(5): p. 1243-52.e7.

103. Lefrancais, E., et al., *Maladaptive role of neutrophil extracellular traps in pathogen-induced lung injury.* JCI Insight, 2018. **3**(3).

104. Chen, S.T., et al., *CLEC5A is a critical receptor in innate immunity against Listeria infection.* Nat Commun, 2017. **8**(1): p. 299.

105. Bonne-Annee, S., et al., *Extracellular traps are associated with human and mouse neutrophil and macrophage mediated killing of larval Strongyloides stercoralis.* Microbes Infect, 2014. **16**(6): p. 502-11.

106. Sollberger, G., B. Amulic, and A. Zychlinsky, *Neutrophil Extracellular Trap Formation Is Independent of De Novo Gene Expression.* PLoS One, 2016. **11**(6): p. e0157454.

107. Ermert, D., et al., *Mouse neutrophil extracellular traps in microbial infections.* J Innate Immun, 2009. **1**(3): p. 181-93.

108. Abi Abdallah, D.S., et al., *Toxoplasma gondii triggers release of human and mouse neutrophil extracellular traps.* Infect Immun, 2012. **80**(2): p. 768-77.
